# Epidemiology of helminth infections in adults in rural rainforest communities of the Nkam-Wouri River Basin, Littoral Region of Cameroon: Implications for NTDs elimination using preventive chemotherapies

**DOI:** 10.1371/journal.pntd.0014780

**Published:** 2026-10-09

**Authors:** Anita Bate Obi Ebob, Narcisse Victor Tchamatchoua Gandjui, Benjamin Lenz, Emmanuel Ouam, Juluis Visnel Foyet, Amuam Andrew Beng, Fanny Fri Fombad, Franck Noel Nietcho, Manuel Ritter, Indulekha Karunakaran, Ndialle Nordine Mponge, Lucy Cho Nchang, Cheng Wang, Fonyuy Brandon Tahnteng, Jerome Fru Cho, Mathias Eyong Esum, Tatiana Djikeussi Katcho, Achim Hoerauf, Marc P. Hübner, Samuel Wanji

**Affiliations:** 1 Parasite and Vector Biology Research Unit, Department of Microbiology and Parasitology, Faculty of Science, University of Buea, Buea, Cameroon; 2 Research Foundation in Tropical Diseases and the Environment, Buea, Cameroon; 3 University of Bonn, University Hospital Bonn, Institute for Medical Microbiology, Immunology and Parasitology, Bonn, Germany; 4 German-West African Centre for Global Health and Pandemic Prevention (G-WAC), Bonn, Germany; 5 German Center for Infection Research (DZIF), Bonn-Cologne, Bonn, Germany; Zhejiang Wanli University, CHINA

## Abstract

**Background:**

Chronic helminth infections cause serious health problems; however, limited data on prevalence and distribution hinder effective control interventions. This study assessed the prevalence, distribution, and risk factors of helminth infections in adults across four health districts (HD) in the Littoral Region of Cameroon, while hypothesizing that helminth infection prevalences are significantly higher in densely forested and wetland forest plantation districts compared to degraded and mosaic plantation forest districts despite over more than two decades of preventive chemotherapy.

**Methodology/Principal Findings:**

Adults aged 18 – 65 years residing in the community for ≥ 5 years were recruited across four HDs. Skin snips, thick blood films, and stool samples were examined using the Kato Katz method (stool) and microscopy. Demographic data were analyzed in R. Participants were mainly farmers (79.9%), male (52.9%) and aged 18–49 years (50.7%). Yabassi HD, a densely forested area, recorded the highest prevalence of *Loa loa* and *Mansonella perstans*, while Loum HD, a wetland forest plantation area, showed higher prevalence for other helminth groups. Overall infection prevalence was 41.9%, dominated by filariasis (33.3%), with *Onchocerca volvulus* (23.9%) being most common, followed by *M. perstans* (11.9%) and *L. loa* (8.9%). Intestinal nematode prevalence was 9.9%, led by *Capillaria* spp. (4.6%), *Ascaris lumbricoides* (2.9%) and *Trichuris trichiura* (2.7%). Trematode infections included *Dicrocoelium dendriticum* (6.9%) and *Paragonimus* spp. (3.5%), while *Taenia* spp. (3.1%) was the most common cestode. Infections were more frequent in males and individuals over 40 years. Strong associations were observed among parasites with similar life cycles, Filariasis risk increased with age ≥ 50 years and longer residence, whereas recent deworming and higher education reduced the odds.

**Conclusions/Significance:**

Adult helminth infections pose a public health risk and may serve as transmission reservoirs in child-centred programs. Monitoring and including adults in preventive chemotherapy programs, promoting regular deworming and awareness and using a multi-parasite, multi-pathway strategy will strengthen control and elimination efforts in persistently endemic areas.

## Author summary

Helminths are parasitic worms affecting over 1.5 billion people globally. These infections lead to nutritional deficiencies, immune suppression, and increased vulnerability to other diseases. In Cameroon, only five helminth infections, ascariasis, trichuriasis, hookworm infections, schistosomiasis and onchocerciasis are currently being controlled, leaving a majority like loiasis, mansonellosis, taeniasis and others whose continuous presence interferes with the ongoing control interventions. Control efforts include annual Preventive Chemotherapy (PC) for schistosomiasis and soil-transmitted helminthiasis through school-based deworming, and Community Directed Treatment with Ivermectin (CDTI) for onchocerciasis. Currently, no vaccines are registered for human helminth infections, and the available drugs have limited efficacy. There is also limited or no data on disease prevalence and distribution. To achieve the WHO 2021–2030 road map goals for controlling helminth infections, robust surveillance and expanded interventions will be needed. Thus, this study aims to fill data gaps by assessing the prevalence, distribution, and risk factors of helminth infections in rural communities across four health districts in the Littoral Region of Cameroon.

## Introduction

Helminths are parasites that infect humans and other animals. Taxonomically, parasitic helminths comprise nematodes (roundworms) that include soil-transmitted helminths (STH) and filarial worms, as well as platyhelminths (flatworms) that include trematodes like schistosomes and cestodes such as tapeworms [1]. Intestinal helminth infections and tissue-dwelling nematodes are major public health concerns, particularly in developing countries. Globally, they affect an estimated 1.5 billion people [2], including 892 million with *Ascaris*, 508 million with *Trichuris*, 480 million with hookworms [3], and 240 million with *Schistosoma* [4]. The global burden of foodborne trematodiasis is estimated at 2.02 million DALYs [5], while taeniasis accounts for 2.8 million DALYs [6]. Tissue-dwelling nematodes, such as those responsible for filariasis, also warrant significant attention in developing countries. Globally, 485 million implementation units (IUs) require Mass Drug Administration (MDA) for lymphatic filariasis [7]. Onchocerciasis impacts an estimated 220 million individuals, with 14.6 million presenting with skin manifestations and 1.15 million suffering from vision impairment [8]. Loiasis affects approximately 42 million people in *Loa loa* endemic regions [9]. Meanwhile, mansonellosis is estimated to affect around 114 million people [10]. The World Health Organisation (WHO) categorises 7 of these diseases as Neglected Tropical Diseases: ascariasis, trichuriasis, hookworm infections, lymphatic filariasis, onchocerciasis, dracunculiasis, and schistosomiasis. In Cameroon, the most frequent geohelminths include *Ascaris lumbricoides*, hookworm spp, *Trichuris trichiura* [11], *Strongyloides stercoralis* [12], *Schistosoma mansoni* [13]*, Hymenolepis nana,* and *Taenia* species. The most prominent tissue-dwelling nematodes include the filarial nematodes *Onchocerca volvulus* [14], *L. loa* and *Mansonella perstans* [15]. Infections by these helminths usually lead to nutritional depletion and are associated with weakened immune responses and increased susceptibility to other infections. Associated risk factors are inadequate sanitation, lack of access to sanitation and infrastructure (Water, Sanitation and Hygiene (WASH)), eating raw or undercooked food, lack of regular deworming, prolonged exposure to vectors, lack of vector protective measures and outdoor activities [16–18]. Five NTDs are endemic in Cameroon: ascariasis, trichuriasis, hookworm infections, schistosomiasis, and onchocerciasis. Preventive Chemotherapy (PC) is the most widely used and effective intervention deployed to achieve the WHO 2021-2030 road map goals for these diseases. PC campaigns against schistosomiasis and STH have been integrated under the “Programme National de Lutte Contre la Schistosomiase et les Helminthiases Intestinales” (PNLSHI). Their core strategy is to conduct annual deworming of school-aged children at schools [11]. On the other hand, the Ministry of Public Health implements onchocerciasis control through the “National Program for the Control of Onchocerciasis and Filariasis” in Cameroon. Its core strategy is annual Community Directed Treatment with Ivermectin (CDTI). The WHO road map 2021-2030 targets include elimination as a public health problem in 96 (96%) countries for STH and in 78 (100%) countries for schistosomiasis, and an additional elimination of schistosomiasis from the human population in 30% of countries [19]. Meanwhile, for onchocerciasis, the goal is to eliminate the need for ivermectin MDA in at least 1 focus in 34 countries, in more than 50% of the population in at least 16 countries and the entire endemic population in at least 12 countries [19].

Presently, no vaccines are registered for human helminth infections, and the available drugs have limited efficacy. There is a risk of drug resistance and resurgence if MDA is stopped abruptly. As such, infection by helminths remains a public health concern, necessitating effective control to achieve the WHO road map 2021-2030. To achieve this goal, appropriate control interventions from local to international levels need to be put in place with proper monitoring and surveillance systems. However, data on the prevalence of these parasites are often incomplete or nonexistent. In addition, control measures may be less effective in some settings due to the geographic location and land cover, which may influence parasite dominance. As such, this study aims to provide information on the prevalence, distribution and factors associated with helminth infections in adults in rural communities along the Nkam-Wouri river drainage and rainy forest area of four health districts (HD) in the Littoral Region of Cameroon. It was hypothesised that helminth infection prevalences are significantly higher in densely forested and wetland forest plantation districts compared to degraded and mosaic plantation forest districts despite over two decades of preventive chemotherapy.

## Methods

### Ethical consideration

As part of the FIMMIP project [20], ethics approval to perform this study was obtained from the Cameroon National Ethics Committee for Human Health Research (No 2019/03/CE/CNERSH/SP). In Germany, ethical approval was obtained by the Ethics Committee for Clinical Human Research (Nr. 046/18). Written informed consent was obtained from each participant after ensuring that they understood and accepted their role in the study.

### Study site

The study was conducted from June to November 2021 in the Littoral region of Cameroon. The time span chosen was the rainy season in the study area, as it is the peak transmission season for these parasites. Four health districts (HDs) constituted the study area: Loum (4°42ˊ58˝N, 9°44′47˝E), Melong (5°7′16″N, 9°57′10″E), Yabassi (4°27′16″N, 9°57′56″E) and Manjo (4°50′40″N, 9°39′20″E) as shown in Fig 1. The selected health districts lie along the Nkam–Wouri River drainage and exhibit heterogeneous agro-forested landscapes, where banana, cocoa and oil palm plantations intermingle with remnant forest patches, wetlands, and expanding urban settlements; yet, while Loum is distinguished by its extensive wetlands and forest plantation, Manjo reflects a comparatively mosaic agro-forestry landscape with smaller-scale agriculture and forest remnants, Melong reflects a degraded forest under a growing urban pressure, and Yabassi remains predominantly densely forested, distinguished by the Ebo Forest block, where forest regrowth has offset agricultural decline. They also share an equatorial climate with two seasons. The inhabitants of these HDs are mainly involved in farming.

**Fig 1:**
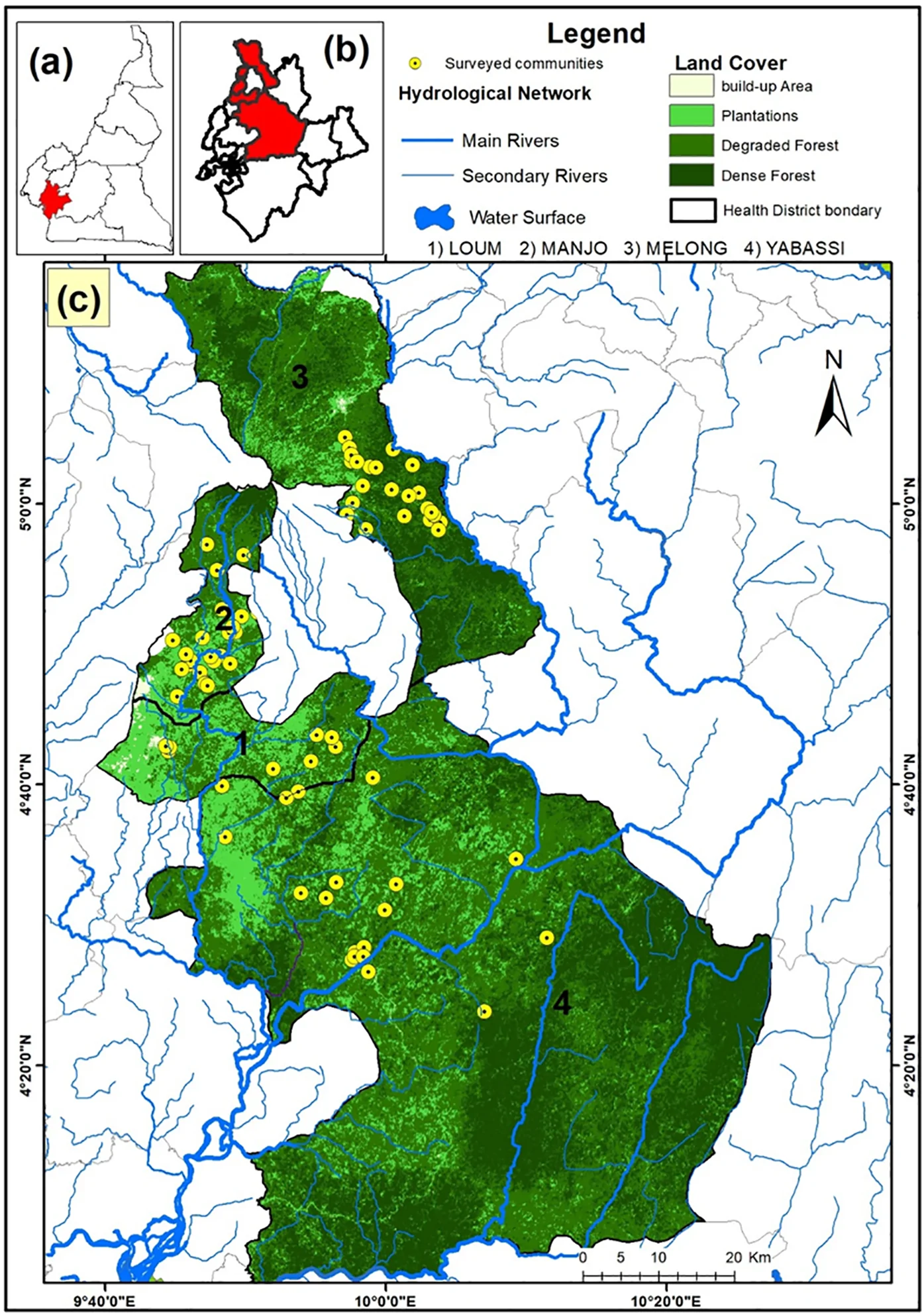
Map of study area. Littoral region of Cameroon depicting the health districts*,* communities, and land cover characteristics where the study was conducted. 1-Loum, 2-Manjo, 3-Melong, 4-Yabaassi. The image was generated using ArcGIS (ArcMap v10.5.5) software by Esri. The data used to plot the land cover map is Landsat 8, downloaded from the USGS website of NASA. Base shape files were reprinted under a *CC BY 4.0* license from the National Institute of Cartography, C*ameroon Source* and copyright information: https://services3.arcgis.com/dwv7XaFMgpPo7FSx/arcgis/rest/services/R%C3%A9gions/FeatureServer/0, https://data-minfof.opendata.arcgis.com/datasets/minfof::r%C3%A9gions/about.

The selection of HDs was guided by several factors, including the duration of ongoing PC programs, proximity to water bodies and forested areas. These districts have been engaged in Community-Directed Treatment with ivermectin (CDTI) for over two decades, beginning in 1999. PC chemotherapy for STH and schistosomiasis was introduced in 2004, with Yabassi, Manjo, and Melong HDs completing more than five effective rounds at coverage levels exceeding 75%. In contrast, Loum Health District reported STH prevalence below 20% and subsequently discontinued PC interventions

### Study design and population

The data in this study are part of baseline data for a larger cross-sectional cohort study investigating the immunological and metabolic profiles of helminth infections. The detailed methodology of the study has been published elsewhere [20]. Data was stratified per health district. For this, the sample size was calculated using the Cochran equation [21] below, yielding a minimum expected sample size of 385 participants to be screened in each health district.


$$\begin{array}{c} N=\frac{z^{\text{2}}.p(\text{1}-p)}{e^{\text{2}}} \end{array}$$

N represents the minimum sample size, z the z-score associated to the confidence level (1.96 for 95% confidence level), p the expected prevalence (assumed to be 50% to have an important sample size), and e the error margin, 5% in this study.

Study communities were purposively selected from high-risk areas near water bodies, forests and potential infectious and/or breeding sites. Participants in the communities were selected among willing residents who fulfilled the inclusion and exclusion criteria, which were age 18–65 years and residents of the communities for at least 5 years. The infected population consisted of participants with at least one helminth parasite, while the negative population were negative for all screened parasites. All those infected were treated with appropriate anthelmintic drugs in the follow-up study by our team, as planned in the FIMMIP protocol.

### Detection of intestinal helminth infections

Intestinal helminth infections were detected in stool samples using the Kato-Katz method. Briefly, a fresh stool sample was taken from each participant and processed using the Kato-Katz technique as described by Katz *et al*. in 1972 [22]. A single stool sample was collected from each participant; slides were prepared and examined independently by two experienced parasitologists. Results were computed as the number of eggs per gram of stool.

### Detection of filarial infections

#### O. volvulus.

**Nodule palpation**

The examination to determine the presence or absence of nodules followed the WHO standard protocol, as previously described [23]. Participants who consented were examined in a well-illuminated and enclosed room to ensure privacy [24]. The recorded data included the number of nodules and their specific locations, being marked on anatomical diagrams in the participant recruitment forms.

**Skin snipping**

The iliac crest of each participant was cleaned with cotton soaked in 70% alcohol and allowed to air-dry. Two bloodless skin biopsies were taken from the posterior left and right iliac crest using a sterile 2 mm corneo-scleral punch (CT 016 Everhards 2218-15 C, Meckenheim, Germany). Baneocin antibiotic powder was applied to the snipped areas to prevent infection. The two skin samples from each participant were placed in two separate, adjacent wells of a 96-well microtiter plate containing 100 µL of saline. The scleral punches were sterilized sequentially in sodium hypochlorite solution, 70% alcohol and distilled or normal saline. The plate was incubated overnight at room temperature to allow maximum emergence of microfilariae (mf) from the skin. To prevent spill-over or evaporation, the plates were sealed with paraﬁlm. Emerged mfwere counted using a light microscope at 10x magnification by two experienced microscopists the following day. The parasitaemia was expressed as average count per skin snip.

#### *L. loa* and *M. perstans.*

Diurnal blood collections were performed between 8 am - 4 pm. Approximately 50 µL of finger-prick blood was collected in a capillary tube and spread onto clean, labelled, and dry slides. The area covered by the smear was approximately 1.5 × 2.5 cm. The smears were allowed to air-dry and stained with 10% Giemsa using standard procedures. After staining, the smears were examined under a light microscope at 100 × magnification by trained technicians. The identification of parasites (*L. loa* and *M. perstans*) was based on microfilarial identification keys [25]. The quantification of mfwas expressed as microfilariae per millilitre (mf/mL) of blood.

### Questionnaire and anthropometric measurements

For each community surveyed, all subjects who voluntarily consented or whose parents or legal guardian agreed (for participants aged 18–20 years) and gave written informed consent to their participation in the study were asked to fill a structured questionnaire to obtain data on anthropometric and socio-demographics like sex, age, body mass index (BMI), level of education, occupation, duration in the community and last time point of deworming. The BMI was computed as weight in kilograms (kg) divided by the square of height in meters (m^2^). BMI was characterized as lean: BMI < 25 kg/m^2^, overweight: BMI [25–29] kg/m^2^ and obese: BMI ≥ 30 kg/m^2^.

### Data management

Confidentiality was ensured to safeguard the personal information of the study participants and other data, and such information was made accessible only to authorized personnel. All data collected in the paper Case Report Form (CRF) were entered electronically into the Research Electronic Data Capture (REDCap) software [26] and verified by double data entry. All statistical analyses were performed using R v4.4.0 [27].

### Statistical analysis

Frequency distributions were used to describe qualitative variables such as socio-demographic characteristics, helminth infection status (positive or negative), and deworming history. The results of this analysis were presented as absolute frequencies and percentages (the latter referred to as prevalence, i.e., the percentage of infected people among those tested), with associated 95% confidence intervals in some cases. To check the association between infection and deworming history, Pearson’s Chi-square test and Fisher’s exact test were used where appropriate. To identify socio-economic and epidemiological predictors of parasitic co-infection, defined as the presence of two or more parasites among individuals with at least one infection, Pearson’s chi-square test was used to check associations separately. Predictors that showed a significant association in the bivariate analysis were used for multiple logistic regression analysis.

To explore the association between various helminth infections, stepwise backward removal logistic regression was fitted iteratively with a threshold p-value of 20%. For each iteration, one helminth infection was set as the outcome variable, and all other infections were set as predictor variables before stepwise removal. The results were presented in a network diagram, with arrows showing significant associations. Negative associations were referred to as protective when two parasites were found together less often than expected, while positive associations were referred to as permissive when two parasites were found together more often than would be expected by chance, all without implying causation. Tables with more detailed results from these regressions were also provided. To identify determinants of helminth infection groups and filariae species, a stepwise backward elimination logistic regression was used, with sociodemographic and deworming intake history as predictor variables, and a threshold p-value of 20% set as well. The significance level in the whole analysis was set at 5%.

### GIS data source

The maps are an original work, designed and generated with ArcMap v10.5.5. The data used to plot the land cover map is Landsat 8, downloaded from the USGS website of NASA. National and administrative level 1 boundaries shapefiles were generated by the national institute of cartography and obtained through the data platform of the “Atlas Forestier du Cameroun” – Ministry of Forestry and Wildlife (https://data-minfof.opendata.arcgis.com/datasets/minfof::r%C3%A9gions/about), distributed under the Creative Commons Attribution 4.0 International (CC-BY) License (https://services3.arcgis.com/dwv7XaFMgpPo7FSx/arcgis/rest/services/R%C3%A9gions/FeatureServer/0). The health district limits shapefile was obtained from the Ministry of Public Health of Cameroon.

## Results

### Socio-demographic data

The study population consisted of 2,140 people, a slightly higher proportion of whom were males (52.9%). About one-third of the participants (29.5%) had lived in their respective communities between 5 and 10 years. A greater proportion of our study participants (42%) were recruited from the degraded forest landscape of Melong Health District (HD) followed by the dense forest of Yabassi (26%), the mosaic agro-forestry landscape of Manjo (21%), and the wetland-dominated Loum HD (9%). The age distribution was balanced, with 50.7% aged 18–49. More than half of the screened participants were classified as lean based on BMI (59.6%), with smaller percentages identified as overweight (28.1%) and obese (12.3%). The majority (79.9%) was engaged in agriculture. Educational levels varied, with nearly half of the population having only completed primary school (47.6%), while 1 in 20 people had higher education (4.9%). It’s worth noting that not all variables were collected from all enrolled participants for one reason or another (e.g., when a participant couldn’t recall an answer, or when the medical device software used during field activities got bugged). The socio-demographic data is summarized in Table 1.

**Table 1:** Socio-demographic characteristics of the study population.

Variables	Modalities	Total	N	Percentage (%)
Sex	Male	2,140	1,131	52.85
	Female		1,009	47.15
Age	18-49	2,140	1,085	50.70
	≥50		1,055	49.30
BMI	Lean: BMI < 25 kg/m2	2,113	1,260	59.63
	Overweight: BMI [25–30 kg/m2)		593	28.06
	Obese: BMI > 30 kg/m2		260	12.30
Education Level	No Formal education	473	44	9.26
	Primary education		226	47.57
	Secondary education		180	37.89
	Higher education		23	4.84
Occupation	farming	597	477	79.90
	others		120	20.10
Health District/ Land Cover	Loum/ wetlands	2,140	184	8.60
	Manjo/ mosaic landscape		447	20.89
	Melong/ degraded forest		898	41.96
	Yabassi/ dense forest		611	28.55
Last time point of deworming	Never	2,096	697	33.25
	Unknown		409	19.51
	<3 months		244	11.64
	4 – 6 months		175	8.35
	7 – 12 months		571	27.24
Duration in the community	0-10	2,135	630	29.51
	11-20		330	15.46
	21-30		297	13.91
	31-40		231	10.82
	41-50		251	11.76
	>50		396	18.55

### Prevalence of helminth infections

#### Overall prevalence of helminth infections in the study area.

A total of 2,071 skin snips, 2,133 thick blood films and 1,556 stool samples were examined to determine the infection status of the population. The overall prevalence of helminth infections in the study population was 41.6% with filariasis being the most common infection type in the study area (33.3%). *O. volvulus* was the most prevalent filarial parasite observed (23.9%), followed by *M. perstans* (11.9%) and *L. loa* (8.9%). Intestinal nematodes had an overall prevalence of 10%, with *Capillaria* spp*.* being the most prevalent (4.6%), followed by *A. lumbricoides* (2.9%) and *T. trichiura* (2.7%). *Dicrocoelium* *dendriticum* (6.9%) and *Paragonimus* spp. (3.5%) were the most prevalent trematode infections, while *Taenia* spp. (3.1%) was the most prevalent cestode infection. A detailed presentation of the overall prevalence of helminth infections is found in Table 2.

**Table 2:** Prevalence of helminth infections overall and per Health District (%).

Infection type	Overall		Health Districts n positive (prevalence)			
	n positive/ n tested	Prevalence (95% CI)	Loum +ve (%)	Manjo +ve (%)	Melong +ve (%)	Yabassi +ve (%)
**Filaria**						
*O. volvulus*	494/2071	23.85 (22.07 – 25.74)	56 (38.36)	79 (17.91)	187 (21.06)	172 (28.86)
*M. perstans*	253/2133	11.86 (10.56 – 13.30)	48 (26.23)	3 (0.67)	35 (3.9)	167 (27.51)
*L. loa*	190/2133	8.91 (7.77 – 10.19)	8 (4.37)	10 (2.25)	33 (3.67)	139 (22.9)
**Total**	**711/2137**	**33.27 (31.30 – 35.30)**	**84 (45.9)**	**86 (19.28)**	**231 (25.72)**	**310 (50.82)**
**Intestinal Nematodes**						
*Capillaria* spp.	71/1556	4.56 (3.63 – 5.72)	13 (10.08)	3 (1.12)	27 (4.23)	28 (5.38)
*A. lumbricoides*	45/1556	2.89 (2.17 – 3.85)	6 (4.65)	11 (4.1)	16 (2.5)	12 (2.31)
*T. trichiura*	42/1556	2.70 (2.00 – 3.63)	2 (1.55)	23 (8.58)	14 (2.19)	3 (0.58)
*E. vermicularis*	6/1556	0.39 (0.17 – 0.86)	4 (3.1)	0 (0)	1 (0.16)	1 (0.19)
*S. stercoralis*	2/1556	0.13 (0.03 – 0.51)	0 (0)	0 (0)	0 (0)	2 (0.38)
*Trichostrongylus* spp.	2/1556	0.13 (0.03 – 0.51)	0 (0)	0 (0)	0 (0)	2 (0.38)
Hookworm	1/1556	0.06 (0.01 – 0.46)	0 (0)	1 (0.37)	0 (0)	0 (0)
**Total**	**155/1556**	**9.96 (8.57 – 11.55)**	**19 (14.73)**	**36 (13.43)**	**54 (8.45)**	**46 (8.85)**
**Trematodes**						
*D. dendriticum*	107/1556	6.88 (5.72 – 8.25)	18 (13.95)	1 (0.37)	66 (10.33)	22 (4.23)
*Paragonimus* spp.	54/1556	3.47 (2.67 – 4.50)	6 (4.65)	3 (1.12)	30 (4.69)	15 (2.88)
*S. mansoni*	18/1556	1.16 (0.73 – 1.83)	3 (2.33)	7 (2.61)	4 (0.63)	4 (0.77)
*Fasciola* spp.	3/1556	0.19 (0.06 – 0.60)	0 (0)	1 (0.37)	2 (0.31)	0 (0)
*Clonorchis* spp.	1/1556	0.06 (0.01 – 0.46)	0 (0)	0 (0)	0 (0)	1 (0.19)
*Opisthorchis* spp.	1/1556	0.06 (0.01 – 0.46)	0 (0)	0 (0)	1 (0.16)	0 (0)
**Total**	**157/1556**	**10.09 (8.69 – 11.69)**	**26 (20.16)**	**11 (4.1)**	**84 (13.15)**	**36 (6.92)**
**Cestodes**						
*Taenia* spp.	48/1556	3.08 (2.33 – 4.07)	10 (7.75)	4 (1.49)	19 (2.97)	15 (2.88)
*D. caninum*	3/1556	0.19 (0.06 – 0.60)	1 (0.78)	0 (0)	0 (0)	2 (0.38)
*D. latum*	1/1556	0.06 (0.01 – 0.46)	0 (0)	0 (0)	0 (0)	1 (0.19)
*H. nana*	2/1556	0.13 (0.03 – 0.51)	0 (0)	1 (0.37)	0 (0)	1 (0.19)
**Total**	**53/1556**	**3.41 (2.61 – 4.43)**	**11 (8.53)**	**5 (1.87)**	**19 (2.97)**	**18 (3.46)**
**Overall Total**	**891/2140**	**41.64 (39.56 – 43.74)**	**105 (57.07)**	**122 (27.29)**	**306 (34.08)**	**358 (58.59)**

#### Prevalence of helminth infection by health district.

Filariasis exhibited a higher prevalence in the Yabassi HD (50.8%), with *M. perstans* (27.5%) and *L. loa* (22.9%) being the most prevalent species. *Capillaria* spp. was the most prevalent intestinal nematode, while *D. dendriticum* and *Taenia* spp. were the most prevalent trematodes and cestodes, respectively. Intestinal nematode (10.3%), trematode (20.2%), and cestode infections (8.5%) registered their highest prevalence in the Loum HD. *Capillaria* spp. (10.1%), *A. lumbricoides* (4.7%) and *E. vermicularis* (3.1%) were the most pervasive intestinal nematodes; *D. dendriticum* (13.9%) and *Paragonimus* spp. (4.7%) were the prevailing trematodes, and *Taenia* spp. (7.9%) was the most pervasive cestode infection. *O. volvulus* was the most abundant filaria (38.4%) in Loum HD. The Manjo HD recorded the highest prevalence of *T. trichiura* (8.6%), with *S. mansoni* being its most prevalent trematode (2.6%)*.* A detailed breakdown of helminth infection prevalence by health district is provided in Table 2 and S1 Appendix Table A-D.

#### Parasite prevalence and distribution by age and gender.

Generally, the older population (≥40 years) seemed to exhibit a higher prevalence of helminth infections. For filariae, the median age for *O. volvulus* was slightly above 50 years, whereas those for *L. loa* and *M. perstans* were slightly below 50 years. Intestinal nematodes were predominantly found in individuals aged 50 years and above, except for *E. vermicularis*. Depending on the species, trematode infections were observed in either older (*D. dendriticum, Paragonimus* spp.) or younger individuals (*S. mansoni, Fasciola* spp.). Apart from *D. caninum*, cestode infections were more common in those aged 18–49. Among the most prominent infections (n ≥ 18), men were more likely to harbor helminth infections than women across all parasite groups. A detailed breakdown of parasite prevalence and distribution by age and gender is displayed in Fig 2.

**Fig 2:**
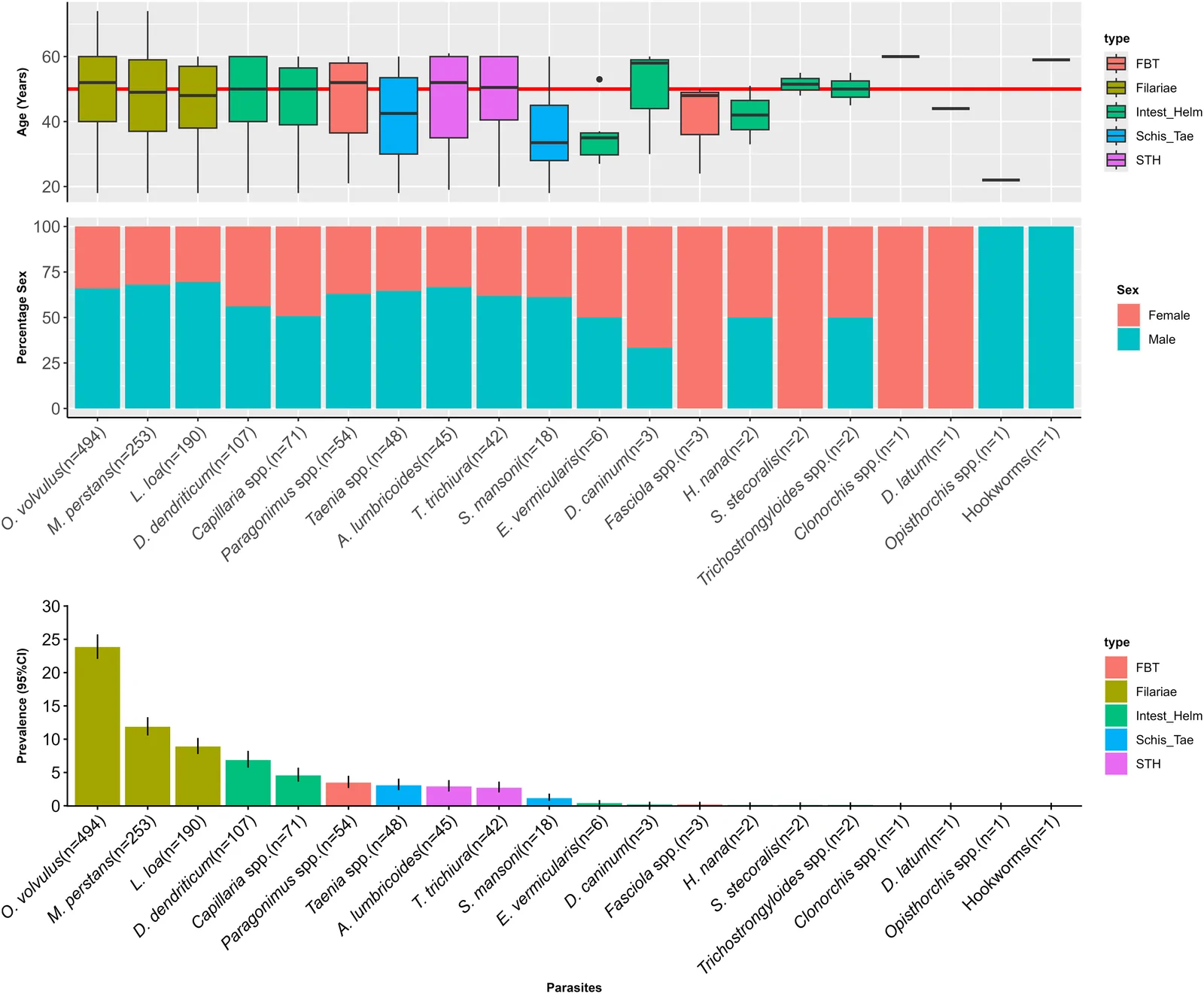
Parasite prevalence and distribution by age and gender. Comparisons were made by age (18-49 and >50 years (red line indicates age = 50)) and gender (male and female) for all the helminth parasites, and it was observed that adults >50 years exhibited a higher prevalence of helminth infections, and males were more likely to be infected.

### Description of helminth co-infections

Of 891 participants found infected with helminths, 568 (63.75%) were mono-infected, followed by co-infection with two helminth species (n = 218, 24.47%). The highest number of helminth co-infections was 5 helminth species found in 3 people (0.34%). More details are provided in Fig 3.

**Fig 3:**
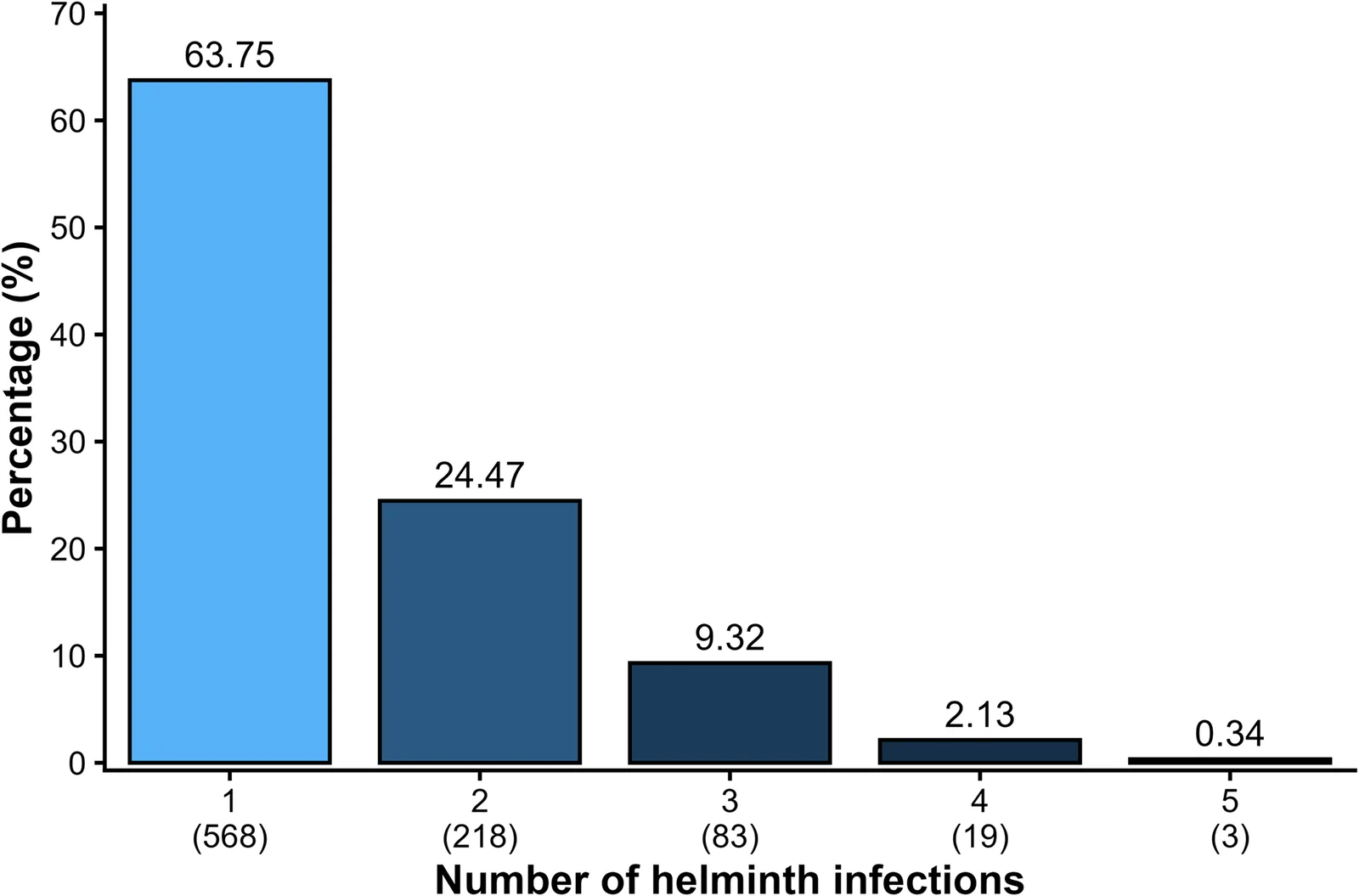
Co-infection distribution.

A total of 11 different pools of co-infection were established (filaria/trematode, filaria/cestode, filaria/intestinal nematode, filaria/intestinal nematode/trematode, filaria/intestinal nematode/cestode, filaria/trematode/cestode, filaria/intestinal nematode/trematode/cestode, cestode/trematode, intestinal nematode/trematode, intestinal nematode/cestode, intestinal nematode/trematode/cestode). Mono-infections were most prevalent for each helminth group compared to the corresponding co-infection counts (Fig 4). The median age for all cestode co-infection categories and the trematode/intestinal nematode pools was < 50 years, with a total of 60 cases. In contrast, the median age for filaria/trematode, filaria/intestinal nematode and filariae/intestinal nematode/trematode was above 50 years of age, with a total count of 94 cases. Across all co-infection categories, including filariae, more men were co-infected than women.

**Fig 4:**
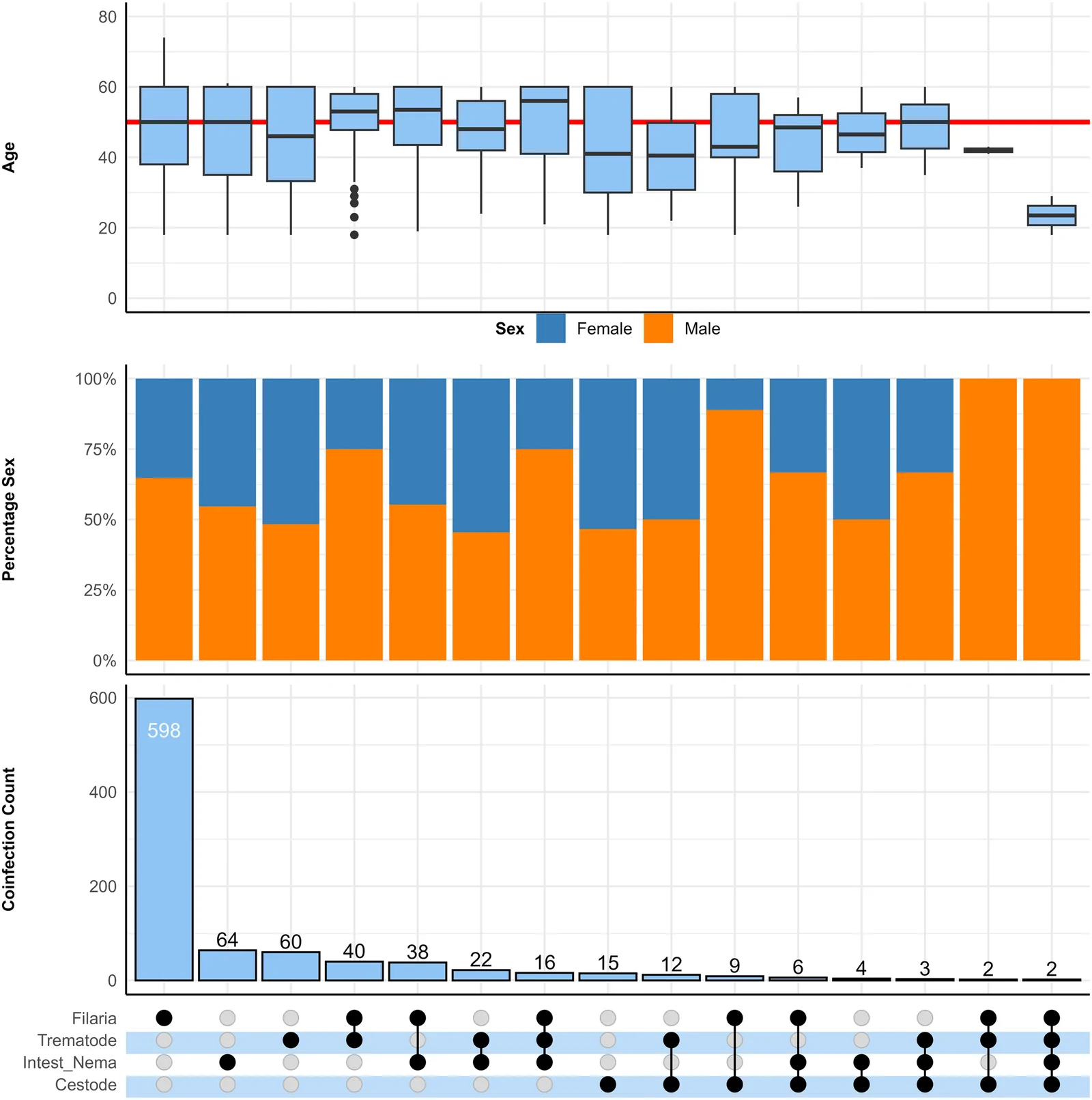
Co-infection distribution by age and gender.

### Co-infection dynamics and associations

Permissive association was used to refer to an increased likelihood of co-infection with two particular parasites/infection groups, without necessarily implying a causal link, whereas protective association referred to a reduced likelihood of co-infection with two particular parasites/infection groups. Adjusted odd ratios (aOR) quantified the strength of the association. There was a strong bidirectional permissive association among filarial infections, particularly between *L. loa* and *M. perstans* (aORs of 9.76 and 9.98, respectively). A bidirectional permissive association was also seen between *M. perstans* and *Paragonimus* spp. (aOR = 2.24, 2.14). Meanwhile, *L. loa* and *D. dendriticum* showed a bidirectional protective association (aORs = 0.14 and 0.12, respectively). However, globally, no associations were observed between filariasis and other infection groups. The intestinal nematodes, *A. lumbricoides* and *T. trichiura,* showed a permissive association for both models (aOR = 3.35, 3.48). *A. lumbricoides* also showed a bidirectional permissive association with *S. mansoni* (aOR = 4.90, 5.25) and a unidirectional association with *D. dendriticum* (aOR=2.61). *Capillaria* spp. showed bidirectional permissive associations with *Paragonimus* spp. (aOR = 2.34, 2.17), *D. dendriticum* (aOR = 8.13, 8.36) and *Taenia* spp. (aOR = 3.68, 3.74). More bidirectional associations are observed with *D. dendriticum* and *Paragonimus* spp. (aOR = 7.91, 9.03) as well as *Taenia* spp. (aOR = 3.34, 3.22). Globally, similar bidirectional permissive associations were observed with infections by intestinal nematodes, trematodes, and cestodes. In contrast, no association was observed between filariae and any of the three other parasitic groups (intestinal nematodes, cestodes and trematodes). Details of the association between helminth species and global representation are shown in Figs 5 and 6 and in S2 Appendix Table A.

**Fig 5:**
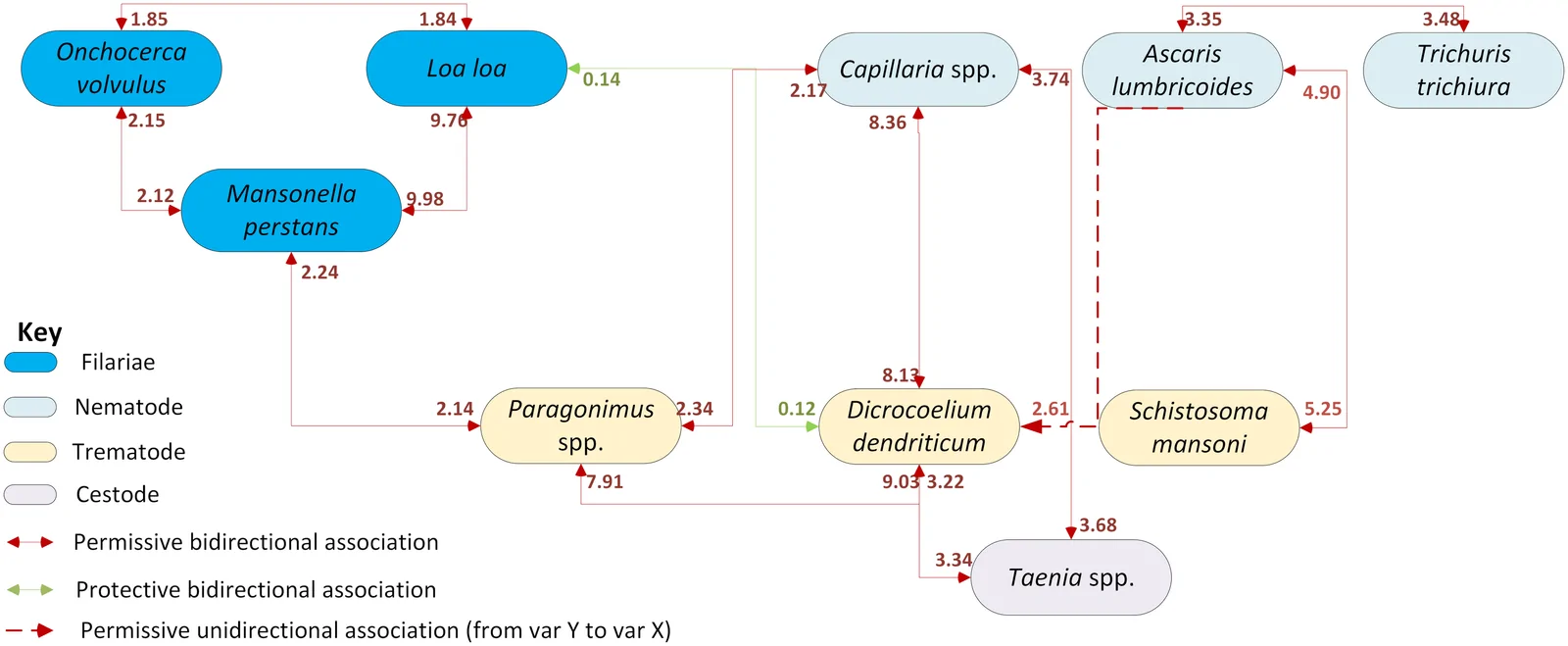
Association between parasitic infections. Arrows show the direction of interaction between parasites with the corresponding aOR close to arrows.

**Fig 6:**
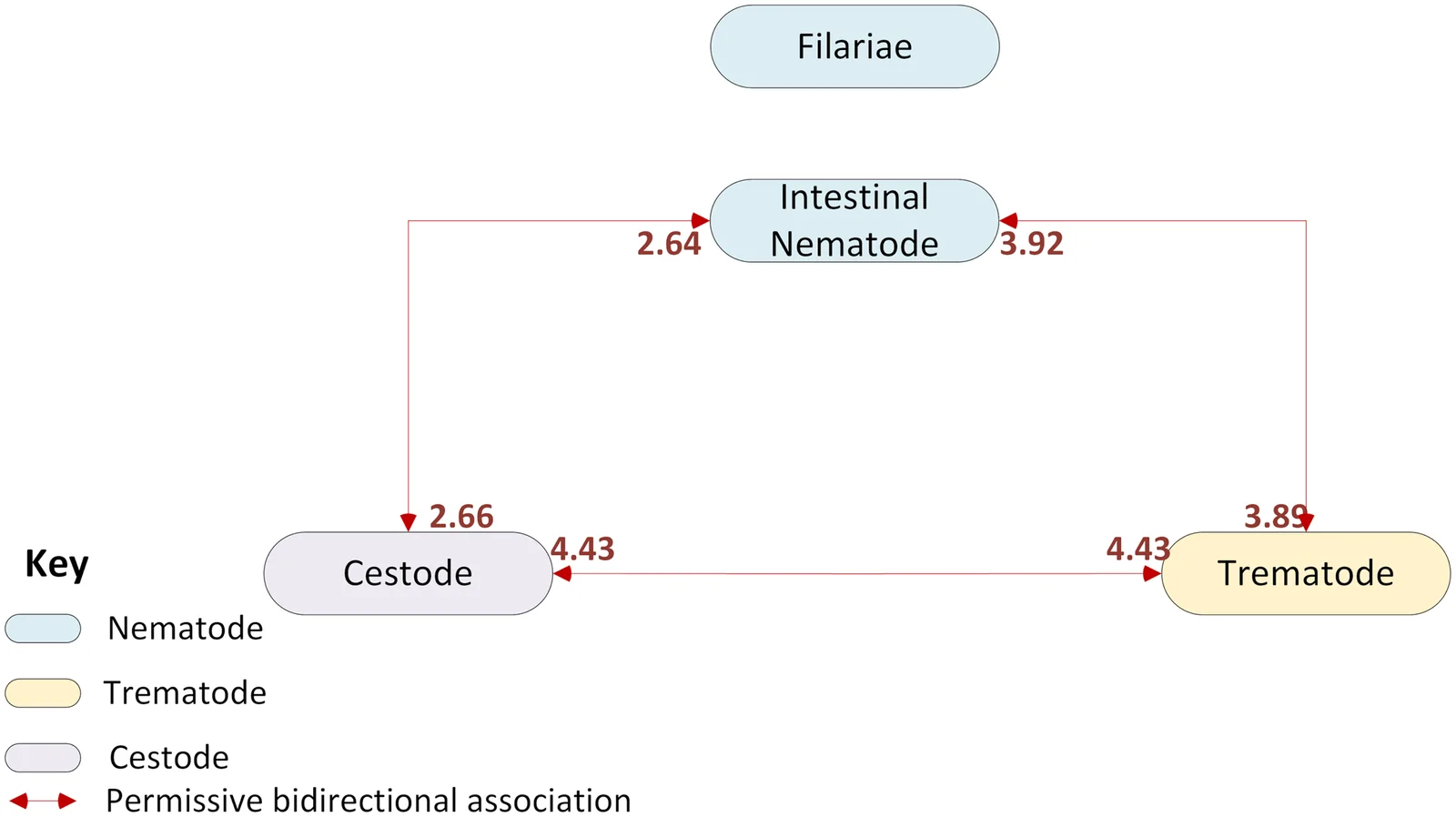
Association between parasitic infection groups. Arrows show the direction of interaction between parasites with the corresponding aOR close to arrow*.*

### Determinants of infections

#### Determinants of helminth infection groups.

**Filariasis**

Age ≥ 50 (aOR = 1.35, *p* = 0.014) was significantly associated with higher odds of infection compared with younger participants aged 18–49 years (S3 Appendix Table A-E). Duration in the community also showed a significant association with filarial infection, with longer residence associated with an increasingly higher risk of filariasis, peaking at 41–50 years of age (aOR = 2.33, *p* < 0.001). Being overweight was associated with significantly lower odds of infection (aOR = 0.51, *p* < 0.001) compared with being lean (S3 Appendix Table A-E). Similarly, last drug intake was linked to significantly lower odds of infection, with risk gradually increasing from those treated within 3 months (aOR = 0.5, *p* < 0.001) to those treated 4–6 (aOR = 0.62, *p* = 0.017) and 7–12 months ago (aOR = 0.66, *p* = 0.001). People who never took a deworming drug constituted the reference category.

**Cestodes**

Age ≥ 50 was associated with lower odds of infection (aOR = 0.62) compared with age 18–49, though the difference was not statistically significant (*p* = 0.106).

#### Determinants of filarial infection.

Level of education, especially secondary education as compared to no formal education, was significantly associated with lowered odds of being infected by either *M. perstans* (aOR = 0.2, *p =  0.004*), *O. volvulus* (aOR = 0.17), or *L. loa* (aOR = 0.06, *p* < 0.001), (S3 Appendix Table A-E)*.* Both Manjo HD (aOR = 0.6, p < 0.001), characterized by a mosaic agro-forestry landscape, and Melong HD (aOR = 0.3, p = 0.001), a degraded forest zone, showed significantly lower risk of *O. volvulus* infection. In contrast, Melong HD (aOR = 0.1, p = 0.001) alone exhibited lower odds of *M. perstans* infection. Meanwhile, Yabassi HD (aOR = 6.5, p = 0.004), distinguished by its dense forest cover, emerged as a high-risk zone for *L. loa* infection, all relative to Loum HD, which is dominated by wetland and plantation landscapes. For *M. perstans*, older age was associated with higher odds of infection (aOR = 2.18, *p* = 0.017) compared with those aged 18–49 years (S3 Appendix Table A-E). Finally, duration in community showed an increasing risk of infection with *O. volvulus* with a peak at 31 – 40 years (aOR = 6.41, *p* < 0.001, S3 Appendix Table A-E).

#### Determinants of co-infection.

Co-infection was more frequent among males (39.1%) than females (31.4%) in the bivariate analysis (p = 0.021); however, this difference was no longer significant after adjustment for other predictors. Adults aged ≥50 years appeared slightly more affected than those aged 18–49 years (37.4% vs. 35.0%), though the difference did not reach statistical significance.

Substantial heterogeneity was observed across the different land covers/ HD: Highest crude co-infection rates (47.6% and 45.5%) were recorded in the wetland-dominated Loum and densely forested Yabassi HDs respectively. In contrast, participants from Manjo mosaic agro-forestry landscape and Melong degraded forestry exhibited significantly lower odds compared with Loum (aOR = 0.22; 95% CI: 0.07–0.59; p = 0.004 and aOR = 0.41; 95% CI: 0.19–0.88; p = 0.023, respectively). Occupation also emerged as a significant predictor, with farmers showing the highest co-infection prevalence (47.6%) compared to only 20% among participants in other occupations (p < 0.001).

By contrast, education level, BMI, last time point of treatment, and duration of residence in the community were not significantly associated with co-infection. More details can be seen in S3 Appendix Table A-E.

## Discussion

The present study reports on the prevalence and distribution of helminth infections in adults, as well as the determinants of infection with these parasites.

Helminth infections were detected in 42% of the adult study population. Filarial nematodes emerged as the most common helminth infection, with an overall prevalence of 33%. Among the identified filarial species, *O. volvulus* was the most prevalent, accounting for 24% of cases. *O. volvulus* is the causative agent of onchocerciasis, which is primarily controlled through Community Directed Treatment with Ivermectin (CDTI), and has been ongoing in the study area for over 2 decades. Although it has been shown that 15 years of uninterrupted MDA is significantly associated with the elimination of transmission [28], the current prevalence in our study area highlights ongoing and persistent transmission. The reasons behind this persistence are probably multifactorial. Contributing factors may include low therapeutic coverage—often linked to farming activities—lack of awareness, and fear of side effects in *L. loa* co-endemic communities [29]. Overall, filariasis remains persistent in the study area, a situation likely sustained by the constant presence of its vectors. This could be attributed to the study area’s dense forest and fast-flowing rivers, linked to the Nkam-Wouri drainage, which support *Simulium* breeding (*O. volvulus* vectors) [30] and also favour *Chrysops* flies (*L. loa* vectors) [31]. Meanwhile, agricultural activities carried out by most residents promote moist, decomposing conditions ideal for *Culicoides* midges (*M. perstans* vectors) [15,32]. Thus, in areas with persistent transmission due to the ever-present vector, such as our study area, CDTI alone may be insufficient. As such, a more integrated method which includes vector control may be more valuable in controlling onchocerciasis*.*

*Capillaria spp.* and *D. dendriticum* emerged as the most frequently detected intestinal nematode and trematode infections, respectively, despite their typical predominance in animal hosts [33,34]. Among *Capillaria* species, only *C. philippinensis* is recognised as having humans as its definitive host, resulting in intestinal capillariasis [35]. Conversely, *C. hepatica* and *D. dendriticum* are rarely observed in humans, where they can cause liver disease. Therefore, the notable prevalence of these parasites in our study area may indicate incidental infections, likely resulting from ingestion of contaminated food or water or exposure linked to poor sanitation. In Cameroon, *Capillaria* has been isolated from dogs and cockroaches [36,37]. HIV was identified as a risk factor for infections with *D. dendriticum*, particularly HIV patients with high viral loads and compromised immunity, being more susceptible to *D. dendriticum* [38]. In the present study, *D. dendriticum* was linked to other helminth infections, such as *Paragonimus* spp., *Taenia* spp., and *Capillaria* spp., which may compromise immunity, thereby increasing susceptibility to *D. dendriticum* infection*.* In Cameroon, prevalent intestinal helminths usually include the intestinal nematodes *A. lumbricoides*, *T. trichiura* and hookworms, as well as trematodes (*Schistosoma* species)—parasites that present major NTDs. In this study, their prevalence, however, appeared relatively low compared to that in children from a 2011 survey carried out in the same region [13]. However, a lower infection prevalence among adults is anticipated, owing to increased awareness, improved hygiene practices, individual deworming initiatives, and potential overlap with other adult-targeted PC programs. In Cameroon, control efforts for STH and schistosomiasis focus primarily on children, leaving the adult population less covered. The interventions have been implemented for over two decades [39], and the Yabassi, Manjo, and Melong Health Districts have achieved more than five effective rounds of MDA (>75% PC coverage). Despite repeated interventions, these districts continue to report moderate prevalence levels among children [40]. The persistence of infections, coupled with current adult prevalence, suggests that transmission in younger populations may be sustained by adult reservoirs. Therefore, monitoring infection prevalence among adults remains essential, especially in areas where infections persist even after more than 20 years of PC control, because increased prevalence in this group would be particularly concerning, as adults may serve as infection reservoirs that can re-infect children and undermine child-focused control strategies. To further reduce parasite transmission and adult infection prevalence, awareness and regular personal deworming should be improved, and PC should be expanded to include adults where feasible.

*Taenia* species were the most frequently detected cestodes in our study, with a prevalence of 3%, surpassing the PC intervention threshold of ≥0.5% for human taeniasis. Infection with *T. solium* is known to cause neurocysticercosis—a condition implicated in up to 30% of epilepsy cases in endemic regions. In some communities, this association can reach as high as 70% [41]. Therefore, the recorded 3% prevalence likely signals a need for taeniasis control interventions within the study area. However, species-level identification was not possible due to limitations of the Kato-Katz technique. This human prevalence is slightly lower than the 8.4% infection rate reported in pigs in a study from Yaoundé, Cameroon, which also documented a nationwide prevalence of *T. solium* in pigs at 24.8% [42]. Both *T. saginata* and *T. solium* have been identified in stool samples from the Menoua West region, an area not very far from the present study site [43].

We examined the geographic distribution of helminth infections and assessed whether helminth infections could be influenced by location- and land cover characteristics. We observed that the Yabassi Health District (HD) exhibited the highest prevalence of filariasis, particularly *L. loa* and *M. perstans*. This may be attributed to its predominantly dense forests distinguished by the Ebo forest block and extensive plantation farming, which foster ideal breeding conditions for the *Chrysops* and *Culicoides* vectors. In contrast, *O. volvulus* was most prevalent in Loum HD, likely due to the presence of its abundant fast-flowing rivers such as the Dibombe and Mbette, which are favourable for *Simulium* vector breeding. Loum also recorded the highest rates of intestinal helminths, trematodes, and cestodes. Limited access to potable water in the area forces many residents to rely on its abundant rivers and springs, heightening the risk of waterborne infections. In addition, its mixed agro-forestry supports livestock rearing, which can facilitate transmission of foodborne cestodes through undercooked meat or contaminated produce; meanwhile, extensive plantation farming gives room for frequent soil contact and exposure to soil-transmitted helminths. Furthermore, human movement in Loum and Melong HDs, driven by tourism and their roles as transit hubs, may contribute to the spread of these infections. A study conducted in both urban and rural settings of the same region also showed that parasitic infection strongly depends on the environment/ land cover and the participants’ economic status [44].

We also assessed the distribution of helminth infections, both mono-infections and co-infections, by age and gender. Individuals over 40 years of age exhibited higher infection rates, especially for filarial infections, in which an age ≥ 50 years was significantly associated with an increased risk of filariasis, particularly *Mansonella perstans* (OR=2.18, p = 0.017). A comparable age-related increase in helminth disease prevalence has been documented in Cameroon [45–47]. One possible explanation is immunosenescence, the gradual weakening of the immune system with age, making older individuals more susceptible to parasitic infections [48]. In line with this, a longer stay in the community was linked to a higher risk of filarial infection, especially for *O. volvulus*. This increased vulnerability may result from cumulative exposure to infected vectors over time or repeated reinfection from consistent sources of transmission [49]. Helminth infections were generally higher among men, possibly because they have greater exposure through outdoor activities such as farming, hunting, and fishing, unlike women, who tend to engage in predominantly indoor tasks. However, these findings diverge from those of Mbuh *et al*. (2012) and Yamssi *et al*. (2021), who reported higher infection rates of *A. lumbricoides*, *T. trichiura*, hookworm, and *Capillaria* among women, suggesting that regional or cultural influences may play a role [50,51]. Higher education level, notably secondary school education, was associated with lower odds of infection, likely due to improved hygiene and health awareness associated with formal education. As such, emphasis should be placed on promoting hygiene education and disease awareness to improve parasite control. Finally, recent drug intake was strongly associated with a reduced risk for filariasis, with individuals treated within the last 3 months (OR = 0.5, p < 0.001) showing the lowest infection risk. This may underscore the effectiveness of PC programs. However, it is unclear whether this reflects the impact of CDTI alone or in combination with personal deworming efforts, as treatment timing was based on self-reporting rather than documented records. The duration and frequency of intake were also not captured. Still, Wanji *et al*. have shown that oral declaration is reliable [52]. The lack of significant associations across other helminth groups may stem from low public awareness, driven by a lack of corresponding MDA initiatives targeting adults. Therefore, we can once again infer that strengthening community health education and encouraging routine individual deworming could be pivotal in advancing helminth control efforts.

While parasitic co-infections are widespread, their direct interactions—particularly regarding disease progression—remain poorly understood. To investigate potential relationships among helminth infections, we analysed associations that may pose a trend for future experimental or longitudinal studies. Insights into these interactions may contribute to the development of more suitable treatment and control strategies. Our analysis revealed several significant associations, with permissive relationships occurring more frequently than protective ones, underscoring the high prevalence of co-infections in developing regions. We observed permissive associations between filariae, notably between *M. perstans* and *L. loa*, aligning with past findings in which *M. perstans* microfilarial load was reported to significantly predict the intensity of *L. loa* infection [53]. Additionally, *M. perstans* demonstrated a bidirectional permissive association with *Paragonimus* species. Previous studies suggest that *M. perstans* may manipulate immune regulation and dampen inflammatory responses [10,54] and may also influence the progression of other diseases, such as malaria, tuberculosis, and possibly paragonimiasis, which may thrive in similar environments. Among intestinal helminths, *A. lumbricoides* and *T. trichiura* exhibited a permissive association but showed no statistically significant linkage with *Capillaria* species. This pattern may be explained by the fact that *A. lumbricoides* and *T. trichiura* are both STHs as defined by WHO, sharing similar transmission routes and life cycles [54], unlike *Capillaria*, which has varied transmission routes, often involving foodborne or indirect environmental exposure rather than direct soil contact. Previous research indicates that helminths often form positive associations with other parasites that thrive under comparable ecological conditions [55]. Interestingly, *D. dendriticum* demonstrated the widest range of interactions with other helminth species in this analysis, notably a strong permissive association with *Capillaria spp*. (aOR = 8.36) and *Paragonimus spp*. (aOR = 7.91). Despite sharing a few biological or ecological similarities with *D. dendriticum*, the underlying mechanisms behind these associations remain unclear. Further investigation is needed to determine whether such co-infections exert synergistic or antagonistic effects on disease progression. Given the high cost of targeting individual parasite species, leveraging co-infection dynamics may present an opportunity for integrated control approaches. Strategies that target multiple parasites and various stages of their life cycles, such as a vector-integrated approach and the development of new drug candidates with pan-nematode efficacy [56–58], could offer a more efficient and sustainable solution.

The study had a few limitations, such as the diagnostic methods used. The Kato-Katz technique [22] used to detect intestinal helminths is known to underestimate the prevalence of both single and multiple helminth infections, particularly when only a single stool sample is analysed and in areas with low-intensity infections [59–61]. It is not suitable for clearly differentiating closely related species, such as *Capillaria* and *Taenia*, in our case, and has low sensitivity for *Enterobius* infections. Similarly, microscopic detection of the parasites is limited in diagnosing the pre-patent stage of infection [62]. Consequently, the true prevalence of helminth infections was likely underestimated. The study relied on oral declaration of deworming, but the duration and frequency of deworming were not accounted for. As such, the true impact of personal deworming may be limited. The sample size target was not reached for the Loum HD; as such, the data here may not be truly representative. Children were excluded from the study. However, for inference purpose, data on children were obtained from the ESPEN website [40].

In conclusion, we have successfully outlined the current epidemiological situation of helminth parasites among adults in our study area. We observed that the prevalence of helminth infections among adults in the study area is still a public health concern and have shown that the prevalence of these helminth infections differs significantly by health district. We noted that it would be important to monitor adult prevalence for child-centred helminth control programs, as integrating the adult population into PC interventions in areas with persistent prevalence may be crucial. Our findings confirm that prevalence varies significantly across health districts, influenced by environmental and land-cover characteristics, with infection being more severe in densely forested and wetland areas, underscoring the need for geographically tailored interventions. In these areas, strengthening current interventions or using an integrated strategy that addresses multiple parasites and/or transmission pathways would offer a more impactful approach to helminth elimination by combining multiple anthelmintics and vector control in a single PC program. More importantly, our work also calls for public policy measures to increase awareness and improve hygiene and sanitation infrastructure in the affected regions to limit transmission, eliminate vector breeding grounds, and prevent secondary infections.

## Supporting information

S1 Appendix Prevalence of different helminth groups per community. (DOCX)

S2 Appendix Association between parasitic infections. (DOCX)

S3 Appendix Determinants of helminth infections. (DOCX)

S4 Appendix Fig A data. (DOCX)

S5 Appendix Fig A data. (XLSX)

## References

[1] Brindley PJ, Mitreva M, Ghedin E, Lustigman S. Helminth genomics: The implications for human health. PLoS Negl Trop Dis. 2009;3(10):e538. doi: 10.1371/journal.pntd.0000538 19855829 PMC2757907

[2] Chen J, Gong Y, Chen Q, Li S, Zhou Y. Global burden of soil-transmitted helminth infections, 1990–2021. Infect Dis Poverty. 2024;13(1). doi: 10.1186/s40249-024-01238-9 39444032 PMC11515461

[3] World Health Organization WHO. Soil-transmitted helminth infections. 2023 [cited 2024 August 23]. Available from: https://www.who.int/news-room/fact-sheets/detail/soil-transmitted-helminth-infections

[4] World Health Organization WHO. Schistosomiasis and soil-transmitted helminthiases: progress report. Weekly epidemiological record Relevé épidémiologique hebdomadaire. 2022;97(48):621–32.

[5] Torgerson PR, Devleesschauwer B, Praet N, Speybroeck N, Willingham AL, Kasuga F. World Health Organization Estimates of the Global and Regional Disease Burden of 11 Foodborne Parasitic Diseases, 2010: A Data Synthesis. PLoS Med. 2015;12(12):e1001920. doi: 10.1371/journal.pmed.1001920 26633705 PMC4668834

[6] World Health Organization. Taeniasis/cysticercosis. WHO website. 2022 [cited 2024 August 23]. Available from: https://www.who.int/news-room/fact-sheets/detail/taeniasis-cysticercosis

[7] World Health Organization. Lymphatic filariasis, status of mass drug administration. WHO website. 2024 [cited 2024 August 23]. Available from: https://www.who.int/data/gho/data/themes/topics/lymphatic-filariasis

[8] World Health Organization. Onchocerciasis 2022. WHO website. 2022 [cited 2024 August 2]. Available from: https://www.who.int/news-room/fact-sheets/detail/onchocerciasi

[9] Ramharter M, Butler J, Mombo-Ngoma G, Nordmann T, Davi SD, Zoleko Manego R. The African eye worm: current understanding of the epidemiology, clinical disease, and treatment of loiasis. Lancet Infect Dis. 2024;24(3):e165–78. doi: 10.1016/S1473-3099(23)00438-3 37858326

[10] Ritter M, Hsu HY, Lenz B, Kien CA, Gandjui NVT, Hübner MP, et al. *Mansonella perstans* - the forgotten filaria. Trends Parasitol. 2025;41(10):909–21. doi: 10.1016/j.pt.2025.08.003 40908246

[11] Ministry of Public Health R of C. The National Programme for the Control of Schistosomiasis and Soil-transmitted Helminthiasis. 2016 [cited 2024 August 25]. Available from: https://pnlshi.org/achievements/

[12] Mang K, Leticia D. Distribution of infectious eggs and larvae of helminths in stools and soil in the medical district of Awae in the city of Yaounde, Cameroon. Universal Journal of Life and Environmental Sciences. 2024;6(1):158–65.

[13] Tchuem Tchuenté L-A, Dongmo Noumedem C, Ngassam P, Kenfack CM, Gipwe NF, Dankoni E, et al. Mapping of schistosomiasis and soil-transmitted helminthiasis in the regions of Littoral, North-West, South and South-West Cameroon and recommendations for treatment. BMC Infect Dis. 2013;13:602. doi: 10.1186/1471-2334-13-602 24365046 PMC3878270

[14] Tekle AH, Zouré HGM, Noma M, Boussinesq M, Coffeng LE, Stolk WA, et al. Progress towards onchocerciasis elimination in the participating countries of the African Programme for Onchocerciasis Control: epidemiological evaluation results. Infect Dis Poverty. 2016;5(1):66. doi: 10.1186/s40249-016-0160-7 27349645 PMC4924267

[15] Wanji S, Tayong DB, Ebai R, Opoku V, Kien CA, Ndongmo WPC, et al. Update on the biology and ecology of *Culicoides* species in the South-West region of Cameroon with implications on the transmission of *Mansonella perstans*. Parasit Vectors. 2019;12(1):166. doi: 10.1186/s13071-019-3432-9 30975194 PMC6460808

[16] Centre for Disease Control and Prevention. About onchocerciasis. CDC website. 2024 [cited 2024 April 26]. Available from: https://www.cdc.gov/filarial-worms/about/onchocerciasis.html

[17] Tofel HK, Abongwa LE, Ndifor RF, Ntonifor HN. Intestinal helminthiasis, anaemia and associated risk factors in a cross-section of the population of Melong, Littorial Region of Cameroon. Sci Rep. 2024;14(1):649. doi: 10.1038/s41598-023-46446-9 38182648 PMC10770123

[18] Badia-Rius X, Betts H, Wanji S, Molyneux D, Taylor MJ, Kelly-hope LA. Environmental factors associated with Loa loa microfilaria prevalence and intensity in diverse bioecological zones of Cameroon. Frontiers in Tropical Diseases. 2021;2(668641):1–10. doi: 10.3389/fitd.2021.668641

[19] World Health Organization. Ending the neglect to attain the sustainable development goals: a road map for neglected tropical diseases 2021–2030. WHO. 2020.

[20] Lenz B, Andrew BA, Ritter M, Karunakaran I, Gandjui NVT, Nchang LC, et al. The design and development of a study protocol to investigate *Onchocerca volvulus, Loa loa* and *Mansonella* perstans-mediated modulation of the metabolic and immunological profile in lean and obese individuals in Cameroon. PLoS One. 2023;18(6):e0285689. doi: 10.1371/journal.pone.0285689 37267236 PMC10237473

[21] Cochran WG. Sampling techniques. 2nd ed. John Wiley & Sons. 1963. Available from: https://books.google.cm/books?id=Y-SxXwAACAAJ

[22] Katz N, Chaves A, Pellegrino J. A simple device for quantitative stool thick-smear technique in *Schistosomiasis mansoni*. Rev Inst Med Trop Sao Paulo. 1972;14(6):397–400. 4675644

[23] Wanji S, Kengne-Ouafo JA, Esum ME, Chounna PWN, Tendongfor N, Adzemye BF, et al. Situation analysis of parasitological and entomological indices of onchocerciasis transmission in three drainage basins of the rain forest of South West Cameroon after a decade of ivermectin treatment. Parasit Vectors. 2015;8:202. doi: 10.1186/s13071-015-0817-2 25886166 PMC4393872

[24] Zouré HGM, Wanji S, Noma M, Amazigo UV, Diggle PJ, Tekle AH, et al. The geographic distribution of Loa loa in Africa: results of large-scale implementation of the Rapid Assessment Procedure for Loiasis (RAPLOA). PLoS Negl Trop Dis. 2011;5(6):e1210. doi: 10.1371/journal.pntd.0001210 21738809 PMC3125145

[25] Orihel TC, Lowrie RC Jr. Loa loa: development to the infective stage in an American deerfly, *Chrysops atlanticus*. Am J Trop Med Hyg. 1975;24(4):610–5. doi: 10.4269/ajtmh.1975.24.610 1057379

[26] Harris PA, Taylor R, Minor BL, Elliott V, Fernandez M, O’Neal L, et al. The REDCap consortium: Building an international community of software platform partners. J Biomed Inform. 2019;95:103208. doi: 10.1016/j.jbi.2019.103208 31078660 PMC7254481

[27] R Core Team. R: A Language and Environment for Statistical Computing. Vienna, Austria: R Foundation for Statistical Computing. 2024.

[28] Mutono N, Basáñez M-G, James A, Stolk WA, Makori A, Kimani TN, et al. Elimination of transmission of onchocerciasis (river blindness) with long-term ivermectin mass drug administration with or without vector control in sub-Saharan Africa: a systematic review and meta-analysis. Lancet Glob Health. 2024;12(5):e771–82. doi: 10.1016/S2214-109X(24)00043-3 38484745 PMC11009120

[29] Kamga G-R, Dissak-Delon FN, Nana-Djeunga HC, Biholong BD, Ghogomu SM, Souopgui J, et al. Audit of the community-directed treatment with ivermectin (CDTI) for onchocerciasis and factors associated with adherence in three regions of Cameroon. Parasit Vectors. 2018;11(1):356. doi: 10.1186/s13071-018-2944-z 29921312 PMC6009063

[30] Renz A. Programme onchocercoses/ Simulium breeding sites. 2021 [cited 2024 May 18]. Available from: https://www.riverblindness.eu/onchocerciasis/simulium-vectors/simulium-breedings-sites/

[31] Badia-Rius X, Betts H, Molyneux DH, Kelly-Hope LA. Environmental factors associated with the distribution of Loa loa vectors Chrysops spp. in Central and West Africa: seeing the forest for the trees. Parasites & Vectors. 2019;12(1):1–11. doi: 10.1186/s13071-019-3327-9 30728063 PMC6366063

[32] Wanji S, Tayong D, Ebai R, Opoku V, Kien C, Ndongmo W, et al. Update on the biology and ecology of *Culicoides* species in the South West region of Cameroon with implications on the transmission of *Mansonella perstans*. 10.1186/s13071-019-3432-9 PMC6460808 30975194

[33] Mellor PS, Boorman J, Baylis M. Culicoides biting midges: their role as arbovirus vectors. Annu Rev Entomol. 2000;45:307–40. doi: 10.1146/annurev.ento.45.1.307 10761580

[34] Junquera P. *Capillaria* spp, parasitic hairworms of dogs and cats. Biology, prevention and control. Eucoleus, Pearsonema, Aonchotheca. 2022 [cited 2024 August 2]. Available from: https://parasitipedia.net/index.php?option=com_content&view=article&id=2606&Itemid=2888

[35] Centre for Disease Control and Prevention. Dicrocoeliasis. CDC website. 2019 [cited 2024 May 20]. Available from: https://www.cdc.gov/dpdx/dicrocoeliasis/index.html

[36] Centre for Disease Control and Prevention. Capilliariasis. 2024 [cited 2024 May 20]. Available from: https://www.cdc.gov/capillaria/about/index.html

[37] Komtangi MC, Mpoame M, Payne VK, Ngufor N. Prevalence of gastrointestinal helminths of dogs in Dschang, Cameroon. Journal of the Cameroon Academy of Sciences. 2005;5(1):11–4.

[38] Atiokeng Tatang RJ, Tsila HG, Wabo Poné J. Medically Important Parasites Carried by Cockroaches in Melong Subdivision, Littoral, Cameroon. J Parasitol Res. 2017;2017:7967325. doi: 10.1155/2017/7967325 28912965 PMC5585542

[39] Tankoua-Tchounda R, Nack J, Nchetnkou CM, Tchankwé DLK, Lontsi-Demano M, Essangui E, et al. Enteric Parasitosis of HIV-positive Patients in Cameroon: A Case of the Regional Hospital of Bafoussam (Western Region). IJTDH. 2022;:6–18. doi: 10.9734/ijtdh/2022/v43i241374

[40] The Expanded Special Project for the Elimination of Neglected Tropical Diseases, ESPEN. Cameroon/ESPEN. Soil-transmitted helminthiasis. Cameroon/ESPEN. 2024 [cited 2026 April 21]. Available from: https://espen.afro.who.int/maps-data/countries/cameroon#sth

[41] World Health Organization. Taeniasis/cysticercosis. 2022 [cited 2024 April 25]. Available from: https://www.who.int/news-room/fact-sheets/detail/taeniasis-cysticercosis

[42] Assana E, Awah-Ndukum J, Djonmaïla JD, Zoli AP. Prevalence of porcine *Taenia solium* and Taenia hydatigena cysticercosis in Cameroon. Prev Vet Med. 2019;169:104690. doi: 10.1016/j.prevetmed.2019.104690 31311631

[43] Vondou L, Zoli AP, Nguekam, Pouedet S, Assana E, Kamga Tokam AC, et al. La taeniose/cysticercose à *Taenia Solium* dans la Menoua (Ouest-Cameroun). Parasite. 2002;9(3):271–4. doi: 10.1051/parasite/2002093271 12375371

[44] Nchang LC, Magha C, Fonong PA, Gandjui NVT, Tchatat NM, Nkimbeng DA, et al. Parasitic infection prevalence in tuberculosis patients and their household contacts in the Littoral Region of Cameroon. Parasite Epidemiol Control. 2025;28:e00409. doi: 10.1016/j.parepi.2025.e00409 39867582 PMC7617333

[45] Wanji S, Tendongfor N, Esum M, Ndindeng S, Enyong P. Epidemiology of concomitant infections due to Loa loa, *Mansonella perstans*, and *Onchocerca volvulus* in rain forest villages of Cameroon. Med Microbiol Immunol. 2003;192(1):15–21. doi: 10.1007/s00430-002-0154-x 12592559

[46] Tatuene KJ, Fotsing GR, Mbougua TJ, Djeunga NH. Epidemiology of *Loa Loa* and *Mansonella Perstans* Filariasis in the Akonolinga Health District, Centre Region, Cameroon. Health Sci Dis. 2014;15(1):1–5. doi: 10.5281/hsd.v15i1.297

[47] Tofel HK, Abongwa LE, Ndifor RF, Ntonifor HN. Intestinal helminthiasis, anaemia and associated risk factors in a cross-section of the population of Melong, Littorial Region of Cameroon. Sci Rep. 2024;14(1):649. doi: 10.1038/s41598-023-46446-9 38182648 PMC10770123

[48] Quiros-Roldan E, Sottini A, Natali PG, Imberti L. The Impact of Immune System Aging on Infectious Diseases. Microorganisms. 2024;12(4):775. doi: 10.3390/microorganisms12040775 38674719 PMC11051847

[49] Das PK, Srividya A, Pani SP, Ramaiah KD, Vanamail P, Dhanda V. Cumulative exposure and its relationship with chronic filarial disease in bancroftian filariasis. Southeast Asian J Trop Med Public Health. 1994;25(3):516–21. 7777918

[50] Mbuh JV, Ntonifor NH, Ojong J. The epidemiology of soil-transmitted helminth and protozoan infections in south-west Cameroon. J Helminthol. 2012;86(1):30–7. doi: 10.1017/S0022149X1000091X 21281528

[51] Yamssi C, Noumedem Anangmo CN, Payne VK, Sabi MB, Ngangnang GR. Gastrointestinal Nematodes among Residents in Melong, Moungo Division, Littoral Region, Cameroon. Biomed Research International. 2021. doi: 10.1155/2021/5368973 33628786 PMC7886586

[52] Wanji S, Kengne-Ouafo JA, Esum ME, Chounna PWN, Adzemye BF, Eyong JEE, et al. Relationship between oral declaration on adherence to ivermectin treatment and parasitological indicators of onchocerciasis in an area of persistent transmission despite a decade of mass drug administration in Cameroon. Parasit Vectors. 2015;8:667. doi: 10.1186/s13071-015-1283-6 26715524 PMC4696282

[53] Drame PM, Montavon C, Pion SD, Kubofcik J, Fay MP, Nutman TB. Molecular epidemiology of blood-borne human parasites in a Loa loa-, *Mansonella perstans*-, and plasmodium falciparum-endemic region of Cameroon. American Journal of Tropical Medicine and Hygiene. 2016;94(6):1301–8. doi: 10.4269/ajtmh.15-0746 27044568 PMC4889748

[54] Kajero OT, Janoušková E, Bakare EA, Belizario V, Divina B, Alonte AJ, et al. Co-infection of intestinal helminths in humans and animals in the Philippines. Trans R Soc Trop Med Hyg. 2022;116(8):727–35. doi: 10.1093/trstmh/trac002 35169848 PMC9356178

[55] Donohue RE, Cross ZK, Michael E. The extent, nature, and pathogenic consequences of helminth polyparasitism in humans: A meta-analysis. PLoS Negl Trop Dis. 2019;13(6):e0007455. doi: 10.1371/journal.pntd.0007455 31211774 PMC6599140

[56] Krücken J, Holden-Dye L, Keiser J, Prichard RK, Townson S, Makepeace BL, et al. Development of emodepside as a possible adulticidal treatment for human onchocerciasis-The fruit of a successful industrial-academic collaboration. PLoS Pathog. 2021;17(7):e1009682. doi: 10.1371/journal.ppat.1009682 34293063 PMC8297762

[57] Hübner MP, Martin C, Specht S, Koschel M, Dubben B, Frohberger SJ, et al. Oxfendazole mediates macrofilaricidal efficacy against the filarial nematode *Litomosoides sigmodontis* in vivo and inhibits *Onchocerca* spec. motility in vitro. PLoS Negl Trop Dis. 2020;14(7):e0008427. doi: 10.1371/journal.pntd.0008427 32628671 PMC7365463

[58] Risch F, Kazakov A, Specht S, Pfarr K, Fischer PU, Hoerauf A, et al. The long and winding road towards new treatments against lymphatic filariasis and onchocerciasis. Trends Parasitol. 2024;40(9):829–45. doi: 10.1016/j.pt.2024.07.005 39122645

[59] Booth M, Vounatsou P, N’goran EK, Tanner M, Utzinger J. The influence of sampling effort and the performance of the Kato-Katz technique in diagnosing *Schistosoma mansoni* and hookworm co-infections in rural Côte d’Ivoire. Parasitology. 2003;127(Pt 6):525–31. doi: 10.1017/s0031182003004128 14700188

[60] Knopp S, Mgeni AF, Khamis IS, Steinmann P, Stothard JR, Rollinson D, et al. Diagnosis of soil-transmitted helminths in the era of preventive chemotherapy: effect of multiple stool sampling and use of different diagnostic techniques. PLoS Negl Trop Dis. 2008;2(11):e331. doi: 10.1371/journal.pntd.0000331 18982057 PMC2570799

[61] Kongs A, Marks G, Verlé P, Van der Stuyft P. The unreliability of the Kato-Katz technique limits its usefulness for evaluating *S. mansoni* infections. Trop Med Int Health. 2001;6(3):163–9. doi: 10.1046/j.1365-3156.2001.00687.x 11299032

[62] Alvar J, Alves F, Bucheton B, Burrows L, Büscher P, Carrillo E, et al. Implications of asymptomatic infection for the natural history of selected parasitic tropical diseases. Semin Immunopathol. 2020;42(3):231–46. doi: 10.1007/s00281-020-00796-y 32189034 PMC7299918

