# Supplementary material for: Epidemiology of helminth infections in adults in rural rainforest communities of the Nkam-Wouri River Basin, Littoral Region of Cameroon: Implications for NTDs elimination using preventive chemotherapies
Source: PLoS Negl Trop Dis. 2026 Oct 9;20(10):e0014780. doi: 10.1371/journal.pntd.0014780 (PMC13686460; doi:10.1371/journal.pntd.0014780)
Supplement: Supplementary file 1 [file pntd.0014780.s001.docx]

# S1 Appendix: Prevalence of helminth infection groups per community and per health district

**Table A: Prevalence of filariasis per community**

| **Community Name** | ***L. loa*** | | ***M. perstans*** | | ***O. volvulus*** | | **Filariae** | |
| --- | --- | --- | --- | --- | --- | --- | --- | --- |
|  | **x/n** | **Prevalence (CI95)** | **x/n** | **Prevalence (CI95)** | **x/n** | **Prevalence (CI95)** | **x/n** | **Prevalence (CI95)** |
| **Health District (HD): Loum**  **Health Area (HA): Babong** | | | | | | | | |
| Badjoki | 2/35 | 5.71 (1.43 – 20.16) | 14/35 | 40 (25.32 – 56.73) | 14/23 | 60.87 (40.24 – 78.23) | 18/35 | 51.43 (35.3 – 67.26) |
| Mabombe | 1/34 | 2.94 (0.41 – 18.14) | 0/34 | 0 (0 – NaN) | 5/13 | 38.46 (16.98 – 65.64) | 6/34 | 17.65 (8.15 – 34.1) |
| Ngondo | 2/16 | 12.5 (3.14 – 38.6) | 9/16 | 56.25 (32.38 – 77.54) | 7/15 | 46.67 (24.09 – 70.7) | 12/16 | 75 (49.18 – 90.29) |
| Nkongbissi/Salaka | 0/37 | 0 (0 – NaN) | 24/37 | 64.86 (48.45 – 78.38) | 15/36 | 41.67 (26.91 – 58.08) | 30/37 | 81.08 (65.31 – 90.7) |
| Overall Babong HA | 5/122 | 4.1 (1.72 – 9.47) | 47/122 | 38.52 (30.32 – 47.43) | 41/87 | 47.13 (36.91 – 57.59) | 66/122 | 54.1 (45.22 – 62.72) |
| **Health District (HD): Loum**  **Health Area (HA): Loum 2** | | | | | | | | |
| Bonabela | 1/9 | 11.11 (1.54 – 49.9­9) | 0/9 | 0 (0 – NaN) | 4/9 | 44.44 (17.68 – 74.87) | 4/9 | 44.44 (17.68 – 74.87) |
| Bonkeng | 0/20 | 0 (0 – NaN) | 0/20 | 0 (0 – NaN) | 3/20 | 15 (4.92 – 37.58) | 3/20 | 15 (4.92 – 37.58) |
| Dimithe | 2/32 | 6.25 (1.57 – 21.81) | 1/32 | 3.12 (0.44 – 19.11) | 8/30 | 26.67 (13.93 – 44.96) | 11/32 | 34.38 (20.16 – 52.07) |
| Overall Loum 2 HA | 3/61 | 4.92 (1.59 – 14.17) | 1/61 | 1.64 (0.23 – 10.73) | 15/59 | 25.42 (15.95 – 37.99) | 18/61 | 29.51 (19.45 – 42.05) |
| Overall Loum HD | 8/183 | 4.37 (2.2 – 8.5) | 48/183 | 26.23 (20.37 – 33.08) | 56/146 | 38.36 (30.83 – 46.48) | 84/183 | 45.9 (38.82 – 53.16) |
| **Health District (HD): Manjo**  **Health Area (HA): Kolla** | | | | | | | | |
| Kolla Carrefour | 0/1 | 0 (0 – NaN) | 0/1 | 0 (0 – NaN) | 0/1 | 0 (0 – NaN) | 0/1 | 0 (0 – NaN) |
| Kolla Lala | 0/7 | 0 (0 – NaN) | 0/7 | 0 (0 – NaN) | 2/7 | 28.57 (7.2 – 67.34) | 2/7 | 28.57 (7.2 – 67.34) |
| Kolla Songo | 0/4 | 0 (0 – NaN) | 0/4 | 0 (0 – NaN) | 1/5 | 20 (2.72 – 69.1) | 1/5 | 20 (2.72 – 69.1) |
| Lala Mission | 0/24 | 0 (0 – NaN) | 0/24 | 0 (0 – NaN) | 3/22 | 13.64 (4.46 – 34.79) | 3/24 | 12.5 (4.09 – 32.38) |
| Manengoteng 1 | 0/9 | 0 (0 – NaN) | 0/9 | 0 (0 – NaN) | 2/9 | 22.22 (5.6 – 57.9) | 2/9 | 22.22 (5.6 – 57.9) |
| Manengoteng 2 | 3/54 | 5.56 (1.8 – 15.86) | 2/54 | 3.7 (0.93 – 13.64) | 8/54 | 14.81 (7.59 – 26.93) | 10/54 | 18.52 (10.26 – 31.11) |
| Mantem 1 | 0/3 | 0 (0 – NaN) | 0/3 | 0 (0 – NaN) | 2/3 | 66.67 (15.35 – 95.66) | 2/3 | 66.67 (15.35 – 95.66) |
| Overall Kolla HA | 3/102 | 2.94 (0.95 – 8.72) | 2/102 | 1.96 (0.49 – 7.5) | 18/101 | 17.82 (11.53 – 26.52) | 20/103 | 19.42 (12.88 – 28.19) |
| **Health District (HD): Manjo**  **Health Area (HA): Manjo 1** | | | | | | | | |
| Boum-Quartier 8 | 1/18 | 5.56 (0.78 – 30.65) | 0/18 | 0 (0 – NaN) | 3/18 | 16.67 (5.47 – 40.86) | 3/18 | 16.67 (5.47 – 40.86) |
| Emeng | 0/1 | 0 (0 – NaN) | 0/1 | 0 (0 – NaN) | 0/1 | 0 (0 – NaN) | 0/1 | 0 (0 – NaN) |
| Manjo Centre | 0/1 | 0 (0 – NaN) | 0/1 | 0 (0 – NaN) | 1/1 | 100 (NaN – NaN) | 1/1 | 100 (NaN – NaN) |
| Manjo-Etam | 0/1 | 0 (0 – NaN) | 0/1 | 0 (0 – NaN) | 1/1 | 100 (NaN – NaN) | 1/1 | 100 (NaN – NaN) |
| Manjo-Quartier 1 | 0/47 | 0 (0 – NaN) | 1/47 | 2.13 (0.3 – 13.62) | 9/46 | 19.57 (10.51 – 33.51) | 10/47 | 21.28 (11.85 – 35.21) |
| Manjo-Quartier 2 | 1/30 | 3.33 (0.47 – 20.2) | 0/30 | 0 (0 – NaN) | 4/30 | 13.33 (5.1 – 30.59) | 5/30 | 16.67 (7.11 – 34.32) |
| Manjo-Quartier 3 | 0/11 | 0 (0 – NaN) | 0/11 | 0 (0 – NaN) | 3/11 | 27.27 (9.05 – 58.57) | 3/11 | 27.27 (9.05 – 58.57) |
| Manjo-Quartier 4 | 0/8 | 0 (0 – NaN) | 0/8 | 0 (0 – NaN) | 0/8 | 0 (0 – NaN) | 0/8 | 0 (0 – NaN) |
| Namba | 0/31 | 0 (0 – NaN) | 0/31 | 0 (0 – NaN) | 8/31 | 25.81 (13.46 – 43.74) | 8/31 | 25.81 (13.46 – 43.74) |
| Overall Manjo 1 HA | 2/148 | 1.35 (0.34 – 5.24) | 1/148 | 0.68 (0.1 – 4.64) | 29/147 | 19.73 (14.07 – 26.95) | 31/148 | 20.95 (15.13 – 28.25) |
| **Health District (HD): Manjo**  **Health Area (HA): Manjo 2** | | | | | | | | |
| Manewang | 0/18 | 0 (0 – NaN) | 0/18 | 0 (0 – NaN) | 0/18 | 0 (0 – NaN) | 0/18 | 0 (0 – NaN) |
| Manjo-Quartier 7 | 0/1 | 0 (0 – NaN) | 0/1 | 0 (0 – NaN) | 0/1 | 0 (0 – NaN) | 0/1 | 0 (0 – NaN) |
| Mantem 2 | 0/9 | 0 (0 – NaN) | 0/9 | 0 (0 – NaN) | 0/9 | 0 (0 – NaN) | 0/9 | 0 (0 – NaN) |
| Mikombe-Q4 | 0/29 | 0 (0 – NaN) | 0/29 | 0 (0 – NaN) | 3/29 | 10.34 (3.37 – 27.6) | 3/29 | 10.34 (3.37 – 27.6) |
| Ndiang | 2/36 | 5.56 (1.39 – 19.67) | 0/36 | 0 (0 – NaN) | 3/35 | 8.57 (2.79 – 23.44) | 5/36 | 13.89 (5.9 – 29.32) |
| Ngolsi | 0/8 | 0 (0 – NaN) | 0/8 | 0 (0 – NaN) | 3/8 | 37.5 (12.54 – 71.52) | 3/8 | 37.5 (12.54 – 71.52) |
| Overall Manjo 2 HA | 2/101 | 1.98 (0.5 – 7.57) | 0/101 | 0 (0 – NaN) | 9/100 | 9 (4.75 – 16.4) | 11/101 | 10.89 (6.13 – 18.6) |
| **Health District (HD): Manjo**  **Health Area (HA): N'Lohe** | | | | | | | | |
| N'lohe | 2/23 | 8.7 (2.18 – 28.88) | 0/23 | 0 (0 – NaN) | 5/23 | 21.74 (9.35 – 42.8) | 5/23 | 21.74 (9.35 – 42.8) |
| Overall N’lohe HA | 2/23 | 8.7 (2.18 – 28.88) | 0/23 | 0 (0 – NaN) | 5/23 | 21.74 (9.35 – 42.8) | 5/23 | 21.74 (9.35 – 42.8) |
| **Health District (HD): Manjo**  **Health Area (HA): Njoumbeng** | | | | | | | | |
| Badjoungue | 0/9 | 0 (0 – NaN) | 0/9 | 0 (0 – NaN) | 1/8 | 12.5 (1.73 – 53.73) | 1/9 | 11.11 (1.54 – 49.99) |
| Bigmop | 0/30 | 0 (0 – NaN) | 0/30 | 0 (0 – NaN) | 7/30 | 23.33 (11.55 – 41.5) | 7/30 | 23.33 (11.55 – 41.5) |
| Mouetouk | 0/15 | 0 (0 – NaN) | 0/15 | 0 (0 – NaN) | 3/15 | 20 (6.59 – 46.98) | 3/15 | 20 (6.59 – 46.98) |
| Njoumbeng | 1/17 | 5.88 (0.82 – 32.03) | 0/17 | 0 (0 – NaN) | 7/17 | 41.18 (21.04 – 64.78) | 8/17 | 47.06 (25.54 – 69.73) |
| Overall Njoumbeng HA | 1/71 | 1.41 (0.2 – 9.33) | 0/71 | 0 (0 – NaN) | 18/70 | 25.71 (16.84 – 37.17) | 19/71 | 26.76 (17.77 – 38.19) |
|  | 10/445 | 2.25 (1.21 – 4.13) | 3/445 | 0.67 (0.22 – 2.07) | 79/441 | 17.91 (14.61 – 21.78) | 86/446 | 19.28 (15.88 – 23.21) |
| **Health District (HD): Melong**  **Health Area (HA): Barehock** | | | | | | | | |
| Barehock | 3/100 | 3 (0.97 – 8.89) | 2/100 | 2 (0.5 – 7.64) | 19/99 | 19.19 (12.59 – 28.14) | 22/100 | 22 (14.95 – 31.16) |
| Ebouh | 1/23 | 4.35 (0.61 – 25.22) | 0/23 | 0 (0 – NaN) | 3/23 | 13.04 (4.27 – 33.55) | 4/23 | 17.39 (6.68 – 38.23) |
| Esseh | 1/8 | 12.5 (1.73 – 53.73) | 0/8 | 0 (0 – NaN) | 0/8 | 0 (0 – NaN) | 1/8 | 12.5 (1.73 – 53.73) |
| Mbangla | 1/11 | 9.09 (1.26 – 43.86) | 1/11 | 9.09 (1.26 – 43.86) | 5/11 | 45.45 (20.28 – 73.19) | 6/11 | 54.55 (26.81 – 79.72) |
| Mbouele Kem | 1/44 | 2.27 (0.32 – 14.45) | 1/44 | 2.27 (0.32 – 14.45) | 3/44 | 6.82 (2.22 – 19.11) | 5/44 | 11.36 (4.81 – 24.54) |
| Ngol | 4/54 | 7.41 (2.81 – 18.13) | 0/54 | 0 (0 – NaN) | 7/51 | 13.73 (6.69 – 26.1) | 10/54 | 18.52 (10.26 – 31.11) |
| Overall Barehock HA | 11/240 | 4.58 (2.56 – 8.09) | 4/240 | 1.67 (0.63 – 4.36) | 37/236 | 15.68 (11.58 – 20.89) | 48/240 | 20 (15.41 – 25.54) |
| **Health District (HD): Melong**  **Health Area (HA): Melong 2** | | | | | | | | |
| Bayon | 1/62 | 1.61 (0.23 – 10.57) | 0/62 | 0 (0 – NaN) | 13/61 | 21.31 (12.8 – 33.33) | 14/62 | 22.58 (13.85 – 34.6) |
| Eboukou | 0/9 | 0 (0 – NaN) | 0/9 | 0 (0 – NaN) | 1/8 | 12.5 (1.73 – 53.73) | 1/9 | 11.11 (1.54 – 49.99) |
| Ekom-Nkam | 2/69 | 2.9 (0.73 – 10.86) | 0/69 | 0 (0 – NaN) | 8/69 | 11.59 (5.91 – 21.51) | 9/69 | 13.04 (6.93 – 23.21) |
| Longko | 0/2 | 0 (0 – NaN) | 0/2 | 0 (0 – NaN) | 1/2 | 50 (5.89 – 94.11) | 1/2 | 50 (5.89 – 94.11) |
| Melong 2 | 2/68 | 2.94 (0.74 – 11.01) | 0/68 | 0 (0 – NaN) | 13/67 | 19.4 (11.61 – 30.61) | 13/68 | 19.12 (11.44 – 30.2) |
| Melong Q3 | 0/21 | 0 (0 – NaN) | 0/21 | 0 (0 – NaN) | 2/20 | 10 (2.51 – 32.38) | 2/21 | 9.52 (2.39 – 31.13) |
| Melong Q4 | 0/32 | 0 (0 – NaN) | 0/32 | 0 (0 – NaN) | 5/32 | 15.62 (6.66 – 32.47) | 5/32 | 15.62 (6.66 – 32.47) |
| Soundop | 1/40 | 2.5 (0.35 – 15.73) | 1/40 | 2.5 (0.35 – 15.73) | 9/40 | 22.5 (12.14 – 37.88) | 9/40 | 22.5 (12.14 – 37.88) |
| Overall Melong 2 HA | 6/303 | 1.98 (0.89 – 4.34) | 1/303 | 0.33 (0.05 – 2.3) | 52/299 | 17.39 (13.5 – 22.11) | 54/303 | 17.82 (13.91 – 22.54) |
| **Health District (HD): Melong**  **Health Area (HA): Ndom Bakem** | | | | | | | | |
| Mandjibo | 1/37 | 2.7 (0.38 – 16.85) | 2/37 | 5.41 (1.36 – 19.2) | 17/37 | 45.95 (30.81 – 61.87) | 17/37 | 45.95 (30.81 – 61.87) |
| Mbarembeng | 6/85 | 7.06 (3.21 – 14.83) | 3/85 | 3.53 (1.14 – 10.38) | 29/85 | 34.12 (24.85 – 44.78) | 35/85 | 41.18 (31.25 – 51.88) |
| Mbie | 1/32 | 3.12 (0.44 – 19.11) | 6/32 | 18.75 (8.67 – 35.92) | 7/32 | 21.88 (10.8 – 39.3) | 12/32 | 37.5 (22.68 – 55.1) |
| Mounko | 3/41 | 7.32 (2.38 – 20.37) | 1/41 | 2.44 (0.34 – 15.39) | 11/40 | 27.5 (15.93 – 43.16) | 14/41 | 34.15 (21.38 – 49.72) |
| Mpaka | 4/35 | 11.43 (4.36 – 26.77) | 3/35 | 8.57 (2.79 – 23.44) | 7/35 | 20 (9.85 – 36.4) | 12/35 | 34.29 (20.61 – 51.18) |
| Ndom | 1/56 | 1.79 (0.25 – 11.61) | 4/56 | 7.14 (2.71 – 17.54) | 13/55 | 23.64 (14.25 – 36.57) | 16/56 | 28.57 (18.3 – 41.67) |
| Ndouenbot | 0/6 | 0 (0 – NaN) | 2/6 | 33.33 (8.39 – 73.19) | 2/6 | 33.33 (8.39 – 73.19) | 4/6 | 66.67 (26.81 – 91.61) |
| Ndouenke | 0/7 | 0 (0 – NaN) | 4/7 | 57.14 (22.98 – 85.63) | 2/7 | 28.57 (7.2 – 67.34) | 4/7 | 57.14 (22.98 – 85.63) |
| Nkonia Koniama | 0/4 | 0 (0 – NaN) | 0/4 | 0 (0 – NaN) | 0/4 | 0 (0 – NaN) | 0/4 | 0 (0 – NaN) |
| Nkoniambot | 0/10 | 0 (0 – NaN) | 3/10 | 30 (9.98 – 62.37) | 1/10 | 10 (1.39 – 46.72) | 4/10 | 40 (15.83 – 70.26) |
| Nkonianke | 0/37 | 0 (0 – NaN) | 2/37 | 5.41 (1.36 – 19.2) | 6/37 | 16.22 (7.47 – 31.69) | 8/37 | 21.62 (11.2 – 37.63) |
| Ntangtom | 0/5 | 0 (0 – NaN) | 0/5 | 0 (0 – NaN) | 3/5 | 60 (20.04 – 89.98) | 3/5 | 60 (20.04 – 89.98) |
| Overall Ndom Bakem HA | 16/355 | 4.51 (2.78 – 7.23) | 30/355 | 8.45 (5.97 – 11.83) | 98/353 | 27.76 (23.34 – 32.67) | 129/355 | 36.34 (31.5 – 41.47) |
| Overall Melong HD | 33/898 | 3.67 (2.62 – 5.12) | 35/898 | 3.9 (2.81 – 5.38) | 187/888 | 21.06 (18.5 – 23.87) | 231/898 | 25.72 (22.97 – 28.68) |
| **Health District (HD): Yabassi**  **Health Area (HA): Longtoka** | | | | | | | | |
| Longtoka | 3/50 | 6 (1.95 – 17.02) | 10/50 | 20 (11.11 – 33.33) | 12/49 | 24.49 (14.47 – 38.35) | 20/50 | 40 (27.46 – 54) |
| Ndop Po | 11/26 | 42.31 (25.2 – 61.49) | 10/26 | 38.46 (22.1 – 57.93) | 4/26 | 15.38 (5.9 – 34.54) | 16/26 | 61.54 (42.07 – 77.9) |
| Ngo Djamen | 4/7 | 57.14 (22.98 – 85.63) | 4/7 | 57.14 (22.98 – 85.63) | 3/7 | 42.86 (14.37 – 77.02) | 5/7 | 71.43 (32.66 – 92.8) |
| Nkongmalang | 37/66 | 56.06 (43.97 – 67.47) | 31/66 | 46.97 (35.33 – 58.95) | 23/65 | 35.38 (24.78 – 47.66) | 51/67 | 76.12 (64.51 – 84.82) |
| Overall Longtoka HA | 55/149 | 36.91 (29.55 – 44.94) | 55/149 | 36.91 (29.55 – 44.94) | 42/147 | 28.57 (21.86 – 36.39) | 92/150 | 61.33 (53.31 – 68.78) |
| **Health District (HD): Yabassi**  **Health Area (HA): Sole** | | | | | | | | |
| Bakem | 1/11 | 9.09 (1.26 – 43.86) | 8/11 | 72.73 (41.43 – 90.95) | 4/10 | 40 (15.83 – 70.26) | 9/11 | 81.82 (49.3 – 95.42) |
| Dibong/Gic Mafil | 7/74 | 9.46 (4.58 – 18.54) | 5/74 | 6.76 (2.84 – 15.23) | 15/73 | 20.55 (12.78 – 31.33) | 24/74 | 32.43 (22.78 – 43.85) |
| Ndocknack | 1/3 | 33.33 (4.34 – 84.65) | 0/3 | 0 (0 – NaN) | 0/3 | 0 (0 – NaN) | 1/3 | 33.33 (4.34 – 84.65) |
| Ndogminya | 3/20 | 15 (4.92 – 37.58) | 1/20 | 5 (0.7 – 28.22) | 4/18 | 22.22 (8.6 – 46.47) | 6/20 | 30 (14.14 – 52.72) |
| Nkokom | 14/48 | 29.17 (18.1 – 43.42) | 14/48 | 29.17 (18.1 – 43.42) | 24/48 | 50 (36.22 – 63.78) | 27/48 | 56.25 (42.09 – 69.46) |
| Sole | 11/62 | 17.74 (10.11 – 29.27) | 0/62 | 0 (0 – NaN) | 32/62 | 51.61 (39.33 – 63.71) | 34/63 | 53.97 (41.67 – 65.8) |
| Tamaco | 0/1 | 0 (0 – NaN) | 0/1 | 0 (0 – NaN) | 0/1 | 0 (0 – NaN) | 0/1 | 0 (0 – NaN) |
| Overall Sole HA | 37/219 | 16.89 (12.49 – 22.45) | 28/219 | 12.79 (8.98 – 17.9) | 79/215 | 36.74 (30.57 – 43.39) | 101/220 | 45.91 (39.43 – 52.53) |
| **Health District (HD): Yabassi**  **Health Area (HA): Yabassi** | | | | | | | | |
| Banya 1 | 6/76 | 7.89 (3.59 – 16.48) | 8/76 | 10.53 (5.35 – 19.66) | 10/74 | 13.51 (7.43 – 23.33) | 16/76 | 21.05 (13.32 – 31.64) |
| Banya 2 | 3/6 | 50 (16.79 – 83.21) | 3/6 | 50 (16.79 – 83.21) | 2/6 | 33.33 (8.39 – 73.19) | 4/6 | 66.67 (26.81 – 91.61) |
| Banya Yabassi | 0/1 | 0 (0 – NaN) | 0/1 | 0 (0 – NaN) | 0/1 | 0 (0 – NaN) | 0/1 | 0 (0 – NaN) |
| Bonabadinga | 3/22 | 13.64 (4.46 – 34.79) | 18/22 | 81.82 (60.36 – 93.01) | 10/22 | 45.45 (26.47 – 65.86) | 21/22 | 95.45 (73.85 – 99.36) |
| Bonabekeng | 7/35 | 20 (9.85 – 36.4) | 14/35 | 40 (25.32 – 56.73) | 9/35 | 25.71 (13.96 – 42.49) | 20/35 | 57.14 (40.57 – 72.25) |
| Mission Catholique | 0/3 | 0 (0 – NaN) | 0/3 | 0 (0 – NaN) | 0/2 | 0 (0 – NaN) | 0/3 | 0 (0 – NaN) |
| Ndogbele 1 | 6/37 | 16.22 (7.47 – 31.69) | 15/37 | 40.54 (26.13 – 56.79) | 7/36 | 19.44 (9.56 – 35.53) | 21/38 | 55.26 (39.46 – 70.07) |
| Ndogbele 2 | 1/5 | 20 (2.72 – 69.1) | 0/5 | 0 (0 – NaN) | 2/5 | 40 (10.02 – 79.96) | 2/5 | 40 (10.02 – 79.96) |
| Ndogkoko | 0/1 | 0 (0 – NaN) | 0/1 | 0 (0 – NaN) | 1/1 | 100 (NaN – NaN) | 1/1 | 100 (NaN – NaN) |
| Ndogndack | 12/25 | 48 (29.64 – 66.92) | 16/25 | 64 (44 – 80.09) | 3/24 | 12.5 (4.09 – 32.38) | 18/25 | 72 (51.78 – 86.03) |
| Nyamtan | 9/28 | 32.14 (17.65 – 51.15) | 10/28 | 35.71 (20.41 – 54.62) | 7/28 | 25 (12.41 – 43.95) | 14/28 | 50 (32.28 – 67.72) |
| Overall Yabassi HA | 47/239 | 19.67 (15.11 – 25.19) | 84/239 | 35.15 (29.36 – 41.41) | 51/234 | 21.79 (16.97 – 27.54) | 117/240 | 48.75 (42.48 – 55.06) |
| Overall Yabassi HD | 139/607 | 22.9 (19.73 – 26.41) | 167/607 | 27.51 (24.11 – 31.2) | 172/596 | 28.86 (25.36 – 32.63) | 310/610 | 50.82 (46.86 – 54.77) |

**Table B: Prevalence of intestinal nematodes per community**

| **Community Name** | ***A. lumbricoides*** | | ***Capillaria* spp.** | | ***E. vermicularis*** | | **Hookworms** | | ***S. stercoralis*** | | ***T. trichiura*** | | ***Trichostrongylus* spp.** | | **Intestinal nematodes** | |
| --- | --- | --- | --- | --- | --- | --- | --- | --- | --- | --- | --- | --- | --- | --- | --- | --- |
|  | **x/n** | **Prevalence (CI95)** | **x/n** | **Prevalence (CI95)** | **x/n** | **Prevalence (CI95)** | **x/n** | **Prevalence (CI95)** | **x/n** | **Prevalence (CI95)** | **x/n** | **Prevalence (CI95)** | **x/n** | **Prevalence (CI95)** | **x/n** | **Prevalence (CI95)** |
| **Health District (HD): Loum**  **Health Area (HA): Babong** | | | | | | | | | | | | | | | | |
| Badjoki | 0/26 | 0 (0 – NaN) | 1/26 | 3.85 (0.54 – 22.79) | 0/26 | 0 (0 – NaN) | 0/26 | 0 (0 – NaN) | 0/26 | 0 (0 – NaN) | 0/26 | 0 (0 – NaN) | 0/26 | 0 (0 – NaN) | 1/35 | 2.86 (0.4 – 17.69) |
| Mabombe | 3/23 | 13.04 (4.27 – 33.55) | 3/23 | 13.04 (4.27 – 33.55) | 3/23 | 13.04 (4.27 – 33.55) | 0/23 | 0 (0 – NaN) | 0/23 | 0 (0 – NaN) | 1/23 | 4.35 (0.61 – 25.22) | 0/23 | 0 (0 – NaN) | 6/35 | 17.14 (7.91 – 33.26) |
| Ngondo | 0/11 | 0 (0 – NaN) | 2/11 | 18.18 (4.58 – 50.7) | 0/11 | 0 (0 – NaN) | 0/11 | 0 (0 – NaN) | 0/11 | 0 (0 – NaN) | 0/11 | 0 (0 – NaN) | 0/11 | 0 (0 – NaN) | 2/16 | 12.5 (3.14 – 38.6) |
| Nkongbissi/Salaka | 1/30 | 3.33 (0.47 – 20.2) | 5/30 | 16.67 (7.11 – 34.32) | 0/30 | 0 (0 – NaN) | 0/30 | 0 (0 – NaN) | 0/30 | 0 (0 – NaN) | 0/30 | 0 (0 – NaN) | 0/30 | 0 (0 – NaN) | 5/37 | 13.51 (5.74 – 28.62) |
| Overall Babong HA | 4/90 | 4.44 (1.68 – 11.25) | 11/90 | 12.22 (6.9 – 20.74) | 3/90 | 3.33 (1.08 – 9.83) | 0/90 | 0 (0 – NaN) | 0/90 | 0 (0 – NaN) | 1/90 | 1.11 (0.16 – 7.46) | 0/90 | 0 (0 – NaN) | 14/123 | 11.38 (6.86 – 18.3) |
| **Health District (HD): Loum**  **Health Area (HA): Loum 2** | | | | | | | | | | | | | | | | |
| Bonabela | 0/8 | 0 (0 – NaN) | 0/8 | 0 (0 – NaN) | 0/8 | 0 (0 – NaN) | 0/8 | 0 (0 – NaN) | 0/8 | 0 (0 – NaN) | 0/8 | 0 (0 – NaN) | 0/8 | 0 (0 – NaN) | 0/9 | 0 (0 – NaN) |
| Bonkeng | 0/4 | 0 (0 – NaN) | 0/4 | 0 (0 – NaN) | 0/4 | 0 (0 – NaN) | 0/4 | 0 (0 – NaN) | 0/4 | 0 (0 – NaN) | 0/4 | 0 (0 – NaN) | 0/4 | 0 (0 – NaN) | 0/20 | 0 (0 – NaN) |
| Dimithe | 2/27 | 7.41 (1.86 – 25.25) | 2/27 | 7.41 (1.86 – 25.25) | 1/27 | 3.7 (0.52 – 22.08) | 0/27 | 0 (0 – NaN) | 0/27 | 0 (0 – NaN) | 1/27 | 3.7 (0.52 – 22.08) | 0/27 | 0 (0 – NaN) | 5/32 | 15.62 (6.66 – 32.47) |
| Overall Loum 2 HA | 2/39 | 5.13 (1.29 – 18.32) | 2/39 | 5.13 (1.29 – 18.32) | 1/39 | 2.56 (0.36 – 16.08) | 0/39 | 0 (0 – NaN) | 0/39 | 0 (0 – NaN) | 1/39 | 2.56 (0.36 – 16.08) | 0/39 | 0 (0 – NaN) | 5/61 | 8.2 (3.45 – 18.23) |
| Overall Loum HD | 6/129 | 4.65 (2.1 – 9.97) | 13/129 | 10.08 (5.94 – 16.58) | 4/129 | 3.1 (1.17 – 7.97) | 0/129 | 0 (0 – NaN) | 0/129 | 0 (0 – NaN) | 2/129 | 1.55 (0.39 – 5.98) | 0/129 | 0 (0 – NaN) | 19/184 | 10.33 (6.68 – 15.62) |
| **Health District (HD): Manjo**  **Health Area (HA): Kolla** | | | | | | | | | | | | | | | | |
| Kolla Carrefour | 0/1 | 0 (0 – NaN) | 0/1 | 0 (0 – NaN) | 0/1 | 0 (0 – NaN) | 0/1 | 0 (0 – NaN) | 0/1 | 0 (0 – NaN) | 0/1 | 0 (0 – NaN) | 0/1 | 0 (0 – NaN) | 0/1 | 0 (0 – NaN) |
| Kolla Lala | 0/6 | 0 (0 – NaN) | 0/6 | 0 (0 – NaN) | 0/6 | 0 (0 – NaN) | 0/6 | 0 (0 – NaN) | 0/6 | 0 (0 – NaN) | 1/6 | 16.67 (2.28 – 63.13) | 0/6 | 0 (0 – NaN) | 1/7 | 14.29 (1.97 – 58.06) |
| Kolla Songo |  |  |  |  |  |  |  |  |  |  |  |  |  |  | 0/5 | 0 (0 – NaN) |
| Lala Mission | 0/17 | 0 (0 – NaN) | 1/17 | 5.88 (0.82 – 32.03) | 0/17 | 0 (0 – NaN) | 0/17 | 0 (0 – NaN) | 0/17 | 0 (0 – NaN) | 2/17 | 11.76 (2.96 – 36.83) | 0/17 | 0 (0 – NaN) | 3/24 | 12.5 (4.09 – 32.38) |
| Manengoteng 1 | 0/7 | 0 (0 – NaN) | 0/7 | 0 (0 – NaN) | 0/7 | 0 (0 – NaN) | 0/7 | 0 (0 – NaN) | 0/7 | 0 (0 – NaN) | 0/7 | 0 (0 – NaN) | 0/7 | 0 (0 – NaN) | 0/9 | 0 (0 – NaN) |
| Manengoteng 2 | 3/35 | 8.57 (2.79 – 23.44) | 0/35 | 0 (0 – NaN) | 0/35 | 0 (0 – NaN) | 0/35 | 0 (0 – NaN) | 0/35 | 0 (0 – NaN) | 8/35 | 22.86 (11.86 – 39.47) | 0/35 | 0 (0 – NaN) | 9/54 | 16.67 (8.91 – 29.03) |
| Mantem 1 | 0/1 | 0 (0 – NaN) | 0/1 | 0 (0 – NaN) | 0/1 | 0 (0 – NaN) | 0/1 | 0 (0 – NaN) | 0/1 | 0 (0 – NaN) | 0/1 | 0 (0 – NaN) | 0/1 | 0 (0 – NaN) | 0/3 | 0 (0 – NaN) |
| Overall Kolla HA | 3/67 | 4.48 (1.45 – 12.98) | 1/67 | 1.49 (0.21 – 9.84) | 0/67 | 0 (0 – NaN) | 0/67 | 0 (0 – NaN) | 0/67 | 0 (0 – NaN) | 11/67 | 16.42 (9.33 – 27.27) | 0/67 | 0 (0 – NaN) | 13/103 | 12.62 (7.47 – 20.53) |
| **Health District (HD): Manjo**  **Health Area (HA): Manjo 1** | | | | | | | | | | | | | | | | |
| Boum-Quartier 8 | 1/11 | 9.09 (1.26 – 43.86) | 0/11 | 0 (0 – NaN) | 0/11 | 0 (0 – NaN) | 0/11 | 0 (0 – NaN) | 0/11 | 0 (0 – NaN) | 0/11 | 0 (0 – NaN) | 0/11 | 0 (0 – NaN) | 1/18 | 5.56 (0.78 – 30.65) |
| Emeng | 0/1 | 0 (0 – NaN) | 0/1 | 0 (0 – NaN) | 0/1 | 0 (0 – NaN) | 0/1 | 0 (0 – NaN) | 0/1 | 0 (0 – NaN) | 0/1 | 0 (0 – NaN) | 0/1 | 0 (0 – NaN) | 0/1 | 0 (0 – NaN) |
| Manjo Centre | 0/1 | 0 (0 – NaN) | 0/1 | 0 (0 – NaN) | 0/1 | 0 (0 – NaN) | 0/1 | 0 (0 – NaN) | 0/1 | 0 (0 – NaN) | 0/1 | 0 (0 – NaN) | 0/1 | 0 (0 – NaN) | 0/1 | 0 (0 – NaN) |
| Manjo-Etam | 0/1 | 0 (0 – NaN) | 0/1 | 0 (0 – NaN) | 0/1 | 0 (0 – NaN) | 0/1 | 0 (0 – NaN) | 0/1 | 0 (0 – NaN) | 0/1 | 0 (0 – NaN) | 0/1 | 0 (0 – NaN) | 0/1 | 0 (0 – NaN) |
| Manjo-Quartier 1 | 0/28 | 0 (0 – NaN) | 0/28 | 0 (0 – NaN) | 0/28 | 0 (0 – NaN) | 1/28 | 3.57 (0.5 – 21.42) | 0/28 | 0 (0 – NaN) | 1/28 | 3.57 (0.5 – 21.42) | 0/28 | 0 (0 – NaN) | 2/48 | 4.17 (1.04 – 15.19) |
| Manjo-Quartier 2 | 0/15 | 0 (0 – NaN) | 0/15 | 0 (0 – NaN) | 0/15 | 0 (0 – NaN) | 0/15 | 0 (0 – NaN) | 0/15 | 0 (0 – NaN) | 0/15 | 0 (0 – NaN) | 0/15 | 0 (0 – NaN) | 0/30 | 0 (0 – NaN) |
| Manjo-Quartier 3 | 0/6 | 0 (0 – NaN) | 0/6 | 0 (0 – NaN) | 0/6 | 0 (0 – NaN) | 0/6 | 0 (0 – NaN) | 0/6 | 0 (0 – NaN) | 0/6 | 0 (0 – NaN) | 0/6 | 0 (0 – NaN) | 0/11 | 0 (0 – NaN) |
| Manjo-Quartier 4 | 0/4 | 0 (0 – NaN) | 0/4 | 0 (0 – NaN) | 0/4 | 0 (0 – NaN) | 0/4 | 0 (0 – NaN) | 0/4 | 0 (0 – NaN) | 1/4 | 25 (3.35 – 76.22) | 0/4 | 0 (0 – NaN) | 1/8 | 12.5 (1.73 – 53.73) |
| Namba | 0/21 | 0 (0 – NaN) | 1/21 | 4.76 (0.67 – 27.14) | 0/21 | 0 (0 – NaN) | 0/21 | 0 (0 – NaN) | 0/21 | 0 (0 – NaN) | 2/21 | 9.52 (2.39 – 31.13) | 0/21 | 0 (0 – NaN) | 3/31 | 9.68 (3.15 – 26.06) |
| Overall Manjo 1 HA | 1/88 | 1.14 (0.16 – 7.62) | 1/88 | 1.14 (0.16 – 7.62) | 0/88 | 0 (0 – NaN) | 1/88 | 1.14 (0.16 – 7.62) | 0/88 | 0 (0 – NaN) | 4/88 | 4.55 (1.72 – 11.49) | 0/88 | 0 (0 – NaN) | 7/149 | 4.7 (2.26 – 9.53) |
| **Health District (HD): Manjo**  **Health Area (HA): Manjo 2** | | | | | | | | | | | | | | | | |
| Manewang | 0/10 | 0 (0 – NaN) | 0/10 | 0 (0 – NaN) | 0/10 | 0 (0 – NaN) | 0/10 | 0 (0 – NaN) | 0/10 | 0 (0 – NaN) | 0/10 | 0 (0 – NaN) | 0/10 | 0 (0 – NaN) | 0/18 | 0 (0 – NaN) |
| Manjo-Quartier 7 | 1/1 | 100 (NaN – NaN) | 0/1 | 0 (0 – NaN) | 0/1 | 0 (0 – NaN) | 0/1 | 0 (0 – NaN) | 0/1 | 0 (0 – NaN) | 0/1 | 0 (0 – NaN) | 0/1 | 0 (0 – NaN) | 1/1 | 100 (NaN – NaN) |
| Mantem 2 | 1/4 | 25 (3.35 – 76.22) | 0/4 | 0 (0 – NaN) | 0/4 | 0 (0 – NaN) | 0/4 | 0 (0 – NaN) | 0/4 | 0 (0 – NaN) | 0/4 | 0 (0 – NaN) | 0/4 | 0 (0 – NaN) | 1/9 | 11.11 (1.54 – 49.99) |
| Mikombe-Q4 | 2/14 | 14.29 (3.6 – 42.68) | 0/14 | 0 (0 – NaN) | 0/14 | 0 (0 – NaN) | 0/14 | 0 (0 – NaN) | 0/14 | 0 (0 – NaN) | 1/14 | 7.14 (1 – 37.03) | 0/14 | 0 (0 – NaN) | 3/29 | 10.34 (3.37 – 27.6) |
| Ndiang | 1/23 | 4.35 (0.61 – 25.22) | 0/23 | 0 (0 – NaN) | 0/23 | 0 (0 – NaN) | 0/23 | 0 (0 – NaN) | 0/23 | 0 (0 – NaN) | 1/23 | 4.35 (0.61 – 25.22) | 0/23 | 0 (0 – NaN) | 2/36 | 5.56 (1.39 – 19.67) |
| Ngolsi | 0/7 | 0 (0 – NaN) | 0/7 | 0 (0 – NaN) | 0/7 | 0 (0 – NaN) | 0/7 | 0 (0 – NaN) | 0/7 | 0 (0 – NaN) | 0/7 | 0 (0 – NaN) | 0/7 | 0 (0 – NaN) | 0/8 | 0 (0 – NaN) |
| Overall Manjo 2 HA | 5/59 | 8.47 (3.57 – 18.8) | 0/59 | 0 (0 – NaN) | 0/59 | 0 (0 – NaN) | 0/59 | 0 (0 – NaN) | 0/59 | 0 (0 – NaN) | 2/59 | 3.39 (0.85 – 12.57) | 0/59 | 0 (0 – NaN) | 7/101 | 6.93 (3.34 – 13.83) |
| **Health District (HD): Manjo**  **Health Area (HA): N'Lohe** | | | | | | | | | | | | | | | | |
| N'lohe | 0/14 | 0 (0 – NaN) | 1/14 | 7.14 (1 – 37.03) | 0/14 | 0 (0 – NaN) | 0/14 | 0 (0 – NaN) | 0/14 | 0 (0 – NaN) | 1/14 | 7.14 (1 – 37.03) | 0/14 | 0 (0 – NaN) | 2/23 | 8.7 (2.18 – 28.88) |
| Overall N’lohe HA | 0/14 | 0 (0 – NaN) | 1/14 | 7.14 (1 – 37.03) | 0/14 | 0 (0 – NaN) | 0/14 | 0 (0 – NaN) | 0/14 | 0 (0 – NaN) | 1/14 | 7.14 (1 – 37.03) | 0/14 | 0 (0 – NaN) | 2/23 | 8.7 (2.18 – 28.88) |
| **Health District (HD): Manjo**  **Health Area (HA): Njoumbeng** | | | | | | | | | | | | | | | | |
| Badjoungue | 1/4 | 25 (3.35 – 76.22) | 0/4 | 0 (0 – NaN) | 0/4 | 0 (0 – NaN) | 0/4 | 0 (0 – NaN) | 0/4 | 0 (0 – NaN) | 0/4 | 0 (0 – NaN) | 0/4 | 0 (0 – NaN) | 1/9 | 11.11 (1.54 – 49.99) |
| Bigmop | 0/14 | 0 (0 – NaN) | 0/14 | 0 (0 – NaN) | 0/14 | 0 (0 – NaN) | 0/14 | 0 (0 – NaN) | 0/14 | 0 (0 – NaN) | 4/14 | 28.57 (11.15 – 56.05) | 0/14 | 0 (0 – NaN) | 4/30 | 13.33 (5.1 – 30.59) |
| Mouetouk | 0/13 | 0 (0 – NaN) | 0/13 | 0 (0 – NaN) | 0/13 | 0 (0 – NaN) | 0/13 | 0 (0 – NaN) | 0/13 | 0 (0 – NaN) | 1/13 | 7.69 (1.07 – 39.06) | 0/13 | 0 (0 – NaN) | 1/15 | 6.67 (0.93 – 35.2) |
| Njoumbeng | 1/9 | 11.11 (1.54 – 49.99) | 0/9 | 0 (0 – NaN) | 0/9 | 0 (0 – NaN) | 0/9 | 0 (0 – NaN) | 0/9 | 0 (0 – NaN) | 0/9 | 0 (0 – NaN) | 0/9 | 0 (0 – NaN) | 1/17 | 5.88 (0.82 – 32.03) |
| Overall Njoumbeng HA | 2/40 | 5 (1.25 – 17.91) | 0/40 | 0 (0 – NaN) | 0/40 | 0 (0 – NaN) | 0/40 | 0 (0 – NaN) | 0/40 | 0 (0 – NaN) | 5/40 | 12.5 (5.3 – 26.72) | 0/40 | 0 (0 – NaN) | 7/71 | 9.86 (4.77 – 19.27) |
| Overall Manjo HD | 11/268 | 4.1 (2.29 – 7.26) | 3/268 | 1.12 (0.36 – 3.41) | 0/268 | 0 (0 – NaN) | 1/268 | 0.37 (0.05 – 2.6) | 0/268 | 0 (0 – NaN) | 23/268 | 8.58 (5.77 – 12.58) | 0/268 | 0 (0 – NaN) | 36/447 | 8.05 (5.86 – 10.96) |
| **Health District (HD): Melong**  **Health Area (HA): Barehock** | | | | | | | | | | | | | | | | |
| Barehock | 0/64 | 0 (0 – NaN) | 2/64 | 3.12 (0.78 – 11.65) | 1/64 | 1.56 (0.22 – 10.27) | 0/64 | 0 (0 – NaN) | 0/64 | 0 (0 – NaN) | 0/64 | 0 (0 – NaN) | 0/64 | 0 (0 – NaN) | 3/100 | 3 (0.97 – 8.89) |
| Ebouh | 0/16 | 0 (0 – NaN) | 0/16 | 0 (0 – NaN) | 0/16 | 0 (0 – NaN) | 0/16 | 0 (0 – NaN) | 0/16 | 0 (0 – NaN) | 1/16 | 6.25 (0.87 – 33.54) | 0/16 | 0 (0 – NaN) | 1/23 | 4.35 (0.61 – 25.22) |
| Esseh | 0/2 | 0 (0 – NaN) | 0/2 | 0 (0 – NaN) | 0/2 | 0 (0 – NaN) | 0/2 | 0 (0 – NaN) | 0/2 | 0 (0 – NaN) | 0/2 | 0 (0 – NaN) | 0/2 | 0 (0 – NaN) | 0/8 | 0 (0 – NaN) |
| Mbangla | 0/5 | 0 (0 – NaN) | 0/5 | 0 (0 – NaN) | 0/5 | 0 (0 – NaN) | 0/5 | 0 (0 – NaN) | 0/5 | 0 (0 – NaN) | 0/5 | 0 (0 – NaN) | 0/5 | 0 (0 – NaN) | 0/11 | 0 (0 – NaN) |
| Mbouele Kem | 0/34 | 0 (0 – NaN) | 2/34 | 5.88 (1.48 – 20.68) | 0/34 | 0 (0 – NaN) | 0/34 | 0 (0 – NaN) | 0/34 | 0 (0 – NaN) | 0/34 | 0 (0 – NaN) | 0/34 | 0 (0 – NaN) | 2/44 | 4.55 (1.14 – 16.44) |
| Ngol | 4/42 | 9.52 (3.62 – 22.78) | 0/42 | 0 (0 – NaN) | 0/42 | 0 (0 – NaN) | 0/42 | 0 (0 – NaN) | 0/42 | 0 (0 – NaN) | 1/42 | 2.38 (0.33 – 15.06) | 0/42 | 0 (0 – NaN) | 5/54 | 9.26 (3.91 – 20.39) |
| Overall Barehock HA | 4/163 | 2.45 (0.92 – 6.35) | 4/163 | 2.45 (0.92 – 6.35) | 1/163 | 0.61 (0.09 – 4.22) | 0/163 | 0 (0 – NaN) | 0/163 | 0 (0 – NaN) | 2/163 | 1.23 (0.31 – 4.77) | 0/163 | 0 (0 – NaN) | 11/240 | 4.58 (2.56 – 8.09) |
| **Health District (HD): Melong**  **Health Area (HA): Melong 2** | | | | | | | | | | | | | | | | |
| Bayon | 1/44 | 2.27 (0.32 – 14.45) | 1/44 | 2.27 (0.32 – 14.45) | 0/44 | 0 (0 – NaN) | 0/44 | 0 (0 – NaN) | 0/44 | 0 (0 – NaN) | 2/44 | 4.55 (1.14 – 16.44) | 0/44 | 0 (0 – NaN) | 3/62 | 4.84 (1.57 – 13.96) |
| Eboukou | 0/1 | 0 (0 – NaN) | 0/1 | 0 (0 – NaN) | 0/1 | 0 (0 – NaN) | 0/1 | 0 (0 – NaN) | 0/1 | 0 (0 – NaN) | 0/1 | 0 (0 – NaN) | 0/1 | 0 (0 – NaN) | 0/9 | 0 (0 – NaN) |
| Ekom-Nkam | 2/42 | 4.76 (1.19 – 17.14) | 0/42 | 0 (0 – NaN) | 0/42 | 0 (0 – NaN) | 0/42 | 0 (0 – NaN) | 0/42 | 0 (0 – NaN) | 1/42 | 2.38 (0.33 – 15.06) | 0/42 | 0 (0 – NaN) | 2/69 | 2.9 (0.73 – 10.86) |
| Longko | 0/2 | 0 (0 – NaN) | 0/2 | 0 (0 – NaN) | 0/2 | 0 (0 – NaN) | 0/2 | 0 (0 – NaN) | 0/2 | 0 (0 – NaN) | 0/2 | 0 (0 – NaN) | 0/2 | 0 (0 – NaN) | 0/2 | 0 (0 – NaN) |
| Melong 2 | 1/50 | 2 (0.28 – 12.88) | 0/50 | 0 (0 – NaN) | 0/50 | 0 (0 – NaN) | 0/50 | 0 (0 – NaN) | 0/50 | 0 (0 – NaN) | 0/50 | 0 (0 – NaN) | 0/50 | 0 (0 – NaN) | 1/68 | 1.47 (0.21 – 9.71) |
| Melong Q3 | 0/15 | 0 (0 – NaN) | 0/15 | 0 (0 – NaN) | 0/15 | 0 (0 – NaN) | 0/15 | 0 (0 – NaN) | 0/15 | 0 (0 – NaN) | 0/15 | 0 (0 – NaN) | 0/15 | 0 (0 – NaN) | 0/21 | 0 (0 – NaN) |
| Melong Q4 | 1/21 | 4.76 (0.67 – 27.14) | 2/21 | 9.52 (2.39 – 31.13) | 0/21 | 0 (0 – NaN) | 0/21 | 0 (0 – NaN) | 0/21 | 0 (0 – NaN) | 1/21 | 4.76 (0.67 – 27.14) | 0/21 | 0 (0 – NaN) | 4/32 | 12.5 (4.77 – 28.94) |
| Soundop | 0/25 | 0 (0 – NaN) | 0/25 | 0 (0 – NaN) | 0/25 | 0 (0 – NaN) | 0/25 | 0 (0 – NaN) | 0/25 | 0 (0 – NaN) | 0/25 | 0 (0 – NaN) | 0/25 | 0 (0 – NaN) | 0/40 | 0 (0 – NaN) |
| Overall Melong 2 HA | 5/200 | 2.5 (1.04 – 5.86) | 3/200 | 1.5 (0.48 – 4.55) | 0/200 | 0 (0 – NaN) | 0/200 | 0 (0 – NaN) | 0/200 | 0 (0 – NaN) | 4/200 | 2 (0.75 – 5.21) | 0/200 | 0 (0 – NaN) | 10/303 | 3.3 (1.78 – 6.02) |
| **Health District (HD): Melong**  **Health Area (HA): Ndom Bakem** | | | | | | | | | | | | | | | | |
| Mandjibo | 1/30 | 3.33 (0.47 – 20.2) | 4/30 | 13.33 (5.1 – 30.59) | 0/30 | 0 (0 – NaN) | 0/30 | 0 (0 – NaN) | 0/30 | 0 (0 – NaN) | 0/30 | 0 (0 – NaN) | 0/30 | 0 (0 – NaN) | 5/37 | 13.51 (5.74 – 28.62) |
| Mbarembeng | 2/68 | 2.94 (0.74 – 11.01) | 3/68 | 4.41 (1.43 – 12.8) | 0/68 | 0 (0 – NaN) | 0/68 | 0 (0 – NaN) | 0/68 | 0 (0 – NaN) | 2/68 | 2.94 (0.74 – 11.01) | 0/68 | 0 (0 – NaN) | 7/85 | 8.24 (3.98 – 16.28) |
| Mbie | 1/26 | 3.85 (0.54 – 22.79) | 2/26 | 7.69 (1.93 – 26.07) | 0/26 | 0 (0 – NaN) | 0/26 | 0 (0 – NaN) | 0/26 | 0 (0 – NaN) | 0/26 | 0 (0 – NaN) | 0/26 | 0 (0 – NaN) | 3/32 | 9.38 (3.06 – 25.35) |
| Mounko | 1/33 | 3.03 (0.43 – 18.61) | 1/33 | 3.03 (0.43 – 18.61) | 0/33 | 0 (0 – NaN) | 0/33 | 0 (0 – NaN) | 0/33 | 0 (0 – NaN) | 1/33 | 3.03 (0.43 – 18.61) | 0/33 | 0 (0 – NaN) | 3/41 | 7.32 (2.38 – 20.37) |
| Mpaka | 1/30 | 3.33 (0.47 – 20.2) | 1/30 | 3.33 (0.47 – 20.2) | 0/30 | 0 (0 – NaN) | 0/30 | 0 (0 – NaN) | 0/30 | 0 (0 – NaN) | 0/30 | 0 (0 – NaN) | 0/30 | 0 (0 – NaN) | 2/35 | 5.71 (1.43 – 20.16) |
| Ndom | 0/42 | 0 (0 – NaN) | 1/42 | 2.38 (0.33 – 15.06) | 0/42 | 0 (0 – NaN) | 0/42 | 0 (0 – NaN) | 0/42 | 0 (0 – NaN) | 3/42 | 7.14 (2.32 – 19.93) | 0/42 | 0 (0 – NaN) | 4/56 | 7.14 (2.71 – 17.54) |
| Ndouenbot | 0/4 | 0 (0 – NaN) | 2/4 | 50 (12.35 – 87.65) | 0/4 | 0 (0 – NaN) | 0/4 | 0 (0 – NaN) | 0/4 | 0 (0 – NaN) | 0/4 | 0 (0 – NaN) | 0/4 | 0 (0 – NaN) | 2/6 | 33.33 (8.39 – 73.19) |
| Ndouenke | 0/7 | 0 (0 – NaN) | 1/7 | 14.29 (1.97 – 58.06) | 0/7 | 0 (0 – NaN) | 0/7 | 0 (0 – NaN) | 0/7 | 0 (0 – NaN) | 1/7 | 14.29 (1.97 – 58.06) | 0/7 | 0 (0 – NaN) | 1/7 | 14.29 (1.97 – 58.06) |
| Nkonia Koniama | 0/4 | 0 (0 – NaN) | 0/4 | 0 (0 – NaN) | 0/4 | 0 (0 – NaN) | 0/4 | 0 (0 – NaN) | 0/4 | 0 (0 – NaN) | 0/4 | 0 (0 – NaN) | 0/4 | 0 (0 – NaN) | 0/4 | 0 (0 – NaN) |
| Nkoniambot | 0/9 | 0 (0 – NaN) | 2/9 | 22.22 (5.6 – 57.9) | 0/9 | 0 (0 – NaN) | 0/9 | 0 (0 – NaN) | 0/9 | 0 (0 – NaN) | 1/9 | 11.11 (1.54 – 49.99) | 0/9 | 0 (0 – NaN) | 3/10 | 30 (9.98 – 62.37) |
| Nkonianke | 0/18 | 0 (0 – NaN) | 0/18 | 0 (0 – NaN) | 0/18 | 0 (0 – NaN) | 0/18 | 0 (0 – NaN) | 0/18 | 0 (0 – NaN) | 0/18 | 0 (0 – NaN) | 0/18 | 0 (0 – NaN) | 0/37 | 0 (0 – NaN) |
| Ntangtom | 1/5 | 20 (2.72 – 69.1) | 3/5 | 60 (20.04 – 89.98) | 0/5 | 0 (0 – NaN) | 0/5 | 0 (0 – NaN) | 0/5 | 0 (0 – NaN) | 0/5 | 0 (0 – NaN) | 0/5 | 0 (0 – NaN) | 3/5 | 60 (20.04 – 89.98) |
| Overall Ndom Bakem HA | 7/276 | 2.54 (1.21 – 5.22) | 20/276 | 7.25 (4.72 – 10.96) | 0/276 | 0 (0 – NaN) | 0/276 | 0 (0 – NaN) | 0/276 | 0 (0 – NaN) | 8/276 | 2.9 (1.46 – 5.69) | 0/276 | 0 (0 – NaN) | 33/355 | 9.3 (6.68 – 12.79) |
| Overall Melong HD | 16/639 | 2.5 (1.54 – 4.05) | 27/639 | 4.23 (2.91 – 6.09) | 1/639 | 0.16 (0.02 – 1.1) | 0/639 | 0 (0 – NaN) | 0/639 | 0 (0 – NaN) | 14/639 | 2.19 (1.3 – 3.66) | 0/639 | 0 (0 – NaN) | 54/898 | 6.01 (4.63 – 7.77) |
| **Health District (HD): Yabassi**  **Health Area (HA): Longtoka** | | | | | | | | | | | | | | | | |
| Longtoka | 0/42 | 0 (0 – NaN) | 0/42 | 0 (0 – NaN) | 0/42 | 0 (0 – NaN) | 0/42 | 0 (0 – NaN) | 0/42 | 0 (0 – NaN) | 0/42 | 0 (0 – NaN) | 0/42 | 0 (0 – NaN) | 0/50 | 0 (0 – NaN) |
| Ndop Po | 0/24 | 0 (0 – NaN) | 0/24 | 0 (0 – NaN) | 0/24 | 0 (0 – NaN) | 0/24 | 0 (0 – NaN) | 0/24 | 0 (0 – NaN) | 0/24 | 0 (0 – NaN) | 0/24 | 0 (0 – NaN) | 0/26 | 0 (0 – NaN) |
| Ngo Djamen | 0/6 | 0 (0 – NaN) | 0/6 | 0 (0 – NaN) | 0/6 | 0 (0 – NaN) | 0/6 | 0 (0 – NaN) | 0/6 | 0 (0 – NaN) | 0/6 | 0 (0 – NaN) | 0/6 | 0 (0 – NaN) | 0/7 | 0 (0 – NaN) |
| Nkongmalang | 1/66 | 1.52 (0.21 – 9.98) | 0/66 | 0 (0 – NaN) | 0/66 | 0 (0 – NaN) | 0/66 | 0 (0 – NaN) | 0/66 | 0 (0 – NaN) | 0/66 | 0 (0 – NaN) | 0/66 | 0 (0 – NaN) | 1/67 | 1.49 (0.21 – 9.84) |
| Overall Logtoka HA | 1/138 | 0.72 (0.1 – 4.96) | 0/138 | 0 (0 – NaN) | 0/138 | 0 (0 – NaN) | 0/138 | 0 (0 – NaN) | 0/138 | 0 (0 – NaN) | 0/138 | 0 (0 – NaN) | 0/138 | 0 (0 – NaN) | 1/150 | 0.67 (0.09 – 4.58) |
| **Health District (HD): Yabassi**  **Health Area (HA): Sole** | | | | | | | | | | | | | | | | |
| Bakem | 1/5 | 20 (2.72 – 69.1) | 1/5 | 20 (2.72 – 69.1) | 0/5 | 0 (0 – NaN) | 0/5 | 0 (0 – NaN) | 0/5 | 0 (0 – NaN) | 0/5 | 0 (0 – NaN) | 0/5 | 0 (0 – NaN) | 2/11 | 18.18 (4.58 – 50.7) |
| Dibong/Gic Mafil | 4/70 | 5.71 (2.16 – 14.26) | 0/70 | 0 (0 – NaN) | 0/70 | 0 (0 – NaN) | 0/70 | 0 (0 – NaN) | 0/70 | 0 (0 – NaN) | 0/70 | 0 (0 – NaN) | 1/70 | 1.43 (0.2 – 9.45) | 5/74 | 6.76 (2.84 – 15.23) |
| Ndocknack | 0/2 | 0 (0 – NaN) | 0/2 | 0 (0 – NaN) | 0/2 | 0 (0 – NaN) | 0/2 | 0 (0 – NaN) | 0/2 | 0 (0 – NaN) | 0/2 | 0 (0 – NaN) | 0/2 | 0 (0 – NaN) | 0/3 | 0 (0 – NaN) |
| Ndogminya | 0/20 | 0 (0 – NaN) | 0/20 | 0 (0 – NaN) | 0/20 | 0 (0 – NaN) | 0/20 | 0 (0 – NaN) | 0/20 | 0 (0 – NaN) | 0/20 | 0 (0 – NaN) | 0/20 | 0 (0 – NaN) | 0/20 | 0 (0 – NaN) |
| Nkokom | 2/41 | 4.88 (1.22 – 17.52) | 0/41 | 0 (0 – NaN) | 0/41 | 0 (0 – NaN) | 0/41 | 0 (0 – NaN) | 1/41 | 2.44 (0.34 – 15.39) | 2/41 | 4.88 (1.22 – 17.52) | 0/41 | 0 (0 – NaN) | 5/48 | 10.42 (4.4 – 22.69) |
| Sole | 1/61 | 1.64 (0.23 – 10.73) | 0/61 | 0 (0 – NaN) | 1/61 | 1.64 (0.23 – 10.73) | 0/61 | 0 (0 – NaN) | 1/61 | 1.64 (0.23 – 10.73) | 0/61 | 0 (0 – NaN) | 1/61 | 1.64 (0.23 – 10.73) | 3/63 | 4.76 (1.54 – 13.75) |
| Tamaco | 0/1 | 0 (0 – NaN) | 0/1 | 0 (0 – NaN) | 0/1 | 0 (0 – NaN) | 0/1 | 0 (0 – NaN) | 0/1 | 0 (0 – NaN) | 0/1 | 0 (0 – NaN) | 0/1 | 0 (0 – NaN) | 0/1 | 0 (0 – NaN) |
| Overall Sole HA | 8/200 | 4 (2.01 – 7.79) | 1/200 | 0.5 (0.07 – 3.46) | 1/200 | 0.5 (0.07 – 3.46) | 0/200 | 0 (0 – NaN) | 2/200 | 1 (0.25 – 3.91) | 2/200 | 1 (0.25 – 3.91) | 2/200 | 1 (0.25 – 3.91) | 15/220 | 6.82 (4.15 – 11) |
| **Health District (HD): Yabassi**  **Health Area (HA): Yabassi** | | | | | | | | | | | | | | | | |
| Banya 1 | 2/64 | 3.12 (0.78 – 11.65) | 11/64 | 17.19 (9.78 – 28.43) | 0/64 | 0 (0 – NaN) | 0/64 | 0 (0 – NaN) | 0/64 | 0 (0 – NaN) | 0/64 | 0 (0 – NaN) | 0/64 | 0 (0 – NaN) | 12/76 | 15.79 (9.19 – 25.78) |
| Banya 2 | 0/6 | 0 (0 – NaN) | 0/6 | 0 (0 – NaN) | 0/6 | 0 (0 – NaN) | 0/6 | 0 (0 – NaN) | 0/6 | 0 (0 – NaN) | 0/6 | 0 (0 – NaN) | 0/6 | 0 (0 – NaN) | 0/6 | 0 (0 – NaN) |
| Banya Yabassi | 0/1 | 0 (0 – NaN) | 0/1 | 0 (0 – NaN) | 0/1 | 0 (0 – NaN) | 0/1 | 0 (0 – NaN) | 0/1 | 0 (0 – NaN) | 0/1 | 0 (0 – NaN) | 0/1 | 0 (0 – NaN) | 0/1 | 0 (0 – NaN) |
| Bonabadinga | 0/19 | 0 (0 – NaN) | 0/19 | 0 (0 – NaN) | 0/19 | 0 (0 – NaN) | 0/19 | 0 (0 – NaN) | 0/19 | 0 (0 – NaN) | 0/19 | 0 (0 – NaN) | 0/19 | 0 (0 – NaN) | 0/22 | 0 (0 – NaN) |
| Bonabekeng | 0/27 | 0 (0 – NaN) | 10/27 | 37.04 (21.22 – 56.23) | 0/27 | 0 (0 – NaN) | 0/27 | 0 (0 – NaN) | 0/27 | 0 (0 – NaN) | 1/27 | 3.7 (0.52 – 22.08) | 0/27 | 0 (0 – NaN) | 11/35 | 31.43 (18.34 – 48.34) |
| Mission Catholique | 0/2 | 0 (0 – NaN) | 2/2 | 100 (NaN – NaN) | 0/2 | 0 (0 – NaN) | 0/2 | 0 (0 – NaN) | 0/2 | 0 (0 – NaN) | 0/2 | 0 (0 – NaN) | 0/2 | 0 (0 – NaN) | 2/3 | 66.67 (15.35 – 95.66) |
| Ndogbele 1 | 0/22 | 0 (0 – NaN) | 2/22 | 9.09 (2.28 – 29.96) | 0/22 | 0 (0 – NaN) | 0/22 | 0 (0 – NaN) | 0/22 | 0 (0 – NaN) | 0/22 | 0 (0 – NaN) | 0/22 | 0 (0 – NaN) | 2/39 | 5.13 (1.29 – 18.32) |
| Ndogbele 2 | 0/2 | 0 (0 – NaN) | 0/2 | 0 (0 – NaN) | 0/2 | 0 (0 – NaN) | 0/2 | 0 (0 – NaN) | 0/2 | 0 (0 – NaN) | 0/2 | 0 (0 – NaN) | 0/2 | 0 (0 – NaN) | 0/5 | 0 (0 – NaN) |
| Ndogkoko | 0/1 | 0 (0 – NaN) | 0/1 | 0 (0 – NaN) | 0/1 | 0 (0 – NaN) | 0/1 | 0 (0 – NaN) | 0/1 | 0 (0 – NaN) | 0/1 | 0 (0 – NaN) | 0/1 | 0 (0 – NaN) | 0/1 | 0 (0 – NaN) |
| Ndogndack | 0/16 | 0 (0 – NaN) | 1/16 | 6.25 (0.87 – 33.54) | 0/16 | 0 (0 – NaN) | 0/16 | 0 (0 – NaN) | 0/16 | 0 (0 – NaN) | 0/16 | 0 (0 – NaN) | 0/16 | 0 (0 – NaN) | 1/25 | 4 (0.56 – 23.55) |
| Nyamtan | 1/22 | 4.55 (0.64 – 26.15) | 1/22 | 4.55 (0.64 – 26.15) | 0/22 | 0 (0 – NaN) | 0/22 | 0 (0 – NaN) | 0/22 | 0 (0 – NaN) | 0/22 | 0 (0 – NaN) | 0/22 | 0 (0 – NaN) | 2/28 | 7.14 (1.79 – 24.48) |
| Overall Yabassi HA | 3/182 | 1.65 (0.53 – 4.98) | 27/182 | 14.84 (10.37 – 20.77) | 0/182 | 0 (0 – NaN) | 0/182 | 0 (0 – NaN) | 0/182 | 0 (0 – NaN) | 1/182 | 0.55 (0.08 – 3.79) | 0/182 | 0 (0 – NaN) | 30/241 | 12.45 (8.84 – 17.25) |
| Overall Yabassi HD | 12/520 | 2.31 (1.32 – 4.02) | 28/520 | 5.38 (3.74 – 7.69) | 1/520 | 0.19 (0.03 – 1.35) | 0/520 | 0 (0 – NaN) | 2/520 | 0.38 (0.1 – 1.52) | 3/520 | 0.58 (0.19 – 1.77) | 2/520 | 0.38 (0.1 – 1.52) | 46/611 | 7.53 (5.69 – 9.91) |

**Table C: Prevalence of trematodes per community**

| **Community Name** | ***Clonorchis* spp.** | | ***D. dendriticum*** | | ***Fasciola* spp.** | | ***Opisthorchis* spp.** | | ***Paragonimus* spp.** | | ***S. mansoni*** | | **Trematodes** | |
| --- | --- | --- | --- | --- | --- | --- | --- | --- | --- | --- | --- | --- | --- | --- |
|  | **x/n** | **Prevalence (CI95)** | **x/n** | **Prevalence (CI95)** | **x/n** | **Prevalence (CI95)** | **x/n** | **Prevalence (CI95)** | **x/n** | **Prevalence (CI95)** | **x/n** | **Prevalence (CI95)** | **x/n** | **Prevalence (CI95)** |
| **Health District (HD): Loum**  **Health Area (HA): Babong** | | | | | | | | | | | | | | |
| Badjoki | 0/26 | 0 (0 – NaN) | 4/26 | 15.38 (5.9 – 34.54) | 0/26 | 0 (0 – NaN) | 0/26 | 0 (0 – NaN) | 0/26 | 0 (0 – NaN) | 1/26 | 3.85 (0.54 – 22.79) | 5/26 | 19.23 (8.24 – 38.7) |
| Mabombe | 0/23 | 0 (0 – NaN) | 7/23 | 30.43 (15.25 – 51.54) | 0/23 | 0 (0 – NaN) | 0/23 | 0 (0 – NaN) | 2/23 | 8.7 (2.18 – 28.88) | 1/23 | 4.35 (0.61 – 25.22) | 10/23 | 43.48 (25.22 – 63.69) |
| Ngondo | 0/11 | 0 (0 – NaN) | 2/11 | 18.18 (4.58 – 50.7) | 0/11 | 0 (0 – NaN) | 0/11 | 0 (0 – NaN) | 1/11 | 9.09 (1.26 – 43.86) | 0/11 | 0 (0 – NaN) | 2/11 | 18.18 (4.58 – 50.7) |
| Nkongbissi/Salaka | 0/30 | 0 (0 – NaN) | 3/30 | 10 (3.26 – 26.81) | 0/30 | 0 (0 – NaN) | 0/30 | 0 (0 – NaN) | 3/30 | 10 (3.26 – 26.81) | 1/30 | 3.33 (0.47 – 20.2) | 7/30 | 23.33 (11.55 – 41.5) |
| Overall Babong HA | 0/90 | 0 (0 – NaN) | 16/90 | 17.78 (11.19 – 27.07) | 0/90 | 0 (0 – NaN) | 0/90 | 0 (0 – NaN) | 6/90 | 6.67 (3.03 – 14.05) | 3/90 | 3.33 (1.08 – 9.83) | 24/90 | 26.67 (18.56 – 36.72) |
| **Health District (HD): Loum**  **Health Area (HA): Loum 2** | | | | | | | | | | | | | | |
| Bonabela | 0/8 | 0 (0 – NaN) | 1/8 | 12.5 (1.73 – 53.73) | 0/8 | 0 (0 – NaN) | 0/8 | 0 (0 – NaN) | 0/8 | 0 (0 – NaN) | 0/8 | 0 (0 – NaN) | 1/8 | 12.5 (1.73 – 53.73) |
| Bonkeng | 0/4 | 0 (0 – NaN) | 1/4 | 25 (3.35 – 76.22) | 0/4 | 0 (0 – NaN) | 0/4 | 0 (0 – NaN) | 0/4 | 0 (0 – NaN) | 0/4 | 0 (0 – NaN) | 1/4 | 25 (3.35 – 76.22) |
| Dimithe | 0/27 | 0 (0 – NaN) | 0/27 | 0 (0 – NaN) | 0/27 | 0 (0 – NaN) | 0/27 | 0 (0 – NaN) | 0/27 | 0 (0 – NaN) | 0/27 | 0 (0 – NaN) | 0/27 | 0 (0 – NaN) |
| Overall Loum 2 HA | 0/39 | 0 (0 – NaN) | 2/39 | 5.13 (1.29 – 18.32) | 0/39 | 0 (0 – NaN) | 0/39 | 0 (0 – NaN) | 0/39 | 0 (0 – NaN) | 0/39 | 0 (0 – NaN) | 2/39 | 5.13 (1.29 – 18.32) |
| Overall Loum HD | 0/129 | 0 (0 – NaN) | 18/129 | 13.95 (8.97 – 21.06) | 0/129 | 0 (0 – NaN) | 0/129 | 0 (0 – NaN) | 6/129 | 4.65 (2.1 – 9.97) | 3/129 | 2.33 (0.75 – 6.96) | 26/129 | 20.16 (14.1 – 27.96) |
| **Health District (HD): Manjo**  **Health Area (HA): Kolla** | | | | | | | | | | | | | | |
| Kolla Carrefour | 0/1 | 0 (0 – NaN) | 0/1 | 0 (0 – NaN) | 0/1 | 0 (0 – NaN) | 0/1 | 0 (0 – NaN) | 0/1 | 0 (0 – NaN) | 0/1 | 0 (0 – NaN) | 0/1 | 0 (0 – NaN) |
| Kolla Lala | 0/6 | 0 (0 – NaN) | 0/6 | 0 (0 – NaN) | 0/6 | 0 (0 – NaN) | 0/6 | 0 (0 – NaN) | 0/6 | 0 (0 – NaN) | 0/6 | 0 (0 – NaN) | 0/6 | 0 (0 – NaN) |
| Lala Mission | 0/17 | 0 (0 – NaN) | 0/17 | 0 (0 – NaN) | 0/17 | 0 (0 – NaN) | 0/17 | 0 (0 – NaN) | 0/17 | 0 (0 – NaN) | 0/17 | 0 (0 – NaN) | 0/17 | 0 (0 – NaN) |
| Manengoteng 1 | 0/7 | 0 (0 – NaN) | 0/7 | 0 (0 – NaN) | 0/7 | 0 (0 – NaN) | 0/7 | 0 (0 – NaN) | 0/7 | 0 (0 – NaN) | 0/7 | 0 (0 – NaN) | 0/7 | 0 (0 – NaN) |
| Manengoteng 2 | 0/35 | 0 (0 – NaN) | 0/35 | 0 (0 – NaN) | 0/35 | 0 (0 – NaN) | 0/35 | 0 (0 – NaN) | 0/35 | 0 (0 – NaN) | 0/35 | 0 (0 – NaN) | 0/35 | 0 (0 – NaN) |
| Mantem 1 | 0/1 | 0 (0 – NaN) | 0/1 | 0 (0 – NaN) | 0/1 | 0 (0 – NaN) | 0/1 | 0 (0 – NaN) | 0/1 | 0 (0 – NaN) | 0/1 | 0 (0 – NaN) | 0/1 | 0 (0 – NaN) |
| Overall Kolla HA | 0/67 | 0 (0 – NaN) | 0/67 | 0 (0 – NaN) | 0/67 | 0 (0 – NaN) | 0/67 | 0 (0 – NaN) | 0/67 | 0 (0 – NaN) | 0/67 | 0 (0 – NaN) | 0/67 | 0 (0 – NaN) |
| **Health District (HD): Manjo**  **Health Area (HA): Manjo 1** | | | | | | | | | | | | | | |
| Boum-Quartier 8 | 0/11 | 0 (0 – NaN) | 0/11 | 0 (0 – NaN) | 0/11 | 0 (0 – NaN) | 0/11 | 0 (0 – NaN) | 0/11 | 0 (0 – NaN) | 2/11 | 18.18 (4.58 – 50.7) | 2/11 | 18.18 (4.58 – 50.7) |
| Emeng | 0/1 | 0 (0 – NaN) | 0/1 | 0 (0 – NaN) | 0/1 | 0 (0 – NaN) | 0/1 | 0 (0 – NaN) | 0/1 | 0 (0 – NaN) | 0/1 | 0 (0 – NaN) | 0/1 | 0 (0 – NaN) |
| Manjo Centre | 0/1 | 0 (0 – NaN) | 0/1 | 0 (0 – NaN) | 0/1 | 0 (0 – NaN) | 0/1 | 0 (0 – NaN) | 0/1 | 0 (0 – NaN) | 0/1 | 0 (0 – NaN) | 0/1 | 0 (0 – NaN) |
| Manjo-Etam | 0/1 | 0 (0 – NaN) | 0/1 | 0 (0 – NaN) | 0/1 | 0 (0 – NaN) | 0/1 | 0 (0 – NaN) | 0/1 | 0 (0 – NaN) | 0/1 | 0 (0 – NaN) | 0/1 | 0 (0 – NaN) |
| Manjo-Quartier 1 | 0/28 | 0 (0 – NaN) | 0/28 | 0 (0 – NaN) | 0/28 | 0 (0 – NaN) | 0/28 | 0 (0 – NaN) | 0/28 | 0 (0 – NaN) | 0/28 | 0 (0 – NaN) | 0/28 | 0 (0 – NaN) |
| Manjo-Quartier 2 | 0/15 | 0 (0 – NaN) | 0/15 | 0 (0 – NaN) | 0/15 | 0 (0 – NaN) | 0/15 | 0 (0 – NaN) | 0/15 | 0 (0 – NaN) | 3/15 | 20 (6.59 – 46.98) | 3/15 | 20 (6.59 – 46.98) |
| Manjo-Quartier 3 | 0/6 | 0 (0 – NaN) | 0/6 | 0 (0 – NaN) | 0/6 | 0 (0 – NaN) | 0/6 | 0 (0 – NaN) | 0/6 | 0 (0 – NaN) | 0/6 | 0 (0 – NaN) | 0/6 | 0 (0 – NaN) |
| Manjo-Quartier 4 | 0/4 | 0 (0 – NaN) | 0/4 | 0 (0 – NaN) | 0/4 | 0 (0 – NaN) | 0/4 | 0 (0 – NaN) | 0/4 | 0 (0 – NaN) | 1/4 | 25 (3.35 – 76.22) | 1/4 | 25 (3.35 – 76.22) |
| Namba | 0/21 | 0 (0 – NaN) | 0/21 | 0 (0 – NaN) | 0/21 | 0 (0 – NaN) | 0/21 | 0 (0 – NaN) | 0/21 | 0 (0 – NaN) | 0/21 | 0 (0 – NaN) | 0/21 | 0 (0 – NaN) |
| Overall Manjo 1 HA | 0/88 | 0 (0 – NaN) | 0/88 | 0 (0 – NaN) | 0/88 | 0 (0 – NaN) | 0/88 | 0 (0 – NaN) | 0/88 | 0 (0 – NaN) | 6/88 | 6.82 (3.1 – 14.36) | 6/88 | 6.82 (3.1 – 14.36) |
| **Health District (HD): Manjo**  **Health Area (HA): Manjo 2** | | | | | | | | | | | | | | |
| Manewang | 0/10 | 0 (0 – NaN) | 0/10 | 0 (0 – NaN) | 0/10 | 0 (0 – NaN) | 0/10 | 0 (0 – NaN) | 0/10 | 0 (0 – NaN) | 0/10 | 0 (0 – NaN) | 0/10 | 0 (0 – NaN) |
| Manjo-Quartier 7 | 0/1 | 0 (0 – NaN) | 0/1 | 0 (0 – NaN) | 0/1 | 0 (0 – NaN) | 0/1 | 0 (0 – NaN) | 0/1 | 0 (0 – NaN) | 0/1 | 0 (0 – NaN) | 0/1 | 0 (0 – NaN) |
| Mantem 2 | 0/4 | 0 (0 – NaN) | 0/4 | 0 (0 – NaN) | 0/4 | 0 (0 – NaN) | 0/4 | 0 (0 – NaN) | 0/4 | 0 (0 – NaN) | 0/4 | 0 (0 – NaN) | 0/4 | 0 (0 – NaN) |
| Mikombe-Q4 | 0/14 | 0 (0 – NaN) | 0/14 | 0 (0 – NaN) | 0/14 | 0 (0 – NaN) | 0/14 | 0 (0 – NaN) | 0/14 | 0 (0 – NaN) | 1/14 | 7.14 (1 – 37.03) | 1/14 | 7.14 (1 – 37.03) |
| Ndiang | 0/23 | 0 (0 – NaN) | 0/23 | 0 (0 – NaN) | 0/23 | 0 (0 – NaN) | 0/23 | 0 (0 – NaN) | 1/23 | 4.35 (0.61 – 25.22) | 0/23 | 0 (0 – NaN) | 1/23 | 4.35 (0.61 – 25.22) |
| Ngolsi | 0/7 | 0 (0 – NaN) | 0/7 | 0 (0 – NaN) | 0/7 | 0 (0 – NaN) | 0/7 | 0 (0 – NaN) | 0/7 | 0 (0 – NaN) | 0/7 | 0 (0 – NaN) | 0/7 | 0 (0 – NaN) |
| Overall Manjo 2 HA | 0/59 | 0 (0 – NaN) | 0/59 | 0 (0 – NaN) | 0/59 | 0 (0 – NaN) | 0/59 | 0 (0 – NaN) | 1/59 | 1.69 (0.24 – 11.07) | 1/59 | 1.69 (0.24 – 11.07) | 2/59 | 3.39 (0.85 – 12.57) |
| **Health District (HD): Manjo**  **Health Area (HA): N'Lohe** | | | | | | | | | | | | | | |
| N'lohe | 0/14 | 0 (0 – NaN) | 1/14 | 7.14 (1 – 37.03) | 1/14 | 7.14 (1 – 37.03) | 0/14 | 0 (0 – NaN) | 2/14 | 14.29 (3.6 – 42.68) | 0/14 | 0 (0 – NaN) | 3/14 | 21.43 (7.07 – 49.43) |
| Overall N’lohe HA | 0/14 | 0 (0 – NaN) | 1/14 | 7.14 (1 – 37.03) | 1/14 | 7.14 (1 – 37.03) | 0/14 | 0 (0 – NaN) | 2/14 | 14.29 (3.6 – 42.68) | 0/14 | 0 (0 – NaN) | 3/14 | 21.43 (7.07 – 49.43) |
| **Health District (HD): Manjo**  **Health Area (HA): Njoumbeng** | | | | | | | | | | | | | | |
| Badjoungue | 0/4 | 0 (0 – NaN) | 0/4 | 0 (0 – NaN) | 0/4 | 0 (0 – NaN) | 0/4 | 0 (0 – NaN) | 0/4 | 0 (0 – NaN) | 0/4 | 0 (0 – NaN) | 0/4 | 0 (0 – NaN) |
| Bigmop | 0/14 | 0 (0 – NaN) | 0/14 | 0 (0 – NaN) | 0/14 | 0 (0 – NaN) | 0/14 | 0 (0 – NaN) | 0/14 | 0 (0 – NaN) | 0/14 | 0 (0 – NaN) | 0/14 | 0 (0 – NaN) |
| Mouetouk | 0/13 | 0 (0 – NaN) | 0/13 | 0 (0 – NaN) | 0/13 | 0 (0 – NaN) | 0/13 | 0 (0 – NaN) | 0/13 | 0 (0 – NaN) | 0/13 | 0 (0 – NaN) | 0/13 | 0 (0 – NaN) |
| Njoumbeng | 0/9 | 0 (0 – NaN) | 0/9 | 0 (0 – NaN) | 0/9 | 0 (0 – NaN) | 0/9 | 0 (0 – NaN) | 0/9 | 0 (0 – NaN) | 0/9 | 0 (0 – NaN) | 0/9 | 0 (0 – NaN) |
| Overall Njoumbeng HA | 0/40 | 0 (0 – NaN) | 0/40 | 0 (0 – NaN) | 0/40 | 0 (0 – NaN) | 0/40 | 0 (0 – NaN) | 0/40 | 0 (0 – NaN) | 0/40 | 0 (0 – NaN) | 0/40 | 0 (0 – NaN) |
| Overall Manjo HD | 0/268 | 0 (0 – NaN) | 1/268 | 0.37 (0.05 – 2.6) | 1/268 | 0.37 (0.05 – 2.6) | 0/268 | 0 (0 – NaN) | 3/268 | 1.12 (0.36 – 3.41) | 7/268 | 2.61 (1.25 – 5.38) | 11/268 | 4.1 (2.29 – 7.26) |
| **Health District (HD): Melong**  **Health Area (HA): Barehock** | | | | | | | | | | | | | | |
| Barehock | 0/64 | 0 (0 – NaN) | 3/64 | 4.69 (1.52 – 13.55) | 0/64 | 0 (0 – NaN) | 0/64 | 0 (0 – NaN) | 3/64 | 4.69 (1.52 – 13.55) | 0/64 | 0 (0 – NaN) | 5/64 | 7.81 (3.29 – 17.43) |
| Ebouh | 0/16 | 0 (0 – NaN) | 2/16 | 12.5 (3.14 – 38.6) | 0/16 | 0 (0 – NaN) | 0/16 | 0 (0 – NaN) | 2/16 | 12.5 (3.14 – 38.6) | 0/16 | 0 (0 – NaN) | 2/16 | 12.5 (3.14 – 38.6) |
| Esseh | 0/2 | 0 (0 – NaN) | 1/2 | 50 (5.89 – 94.11) | 0/2 | 0 (0 – NaN) | 0/2 | 0 (0 – NaN) | 0/2 | 0 (0 – NaN) | 0/2 | 0 (0 – NaN) | 1/2 | 50 (5.89 – 94.11) |
| Mbangla | 0/5 | 0 (0 – NaN) | 0/5 | 0 (0 – NaN) | 0/5 | 0 (0 – NaN) | 0/5 | 0 (0 – NaN) | 0/5 | 0 (0 – NaN) | 0/5 | 0 (0 – NaN) | 0/5 | 0 (0 – NaN) |
| Mbouele Kem | 0/34 | 0 (0 – NaN) | 4/34 | 11.76 (4.49 – 27.46) | 0/34 | 0 (0 – NaN) | 0/34 | 0 (0 – NaN) | 1/34 | 2.94 (0.41 – 18.14) | 0/34 | 0 (0 – NaN) | 4/34 | 11.76 (4.49 – 27.46) |
| Ngol | 0/42 | 0 (0 – NaN) | 0/42 | 0 (0 – NaN) | 0/42 | 0 (0 – NaN) | 0/42 | 0 (0 – NaN) | 0/42 | 0 (0 – NaN) | 0/42 | 0 (0 – NaN) | 0/42 | 0 (0 – NaN) |
| Overall Barehock HA | 0/163 | 0 (0 – NaN) | 10/163 | 6.13 (3.33 – 11.03) | 0/163 | 0 (0 – NaN) | 0/163 | 0 (0 – NaN) | 6/163 | 3.68 (1.66 – 7.95) | 0/163 | 0 (0 – NaN) | 12/163 | 7.36 (4.23 – 12.52) |
| **Health District (HD): Melong**  **Health Area (HA): Melong 2** | | | | | | | | | | | | | | |
| Bayon | 0/44 | 0 (0 – NaN) | 5/44 | 11.36 (4.81 – 24.54) | 0/44 | 0 (0 – NaN) | 0/44 | 0 (0 – NaN) | 2/44 | 4.55 (1.14 – 16.44) | 0/44 | 0 (0 – NaN) | 5/44 | 11.36 (4.81 – 24.54) |
| Eboukou | 0/1 | 0 (0 – NaN) | 0/1 | 0 (0 – NaN) | 0/1 | 0 (0 – NaN) | 0/1 | 0 (0 – NaN) | 0/1 | 0 (0 – NaN) | 0/1 | 0 (0 – NaN) | 0/1 | 0 (0 – NaN) |
| Ekom-Nkam | 0/42 | 0 (0 – NaN) | 2/42 | 4.76 (1.19 – 17.14) | 0/42 | 0 (0 – NaN) | 1/42 | 2.38 (0.33 – 15.06) | 0/42 | 0 (0 – NaN) | 0/42 | 0 (0 – NaN) | 3/42 | 7.14 (2.32 – 19.93) |
| Longko | 0/2 | 0 (0 – NaN) | 0/2 | 0 (0 – NaN) | 0/2 | 0 (0 – NaN) | 0/2 | 0 (0 – NaN) | 0/2 | 0 (0 – NaN) | 0/2 | 0 (0 – NaN) | 0/2 | 0 (0 – NaN) |
| Melong 2 | 0/50 | 0 (0 – NaN) | 3/50 | 6 (1.95 – 17.02) | 0/50 | 0 (0 – NaN) | 0/50 | 0 (0 – NaN) | 1/50 | 2 (0.28 – 12.88) | 3/50 | 6 (1.95 – 17.02) | 6/50 | 12 (5.49 – 24.24) |
| Melong Q3 | 0/15 | 0 (0 – NaN) | 1/15 | 6.67 (0.93 – 35.2) | 0/15 | 0 (0 – NaN) | 0/15 | 0 (0 – NaN) | 0/15 | 0 (0 – NaN) | 0/15 | 0 (0 – NaN) | 1/15 | 6.67 (0.93 – 35.2) |
| Melong Q4 | 0/21 | 0 (0 – NaN) | 0/21 | 0 (0 – NaN) | 0/21 | 0 (0 – NaN) | 0/21 | 0 (0 – NaN) | 2/21 | 9.52 (2.39 – 31.13) | 0/21 | 0 (0 – NaN) | 2/21 | 9.52 (2.39 – 31.13) |
| Soundop | 0/25 | 0 (0 – NaN) | 0/25 | 0 (0 – NaN) | 0/25 | 0 (0 – NaN) | 0/25 | 0 (0 – NaN) | 0/25 | 0 (0 – NaN) | 0/25 | 0 (0 – NaN) | 0/25 | 0 (0 – NaN) |
| Overall Melong 2 HA | 0/200 | 0 (0 – NaN) | 11/200 | 5.5 (3.07 – 9.66) | 0/200 | 0 (0 – NaN) | 1/200 | 0.5 (0.07 – 3.46) | 5/200 | 2.5 (1.04 – 5.86) | 3/200 | 1.5 (0.48 – 4.55) | 17/200 | 8.5 (5.35 – 13.25) |
| **Health District (HD): Melong**  **Health Area (HA): Ndom Bakem** | | | | | | | | | | | | | | |
| Mandjibo | 0/30 | 0 (0 – NaN) | 9/30 | 30 (16.41 – 48.34) | 0/30 | 0 (0 – NaN) | 0/30 | 0 (0 – NaN) | 2/30 | 6.67 (1.67 – 23.07) | 0/30 | 0 (0 – NaN) | 10/30 | 33.33 (18.97 – 51.65) |
| Mbarembeng | 0/68 | 0 (0 – NaN) | 8/68 | 11.76 (5.99 – 21.8) | 1/68 | 1.47 (0.21 – 9.71) | 0/68 | 0 (0 – NaN) | 7/68 | 10.29 (4.99 – 20.06) | 0/68 | 0 (0 – NaN) | 11/68 | 16.18 (9.19 – 26.9) |
| Mbie | 0/26 | 0 (0 – NaN) | 7/26 | 26.92 (13.41 – 46.71) | 0/26 | 0 (0 – NaN) | 0/26 | 0 (0 – NaN) | 2/26 | 7.69 (1.93 – 26.07) | 0/26 | 0 (0 – NaN) | 8/26 | 30.77 (16.2 – 50.55) |
| Mounko | 0/33 | 0 (0 – NaN) | 6/33 | 18.18 (8.4 – 34.99) | 1/33 | 3.03 (0.43 – 18.61) | 0/33 | 0 (0 – NaN) | 1/33 | 3.03 (0.43 – 18.61) | 0/33 | 0 (0 – NaN) | 6/33 | 18.18 (8.4 – 34.99) |
| Mpaka | 0/30 | 0 (0 – NaN) | 2/30 | 6.67 (1.67 – 23.07) | 0/30 | 0 (0 – NaN) | 0/30 | 0 (0 – NaN) | 1/30 | 3.33 (0.47 – 20.2) | 0/30 | 0 (0 – NaN) | 3/30 | 10 (3.26 – 26.81) |
| Ndom | 0/42 | 0 (0 – NaN) | 3/42 | 7.14 (2.32 – 19.93) | 0/42 | 0 (0 – NaN) | 0/42 | 0 (0 – NaN) | 0/42 | 0 (0 – NaN) | 1/42 | 2.38 (0.33 – 15.06) | 4/42 | 9.52 (3.62 – 22.78) |
| Ndouenbot | 0/4 | 0 (0 – NaN) | 3/4 | 75 (23.78 – 96.65) | 0/4 | 0 (0 – NaN) | 0/4 | 0 (0 – NaN) | 1/4 | 25 (3.35 – 76.22) | 0/4 | 0 (0 – NaN) | 3/4 | 75 (23.78 – 96.65) |
| Ndouenke | 0/7 | 0 (0 – NaN) | 2/7 | 28.57 (7.2 – 67.34) | 0/7 | 0 (0 – NaN) | 0/7 | 0 (0 – NaN) | 2/7 | 28.57 (7.2 – 67.34) | 0/7 | 0 (0 – NaN) | 3/7 | 42.86 (14.37 – 77.02) |
| Nkonia Koniama | 0/4 | 0 (0 – NaN) | 0/4 | 0 (0 – NaN) | 0/4 | 0 (0 – NaN) | 0/4 | 0 (0 – NaN) | 0/4 | 0 (0 – NaN) | 0/4 | 0 (0 – NaN) | 0/4 | 0 (0 – NaN) |
| Nkoniambot | 0/9 | 0 (0 – NaN) | 1/9 | 11.11 (1.54 – 49.99) | 0/9 | 0 (0 – NaN) | 0/9 | 0 (0 – NaN) | 1/9 | 11.11 (1.54 – 49.99) | 0/9 | 0 (0 – NaN) | 2/9 | 22.22 (5.6 – 57.9) |
| Nkonianke | 0/18 | 0 (0 – NaN) | 0/18 | 0 (0 – NaN) | 0/18 | 0 (0 – NaN) | 0/18 | 0 (0 – NaN) | 1/18 | 5.56 (0.78 – 30.65) | 0/18 | 0 (0 – NaN) | 1/18 | 5.56 (0.78 – 30.65) |
| Ntangtom | 0/5 | 0 (0 – NaN) | 4/5 | 80 (30.9 – 97.28) | 0/5 | 0 (0 – NaN) | 0/5 | 0 (0 – NaN) | 1/5 | 20 (2.72 – 69.1) | 0/5 | 0 (0 – NaN) | 4/5 | 80 (30.9 – 97.28) |
| Overall Ndom Bakem HA | 0/276 | 0 (0 – NaN) | 45/276 | 16.3 (12.4 – 21.14) | 2/276 | 0.72 (0.18 – 2.85) | 0/276 | 0 (0 – NaN) | 19/276 | 6.88 (4.43 – 10.54) | 1/276 | 0.36 (0.05 – 2.53) | 55/276 | 19.93 (15.63 – 25.06) |
| Overall Melong HD | 0/639 | 0 (0 – NaN) | 66/639 | 10.33 (8.2 – 12.94) | 2/639 | 0.31 (0.08 – 1.24) | 1/639 | 0.16 (0.02 – 1.1) | 30/639 | 4.69 (3.3 – 6.64) | 4/639 | 0.63 (0.24 – 1.66) | 84/639 | 13.15 (10.74 – 15.99) |
| **Health District (HD): Yabassi**  **Health Area (HA): Longtoka** | | | | | | | | | | | | | | |
| Longtoka | 0/42 | 0 (0 – NaN) | 0/42 | 0 (0 – NaN) | 0/42 | 0 (0 – NaN) | 0/42 | 0 (0 – NaN) | 1/42 | 2.38 (0.33 – 15.06) | 0/42 | 0 (0 – NaN) | 1/42 | 2.38 (0.33 – 15.06) |
| Ndop Po | 0/24 | 0 (0 – NaN) | 0/24 | 0 (0 – NaN) | 0/24 | 0 (0 – NaN) | 0/24 | 0 (0 – NaN) | 0/24 | 0 (0 – NaN) | 0/24 | 0 (0 – NaN) | 0/24 | 0 (0 – NaN) |
| Ngo Djamen | 0/6 | 0 (0 – NaN) | 0/6 | 0 (0 – NaN) | 0/6 | 0 (0 – NaN) | 0/6 | 0 (0 – NaN) | 0/6 | 0 (0 – NaN) | 0/6 | 0 (0 – NaN) | 0/6 | 0 (0 – NaN) |
| Nkongmalang | 0/66 | 0 (0 – NaN) | 0/66 | 0 (0 – NaN) | 0/66 | 0 (0 – NaN) | 0/66 | 0 (0 – NaN) | 2/66 | 3.03 (0.76 – 11.32) | 0/66 | 0 (0 – NaN) | 2/66 | 3.03 (0.76 – 11.32) |
| Overall Longtoka HA | 0/138 | 0 (0 – NaN) | 0/138 | 0 (0 – NaN) | 0/138 | 0 (0 – NaN) | 0/138 | 0 (0 – NaN) | 3/138 | 2.17 (0.7 – 6.52) | 0/138 | 0 (0 – NaN) | 3/138 | 2.17 (0.7 – 6.52) |
| **Health District (HD): Yabassi**  **Health Area (HA): Sole** | | | | | | | | | | | | | | |
| Bakem | 0/5 | 0 (0 – NaN) | 1/5 | 20 (2.72 – 69.1) | 0/5 | 0 (0 – NaN) | 0/5 | 0 (0 – NaN) | 1/5 | 20 (2.72 – 69.1) | 1/5 | 20 (2.72 – 69.1) | 2/5 | 40 (10.02 – 79.96) |
| Dibong/Gic Mafil | 0/70 | 0 (0 – NaN) | 1/70 | 1.43 (0.2 – 9.45) | 0/70 | 0 (0 – NaN) | 0/70 | 0 (0 – NaN) | 0/70 | 0 (0 – NaN) | 1/70 | 1.43 (0.2 – 9.45) | 2/70 | 2.86 (0.72 – 10.71) |
| Ndocknack | 0/2 | 0 (0 – NaN) | 0/2 | 0 (0 – NaN) | 0/2 | 0 (0 – NaN) | 0/2 | 0 (0 – NaN) | 1/2 | 50 (5.89 – 94.11) | 0/2 | 0 (0 – NaN) | 1/2 | 50 (5.89 – 94.11) |
| Ndogminya | 0/20 | 0 (0 – NaN) | 0/20 | 0 (0 – NaN) | 0/20 | 0 (0 – NaN) | 0/20 | 0 (0 – NaN) | 0/20 | 0 (0 – NaN) | 0/20 | 0 (0 – NaN) | 0/20 | 0 (0 – NaN) |
| Nkokom | 0/41 | 0 (0 – NaN) | 0/41 | 0 (0 – NaN) | 0/41 | 0 (0 – NaN) | 0/41 | 0 (0 – NaN) | 1/41 | 2.44 (0.34 – 15.39) | 0/41 | 0 (0 – NaN) | 1/41 | 2.44 (0.34 – 15.39) |
| Sole | 0/61 | 0 (0 – NaN) | 0/61 | 0 (0 – NaN) | 0/61 | 0 (0 – NaN) | 0/61 | 0 (0 – NaN) | 1/61 | 1.64 (0.23 – 10.73) | 0/61 | 0 (0 – NaN) | 1/61 | 1.64 (0.23 – 10.73) |
| Tamaco | 0/1 | 0 (0 – NaN) | 0/1 | 0 (0 – NaN) | 0/1 | 0 (0 – NaN) | 0/1 | 0 (0 – NaN) | 0/1 | 0 (0 – NaN) | 0/1 | 0 (0 – NaN) | 0/1 | 0 (0 – NaN) |
| Overall Sole HA | 0/200 | 0 (0 – NaN) | 2/200 | 1 (0.25 – 3.91) | 0/200 | 0 (0 – NaN) | 0/200 | 0 (0 – NaN) | 4/200 | 2 (0.75 – 5.21) | 2/200 | 1 (0.25 – 3.91) | 7/200 | 3.5 (1.68 – 7.16) |
| **Health District (HD): Yabassi**  **Health Area (HA): Yabassi** | | | | | | | | | | | | | | |
| Banya 1 | 1/64 | 1.56 (0.22 – 10.27) | 11/64 | 17.19 (9.78 – 28.43) | 0/64 | 0 (0 – NaN) | 0/64 | 0 (0 – NaN) | 3/64 | 4.69 (1.52 – 13.55) | 1/64 | 1.56 (0.22 – 10.27) | 14/64 | 21.88 (13.41 – 33.62) |
| Banya 2 | 0/6 | 0 (0 – NaN) | 0/6 | 0 (0 – NaN) | 0/6 | 0 (0 – NaN) | 0/6 | 0 (0 – NaN) | 0/6 | 0 (0 – NaN) | 0/6 | 0 (0 – NaN) | 0/6 | 0 (0 – NaN) |
| Banya Yabassi | 0/1 | 0 (0 – NaN) | 0/1 | 0 (0 – NaN) | 0/1 | 0 (0 – NaN) | 0/1 | 0 (0 – NaN) | 0/1 | 0 (0 – NaN) | 0/1 | 0 (0 – NaN) | 0/1 | 0 (0 – NaN) |
| Bonabadinga | 0/19 | 0 (0 – NaN) | 0/19 | 0 (0 – NaN) | 0/19 | 0 (0 – NaN) | 0/19 | 0 (0 – NaN) | 0/19 | 0 (0 – NaN) | 0/19 | 0 (0 – NaN) | 0/19 | 0 (0 – NaN) |
| Bonabekeng | 0/27 | 0 (0 – NaN) | 3/27 | 11.11 (3.63 – 29.33) | 0/27 | 0 (0 – NaN) | 0/27 | 0 (0 – NaN) | 3/27 | 11.11 (3.63 – 29.33) | 0/27 | 0 (0 – NaN) | 4/27 | 14.81 (5.67 – 33.46) |
| Mission Catholique | 0/2 | 0 (0 – NaN) | 0/2 | 0 (0 – NaN) | 0/2 | 0 (0 – NaN) | 0/2 | 0 (0 – NaN) | 0/2 | 0 (0 – NaN) | 0/2 | 0 (0 – NaN) | 0/2 | 0 (0 – NaN) |
| Ndogbele 1 | 0/22 | 0 (0 – NaN) | 3/22 | 13.64 (4.46 – 34.79) | 0/22 | 0 (0 – NaN) | 0/22 | 0 (0 – NaN) | 1/22 | 4.55 (0.64 – 26.15) | 0/22 | 0 (0 – NaN) | 4/22 | 18.18 (6.99 – 39.64) |
| Ndogbele 2 | 0/2 | 0 (0 – NaN) | 0/2 | 0 (0 – NaN) | 0/2 | 0 (0 – NaN) | 0/2 | 0 (0 – NaN) | 0/2 | 0 (0 – NaN) | 0/2 | 0 (0 – NaN) | 0/2 | 0 (0 – NaN) |
| Ndogkoko | 0/1 | 0 (0 – NaN) | 0/1 | 0 (0 – NaN) | 0/1 | 0 (0 – NaN) | 0/1 | 0 (0 – NaN) | 0/1 | 0 (0 – NaN) | 0/1 | 0 (0 – NaN) | 0/1 | 0 (0 – NaN) |
| Ndogndack | 0/16 | 0 (0 – NaN) | 0/16 | 0 (0 – NaN) | 0/16 | 0 (0 – NaN) | 0/16 | 0 (0 – NaN) | 0/16 | 0 (0 – NaN) | 0/16 | 0 (0 – NaN) | 0/16 | 0 (0 – NaN) |
| Nyamtan | 0/22 | 0 (0 – NaN) | 3/22 | 13.64 (4.46 – 34.79) | 0/22 | 0 (0 – NaN) | 0/22 | 0 (0 – NaN) | 1/22 | 4.55 (0.64 – 26.15) | 1/22 | 4.55 (0.64 – 26.15) | 4/22 | 18.18 (6.99 – 39.64) |
| Overall Yabassi HA | 1/182 | 0.55 (0.08 – 3.79) | 20/182 | 10.99 (7.2 – 16.42) | 0/182 | 0 (0 – NaN) | 0/182 | 0 (0 – NaN) | 8/182 | 4.4 (2.21 – 8.54) | 2/182 | 1.1 (0.28 – 4.29) | 26/182 | 14.29 (9.91 – 20.16) |
| Overall Yabassi HD | 1/520 | 0.19 (0.03 – 1.35) | 22/520 | 4.23 (2.8 – 6.34) | 0/520 | 0 (0 – NaN) | 0/520 | 0 (0 – NaN) | 15/520 | 2.88 (1.75 – 4.73) | 4/520 | 0.77 (0.29 – 2.03) | 36/520 | 6.92 (5.03 – 9.45) |

**Table D: Prevalence of cestodes per community**

| **Community Name** | ***D. caninum*** | | ***D. latum*** | | ***H. nana*** | | ***Taenia* spp.** | | **Cestodes** | |
| --- | --- | --- | --- | --- | --- | --- | --- | --- | --- | --- |
|  | **x/n** | **Prevalence (CI95)** | **x/n** | **Prevalence (CI95)** | **x/n** | **Prevalence (CI95)** | **x/n** | **Prevalence (CI95)** | **x/n** | **Prevalence (CI95)** |
| **Health District (HD): Loum**  **Health Area (HA): Babong** | | | | | | | | | | |
| Badjoki | 0/26 | 0 (0 – NaN) | 0/26 | 0 (0 – NaN) | 0/26 | 0 (0 – NaN) | 1/26 | 3.85 (0.54 – 22.79) | 1/26 | 3.85 (0.54 – 22.79) |
| Mabombe | 0/23 | 0 (0 – NaN) | 0/23 | 0 (0 – NaN) | 0/23 | 0 (0 – NaN) | 3/23 | 13.04 (4.27 – 33.55) | 3/23 | 13.04 (4.27 – 33.55) |
| Ngondo | 0/11 | 0 (0 – NaN) | 0/11 | 0 (0 – NaN) | 0/11 | 0 (0 – NaN) | 1/11 | 9.09 (1.26 – 43.86) | 1/11 | 9.09 (1.26 – 43.86) |
| Nkongbissi/Salaka | 0/30 | 0 (0 – NaN) | 0/30 | 0 (0 – NaN) | 0/30 | 0 (0 – NaN) | 1/30 | 3.33 (0.47 – 20.2) | 1/30 | 3.33 (0.47 – 20.2) |
| Overall Babong HA | 0/90 | 0 (0 – NaN) | 0/90 | 0 (0 – NaN) | 0/90 | 0 (0 – NaN) | 6/90 | 6.67 (3.03 – 14.05) | 6/90 | 6.67 (3.03 – 14.05) |
| **Health District (HD): Loum**  **Health Area (HA): Loum 2** | | | | | | | | | | |
| Bonabela | 0/8 | 0 (0 – NaN) | 0/8 | 0 (0 – NaN) | 0/8 | 0 (0 – NaN) | 1/8 | 12.5 (1.73 – 53.73) | 1/8 | 12.5 (1.73 – 53.73) |
| Bonkeng | 0/4 | 0 (0 – NaN) | 0/4 | 0 (0 – NaN) | 0/4 | 0 (0 – NaN) | 0/4 | 0 (0 – NaN) | 0/4 | 0 (0 – NaN) |
| Dimithe | 1/27 | 3.7 (0.52 – 22.08) | 0/27 | 0 (0 – NaN) | 0/27 | 0 (0 – NaN) | 3/27 | 11.11 (3.63 – 29.33) | 4/27 | 14.81 (5.67 – 33.46) |
| Overall Loum 2 HA | 1/39 | 2.56 (0.36 – 16.08) | 0/39 | 0 (0 – NaN) | 0/39 | 0 (0 – NaN) | 4/39 | 10.26 (3.9 – 24.33) | 5/39 | 12.82 (5.44 – 27.33) |
| Overall Loum HD | 1/129 | 0.78 (0.11 – 5.29) | 0/129 | 0 (0 – NaN) | 0/129 | 0 (0 – NaN) | 10/129 | 7.75 (4.22 – 13.81) | 11/129 | 8.53 (4.78 – 14.74) |
| **Health District (HD): Manjo**  **Health Area (HA): Kolla** | | | | | | | | | | |
| Kolla Carrefour | 0/1 | 0 (0 – NaN) | 0/1 | 0 (0 – NaN) | 0/1 | 0 (0 – NaN) | 0/1 | 0 (0 – NaN) | 0/1 | 0 (0 – NaN) |
| Kolla Lala | 0/6 | 0 (0 – NaN) | 0/6 | 0 (0 – NaN) | 0/6 | 0 (0 – NaN) | 0/6 | 0 (0 – NaN) | 0/6 | 0 (0 – NaN) |
| Lala Mission | 0/17 | 0 (0 – NaN) | 0/17 | 0 (0 – NaN) | 0/17 | 0 (0 – NaN) | 2/17 | 11.76 (2.96 – 36.83) | 2/17 | 11.76 (2.96 – 36.83) |
| Manengoteng 1 | 0/7 | 0 (0 – NaN) | 0/7 | 0 (0 – NaN) | 0/7 | 0 (0 – NaN) | 0/7 | 0 (0 – NaN) | 0/7 | 0 (0 – NaN) |
| Manengoteng 2 | 0/35 | 0 (0 – NaN) | 0/35 | 0 (0 – NaN) | 0/35 | 0 (0 – NaN) | 0/35 | 0 (0 – NaN) | 0/35 | 0 (0 – NaN) |
| Mantem 1 | 0/1 | 0 (0 – NaN) | 0/1 | 0 (0 – NaN) | 0/1 | 0 (0 – NaN) | 0/1 | 0 (0 – NaN) | 0/1 | 0 (0 – NaN) |
| Overall Kolla HA | 0/67 | 0 (0 – NaN) | 0/67 | 0 (0 – NaN) | 0/67 | 0 (0 – NaN) | 2/67 | 2.99 (0.75 – 11.16) | 2/67 | 2.99 (0.75 – 11.16) |
| **Health District (HD): Manjo**  **Health Area (HA): Manjo 1** | | | | | | | | | | |
| Boum-Quartier 8 | 0/11 | 0 (0 – NaN) | 0/11 | 0 (0 – NaN) | 0/11 | 0 (0 – NaN) | 0/11 | 0 (0 – NaN) | 0/11 | 0 (0 – NaN) |
| Emeng | 0/1 | 0 (0 – NaN) | 0/1 | 0 (0 – NaN) | 0/1 | 0 (0 – NaN) | 0/1 | 0 (0 – NaN) | 0/1 | 0 (0 – NaN) |
| Manjo Centre | 0/1 | 0 (0 – NaN) | 0/1 | 0 (0 – NaN) | 0/1 | 0 (0 – NaN) | 0/1 | 0 (0 – NaN) | 0/1 | 0 (0 – NaN) |
| Manjo-Etam | 0/1 | 0 (0 – NaN) | 0/1 | 0 (0 – NaN) | 0/1 | 0 (0 – NaN) | 0/1 | 0 (0 – NaN) | 0/1 | 0 (0 – NaN) |
| Manjo-Quartier 1 | 0/28 | 0 (0 – NaN) | 0/28 | 0 (0 – NaN) | 0/28 | 0 (0 – NaN) | 0/28 | 0 (0 – NaN) | 0/28 | 0 (0 – NaN) |
| Manjo-Quartier 2 | 0/15 | 0 (0 – NaN) | 0/15 | 0 (0 – NaN) | 1/15 | 6.67 (0.93 – 35.2) | 0/15 | 0 (0 – NaN) | 1/15 | 6.67 (0.93 – 35.2) |
| Manjo-Quartier 3 | 0/6 | 0 (0 – NaN) | 0/6 | 0 (0 – NaN) | 0/6 | 0 (0 – NaN) | 0/6 | 0 (0 – NaN) | 0/6 | 0 (0 – NaN) |
| Manjo-Quartier 4 | 0/4 | 0 (0 – NaN) | 0/4 | 0 (0 – NaN) | 0/4 | 0 (0 – NaN) | 0/4 | 0 (0 – NaN) | 0/4 | 0 (0 – NaN) |
| Namba | 0/21 | 0 (0 – NaN) | 0/21 | 0 (0 – NaN) | 0/21 | 0 (0 – NaN) | 0/21 | 0 (0 – NaN) | 0/21 | 0 (0 – NaN) |
| Overall Manjo 1 HA | 0/88 | 0 (0 – NaN) | 0/88 | 0 (0 – NaN) | 1/88 | 1.14 (0.16 – 7.62) | 0/88 | 0 (0 – NaN) | 1/88 | 1.14 (0.16 – 7.62) |
| **Health District (HD): Manjo**  **Health Area (HA): Manjo 2** | | | | | | | | | | |
| Manewang | 0/10 | 0 (0 – NaN) | 0/10 | 0 (0 – NaN) | 0/10 | 0 (0 – NaN) | 0/10 | 0 (0 – NaN) | 0/10 | 0 (0 – NaN) |
| Manjo-Quartier 7 | 0/1 | 0 (0 – NaN) | 0/1 | 0 (0 – NaN) | 0/1 | 0 (0 – NaN) | 0/1 | 0 (0 – NaN) | 0/1 | 0 (0 – NaN) |
| Mantem 2 | 0/4 | 0 (0 – NaN) | 0/4 | 0 (0 – NaN) | 0/4 | 0 (0 – NaN) | 0/4 | 0 (0 – NaN) | 0/4 | 0 (0 – NaN) |
| Mikombe-Q4 | 0/14 | 0 (0 – NaN) | 0/14 | 0 (0 – NaN) | 0/14 | 0 (0 – NaN) | 0/14 | 0 (0 – NaN) | 0/14 | 0 (0 – NaN) |
| Ndiang | 0/23 | 0 (0 – NaN) | 0/23 | 0 (0 – NaN) | 0/23 | 0 (0 – NaN) | 0/23 | 0 (0 – NaN) | 0/23 | 0 (0 – NaN) |
| Ngolsi | 0/7 | 0 (0 – NaN) | 0/7 | 0 (0 – NaN) | 0/7 | 0 (0 – NaN) | 0/7 | 0 (0 – NaN) | 0/7 | 0 (0 – NaN) |
| Overall Manjo 2 HA | 0/59 | 0 (0 – NaN) | 0/59 | 0 (0 – NaN) | 0/59 | 0 (0 – NaN) | 0/59 | 0 (0 – NaN) | 0/59 | 0 (0 – NaN) |
| **Health District (HD): Manjo**  **Health Area (HA): N'Lohe** | | | | | | | | | | |
| N'lohe | 0/14 | 0 (0 – NaN) | 0/14 | 0 (0 – NaN) | 0/14 | 0 (0 – NaN) | 2/14 | 14.29 (3.6 – 42.68) | 2/14 | 14.29 (3.6 – 42.68) |
| Overall N’lohe HA | 0/14 | 0 (0 – NaN) | 0/14 | 0 (0 – NaN) | 0/14 | 0 (0 – NaN) | 2/14 | 14.29 (3.6 – 42.68) | 2/14 | 14.29 (3.6 – 42.68) |
| **Health District (HD): Manjo**  **Health Area (HA): Njoumbeng** | | | | | | | | | | |
| Badjoungue | 0/4 | 0 (0 – NaN) | 0/4 | 0 (0 – NaN) | 0/4 | 0 (0 – NaN) | 0/4 | 0 (0 – NaN) | 0/4 | 0 (0 – NaN) |
| Bigmop | 0/14 | 0 (0 – NaN) | 0/14 | 0 (0 – NaN) | 0/14 | 0 (0 – NaN) | 0/14 | 0 (0 – NaN) | 0/14 | 0 (0 – NaN) |
| Mouetouk | 0/13 | 0 (0 – NaN) | 0/13 | 0 (0 – NaN) | 0/13 | 0 (0 – NaN) | 0/13 | 0 (0 – NaN) | 0/13 | 0 (0 – NaN) |
| Njoumbeng | 0/9 | 0 (0 – NaN) | 0/9 | 0 (0 – NaN) | 0/9 | 0 (0 – NaN) | 0/9 | 0 (0 – NaN) | 0/9 | 0 (0 – NaN) |
| Overall Njoumbeng HA | 0/40 | 0 (0 – NaN) | 0/40 | 0 (0 – NaN) | 0/40 | 0 (0 – NaN) | 0/40 | 0 (0 – NaN) | 0/40 | 0 (0 – NaN) |
| Overall Manjo HD | 0/268 | 0 (0 – NaN) | 0/268 | 0 (0 – NaN) | 1/268 | 0.37 (0.05 – 2.6) | 4/268 | 1.49 (0.56 – 3.91) | 5/268 | 1.87 (0.78 – 4.4) |
| **Health District (HD): Melong**  **Health Area (HA): Barehock** | | | | | | | | | | |
| Barehock | 0/64 | 0 (0 – NaN) | 0/64 | 0 (0 – NaN) | 0/64 | 0 (0 – NaN) | 3/64 | 4.69 (1.52 – 13.55) | 3/64 | 4.69 (1.52 – 13.55) |
| Ebouh | 0/16 | 0 (0 – NaN) | 0/16 | 0 (0 – NaN) | 0/16 | 0 (0 – NaN) | 1/16 | 6.25 (0.87 – 33.54) | 1/16 | 6.25 (0.87 – 33.54) |
| Esseh | 0/2 | 0 (0 – NaN) | 0/2 | 0 (0 – NaN) | 0/2 | 0 (0 – NaN) | 0/2 | 0 (0 – NaN) | 0/2 | 0 (0 – NaN) |
| Mbangla | 0/5 | 0 (0 – NaN) | 0/5 | 0 (0 – NaN) | 0/5 | 0 (0 – NaN) | 0/5 | 0 (0 – NaN) | 0/5 | 0 (0 – NaN) |
| Mbouele Kem | 0/34 | 0 (0 – NaN) | 0/34 | 0 (0 – NaN) | 0/34 | 0 (0 – NaN) | 2/34 | 5.88 (1.48 – 20.68) | 2/34 | 5.88 (1.48 – 20.68) |
| Ngol | 0/42 | 0 (0 – NaN) | 0/42 | 0 (0 – NaN) | 0/42 | 0 (0 – NaN) | 0/42 | 0 (0 – NaN) | 0/42 | 0 (0 – NaN) |
| Overall Barehock HA | 0/163 | 0 (0 – NaN) | 0/163 | 0 (0 – NaN) | 0/163 | 0 (0 – NaN) | 6/163 | 3.68 (1.66 – 7.95) | 6/163 | 3.68 (1.66 – 7.95) |
| **Health District (HD): Melong**  **Health Area (HA): Melong 2** | | | | | | | | | | |
| Bayon | 0/44 | 0 (0 – NaN) | 0/44 | 0 (0 – NaN) | 0/44 | 0 (0 – NaN) | 3/44 | 6.82 (2.22 – 19.11) | 3/44 | 6.82 (2.22 – 19.11) |
| Eboukou | 0/1 | 0 (0 – NaN) | 0/1 | 0 (0 – NaN) | 0/1 | 0 (0 – NaN) | 0/1 | 0 (0 – NaN) | 0/1 | 0 (0 – NaN) |
| Ekom-Nkam | 0/42 | 0 (0 – NaN) | 0/42 | 0 (0 – NaN) | 0/42 | 0 (0 – NaN) | 1/42 | 2.38 (0.33 – 15.06) | 1/42 | 2.38 (0.33 – 15.06) |
| Longko | 0/2 | 0 (0 – NaN) | 0/2 | 0 (0 – NaN) | 0/2 | 0 (0 – NaN) | 0/2 | 0 (0 – NaN) | 0/2 | 0 (0 – NaN) |
| Melong 2 | 0/50 | 0 (0 – NaN) | 0/50 | 0 (0 – NaN) | 0/50 | 0 (0 – NaN) | 0/50 | 0 (0 – NaN) | 0/50 | 0 (0 – NaN) |
| Melong Q3 | 0/15 | 0 (0 – NaN) | 0/15 | 0 (0 – NaN) | 0/15 | 0 (0 – NaN) | 1/15 | 6.67 (0.93 – 35.2) | 1/15 | 6.67 (0.93 – 35.2) |
| Melong Q4 | 0/21 | 0 (0 – NaN) | 0/21 | 0 (0 – NaN) | 0/21 | 0 (0 – NaN) | 4/21 | 19.05 (7.34 – 41.15) | 4/21 | 19.05 (7.34 – 41.15) |
| Soundop | 0/25 | 0 (0 – NaN) | 0/25 | 0 (0 – NaN) | 0/25 | 0 (0 – NaN) | 0/25 | 0 (0 – NaN) | 0/25 | 0 (0 – NaN) |
| Overall Melong 2 HA | 0/200 | 0 (0 – NaN) | 0/200 | 0 (0 – NaN) | 0/200 | 0 (0 – NaN) | 9/200 | 4.5 (2.36 – 8.42) | 9/200 | 4.5 (2.36 – 8.42) |
| **Health District (HD): Melong**  **Health Area (HA): Ndom Bakem** | | | | | | | | | | |
| Mandjibo | 0/30 | 0 (0 – NaN) | 0/30 | 0 (0 – NaN) | 0/30 | 0 (0 – NaN) | 1/30 | 3.33 (0.47 – 20.2) | 1/30 | 3.33 (0.47 – 20.2) |
| Mbarembeng | 0/68 | 0 (0 – NaN) | 0/68 | 0 (0 – NaN) | 0/68 | 0 (0 – NaN) | 1/68 | 1.47 (0.21 – 9.71) | 1/68 | 1.47 (0.21 – 9.71) |
| Mbie | 0/26 | 0 (0 – NaN) | 0/26 | 0 (0 – NaN) | 0/26 | 0 (0 – NaN) | 0/26 | 0 (0 – NaN) | 0/26 | 0 (0 – NaN) |
| Mounko | 0/33 | 0 (0 – NaN) | 0/33 | 0 (0 – NaN) | 0/33 | 0 (0 – NaN) | 0/33 | 0 (0 – NaN) | 0/33 | 0 (0 – NaN) |
| Mpaka | 0/30 | 0 (0 – NaN) | 0/30 | 0 (0 – NaN) | 0/30 | 0 (0 – NaN) | 0/30 | 0 (0 – NaN) | 0/30 | 0 (0 – NaN) |
| Ndom | 0/42 | 0 (0 – NaN) | 0/42 | 0 (0 – NaN) | 0/42 | 0 (0 – NaN) | 0/42 | 0 (0 – NaN) | 0/42 | 0 (0 – NaN) |
| Ndouenbot | 0/4 | 0 (0 – NaN) | 0/4 | 0 (0 – NaN) | 0/4 | 0 (0 – NaN) | 0/4 | 0 (0 – NaN) | 0/4 | 0 (0 – NaN) |
| Ndouenke | 0/7 | 0 (0 – NaN) | 0/7 | 0 (0 – NaN) | 0/7 | 0 (0 – NaN) | 0/7 | 0 (0 – NaN) | 0/7 | 0 (0 – NaN) |
| Nkonia Koniama | 0/4 | 0 (0 – NaN) | 0/4 | 0 (0 – NaN) | 0/4 | 0 (0 – NaN) | 0/4 | 0 (0 – NaN) | 0/4 | 0 (0 – NaN) |
| Nkoniambot | 0/9 | 0 (0 – NaN) | 0/9 | 0 (0 – NaN) | 0/9 | 0 (0 – NaN) | 0/9 | 0 (0 – NaN) | 0/9 | 0 (0 – NaN) |
| Nkonianke | 0/18 | 0 (0 – NaN) | 0/18 | 0 (0 – NaN) | 0/18 | 0 (0 – NaN) | 0/18 | 0 (0 – NaN) | 0/18 | 0 (0 – NaN) |
| Ntangtom | 0/5 | 0 (0 – NaN) | 0/5 | 0 (0 – NaN) | 0/5 | 0 (0 – NaN) | 2/5 | 40 (10.02 – 79.96) | 2/5 | 40 (10.02 – 79.96) |
| Overall Ndom Bakem HA | 0/276 | 0 (0 – NaN) | 0/276 | 0 (0 – NaN) | 0/276 | 0 (0 – NaN) | 4/276 | 1.45 (0.55 – 3.8) | 4/276 | 1.45 (0.55 – 3.8) |
| Overall Melong HD | 0/639 | 0 (0 – NaN) | 0/639 | 0 (0 – NaN) | 0/639 | 0 (0 – NaN) | 19/639 | 2.97 (1.9 – 4.61) | 19/639 | 2.97 (1.9 – 4.61) |
| **Health District (HD): Yabassi**  **Health Area (HA): Longtoka** | | | | | | | | | | |
| Longtoka | 0/42 | 0 (0 – NaN) | 0/42 | 0 (0 – NaN) | 0/42 | 0 (0 – NaN) | 2/42 | 4.76 (1.19 – 17.14) | 2/42 | 4.76 (1.19 – 17.14) |
| Ndop Po | 0/24 | 0 (0 – NaN) | 0/24 | 0 (0 – NaN) | 0/24 | 0 (0 – NaN) | 1/24 | 4.17 (0.58 – 24.35) | 1/24 | 4.17 (0.58 – 24.35) |
| Ngo Djamen | 0/6 | 0 (0 – NaN) | 0/6 | 0 (0 – NaN) | 0/6 | 0 (0 – NaN) | 0/6 | 0 (0 – NaN) | 0/6 | 0 (0 – NaN) |
| Nkongmalang | 0/66 | 0 (0 – NaN) | 0/66 | 0 (0 – NaN) | 0/66 | 0 (0 – NaN) | 0/66 | 0 (0 – NaN) | 0/66 | 0 (0 – NaN) |
| Overall Longtoka HA | 0/138 | 0 (0 – NaN) | 0/138 | 0 (0 – NaN) | 0/138 | 0 (0 – NaN) | 3/138 | 2.17 (0.7 – 6.52) | 3/138 | 2.17 (0.7 – 6.52) |
| **Health District (HD): Yabassi**  **Health Area (HA): Sole** | | | | | | | | | | |
| Bakem | 0/5 | 0 (0 – NaN) | 0/5 | 0 (0 – NaN) | 0/5 | 0 (0 – NaN) | 0/5 | 0 (0 – NaN) | 0/5 | 0 (0 – NaN) |
| Dibong/Gic Mafil | 0/70 | 0 (0 – NaN) | 1/70 | 1.43 (0.2 – 9.45) | 1/70 | 1.43 (0.2 – 9.45) | 4/70 | 5.71 (2.16 – 14.26) | 5/70 | 7.14 (3 – 16.04) |
| Ndocknack | 0/2 | 0 (0 – NaN) | 0/2 | 0 (0 – NaN) | 0/2 | 0 (0 – NaN) | 0/2 | 0 (0 – NaN) | 0/2 | 0 (0 – NaN) |
| Ndogminya | 0/20 | 0 (0 – NaN) | 0/20 | 0 (0 – NaN) | 0/20 | 0 (0 – NaN) | 0/20 | 0 (0 – NaN) | 0/20 | 0 (0 – NaN) |
| Nkokom | 1/41 | 2.44 (0.34 – 15.39) | 0/41 | 0 (0 – NaN) | 0/41 | 0 (0 – NaN) | 0/41 | 0 (0 – NaN) | 1/41 | 2.44 (0.34 – 15.39) |
| Sole | 0/61 | 0 (0 – NaN) | 0/61 | 0 (0 – NaN) | 0/61 | 0 (0 – NaN) | 0/61 | 0 (0 – NaN) | 0/61 | 0 (0 – NaN) |
| Tamaco | 0/1 | 0 (0 – NaN) | 0/1 | 0 (0 – NaN) | 0/1 | 0 (0 – NaN) | 0/1 | 0 (0 – NaN) | 0/1 | 0 (0 – NaN) |
| Overall Sole HA | 1/200 | 0.5 (0.07 – 3.46) | 1/200 | 0.5 (0.07 – 3.46) | 1/200 | 0.5 (0.07 – 3.46) | 4/200 | 2 (0.75 – 5.21) | 6/200 | 3 (1.35 – 6.52) |
| **Health District (HD): Yabassi**  **Health Area (HA): Yabassi** | | | | | | | | | | |
| Banya 1 | 0/64 | 0 (0 – NaN) | 0/64 | 0 (0 – NaN) | 0/64 | 0 (0 – NaN) | 3/64 | 4.69 (1.52 – 13.55) | 3/64 | 4.69 (1.52 – 13.55) |
| Banya 2 | 0/6 | 0 (0 – NaN) | 0/6 | 0 (0 – NaN) | 0/6 | 0 (0 – NaN) | 0/6 | 0 (0 – NaN) | 0/6 | 0 (0 – NaN) |
| Banya Yabassi | 0/1 | 0 (0 – NaN) | 0/1 | 0 (0 – NaN) | 0/1 | 0 (0 – NaN) | 0/1 | 0 (0 – NaN) | 0/1 | 0 (0 – NaN) |
| Bonabadinga | 0/19 | 0 (0 – NaN) | 0/19 | 0 (0 – NaN) | 0/19 | 0 (0 – NaN) | 2/19 | 10.53 (2.65 – 33.74) | 2/19 | 10.53 (2.65 – 33.74) |
| Bonabekeng | 0/27 | 0 (0 – NaN) | 0/27 | 0 (0 – NaN) | 0/27 | 0 (0 – NaN) | 2/27 | 7.41 (1.86 – 25.25) | 2/27 | 7.41 (1.86 – 25.25) |
| Mission Catholique | 0/2 | 0 (0 – NaN) | 0/2 | 0 (0 – NaN) | 0/2 | 0 (0 – NaN) | 0/2 | 0 (0 – NaN) | 0/2 | 0 (0 – NaN) |
| Ndogbele 1 | 0/22 | 0 (0 – NaN) | 0/22 | 0 (0 – NaN) | 0/22 | 0 (0 – NaN) | 0/22 | 0 (0 – NaN) | 0/22 | 0 (0 – NaN) |
| Ndogbele 2 | 1/2 | 50 (5.89 – 94.11) | 0/2 | 0 (0 – NaN) | 0/2 | 0 (0 – NaN) | 0/2 | 0 (0 – NaN) | 1/2 | 50 (5.89 – 94.11) |
| Ndogkoko | 0/1 | 0 (0 – NaN) | 0/1 | 0 (0 – NaN) | 0/1 | 0 (0 – NaN) | 0/1 | 0 (0 – NaN) | 0/1 | 0 (0 – NaN) |
| Ndogndack | 0/16 | 0 (0 – NaN) | 0/16 | 0 (0 – NaN) | 0/16 | 0 (0 – NaN) | 0/16 | 0 (0 – NaN) | 0/16 | 0 (0 – NaN) |
| Nyamtan | 0/22 | 0 (0 – NaN) | 0/22 | 0 (0 – NaN) | 0/22 | 0 (0 – NaN) | 1/22 | 4.55 (0.64 – 26.15) | 1/22 | 4.55 (0.64 – 26.15) |
| Overall Yabassi HA | 1/182 | 0.55 (0.08 – 3.79) | 0/182 | 0 (0 – NaN) | 0/182 | 0 (0 – NaN) | 8/182 | 4.4 (2.21 – 8.54) | 9/182 | 4.95 (2.59 – 9.23) |
| Overall Yabassi HD | 2/520 | 0.38 (0.1 – 1.52) | 1/520 | 0.19 (0.03 – 1.35) | 1/520 | 0.19 (0.03 – 1.35) | 15/520 | 2.88 (1.75 – 4.73) | 18/520 | 3.46 (2.19 – 5.43) |

NaN-Not a Numeric

**S Fig A. Prevalence (%) of helminths by health district, showing the community prevalence.** The prevalence of helminth parasitic groups was recorded per health district (HD). The fill colour of HDs follows the same colour guide of communities; blue lines represent rivers. From left to right, frames show the prevalence of filariae intestinal nematodes, trematodes, and cestodes, respectively. Filariae were the most abundant species across all four HDs, with Yabassi HD the most pervasive (50.8%). The Loum HD registered the highest prevalence for intestinal nematode (10.3%) trematode (20.2%) and cestode (8.5%) infections. The image was created using ArcGIS (ArcMap v10.5.5) software by Esri. Base layer shapefiles: National Institute of Cartography, Cameroon. Source and copyright information: https://services3.arcgis.com/dwv7XaFMgpPo7FSx/arcgis/rest/services/R%C3%A9gions/FeatureServer/0, https://data- minfof.opendata.arcgis.com/datasets/minfof::r%C3%A9gions/about
