# Supplementary material for: Epidemiology of helminth infections in adults in rural rainforest communities of the Nkam-Wouri River Basin, Littoral Region of Cameroon: Implications for NTDs elimination using preventive chemotherapies
Source: PLoS Negl Trop Dis. 2026 Oct 9;20(10):e0014780. doi: 10.1371/journal.pntd.0014780 (PMC13686460; doi:10.1371/journal.pntd.0014780)
Supplement: Supplementary file 2 [file pntd.0014780.s002.docx]

# S2 Appendix: Association between parasitic infections

**Table A: Association between parasite species**

***O. volvulus***

| **Parasite** | **Status** | **Infected/Examined** | **Prevalence** | **aOR** | **p-value** |
| --- | --- | --- | --- | --- | --- |
| *M. perstans* | Negative | 141/1334 | 10.57 (9.03 - 12.34) | – | – |
|  | Positive | 47/195 | 24.10 (18.60 - 30.62) | 2.12 (1.40 – 3.18) | **0.0003** |
| *Loa loa* | Negative | 153/1380 | 11.09 (9.53 - 12.86) | – | – |
|  | Positive | 35/149 | 23.49 (17.35 - 30.99) | 1.84 (1.15 – 2.90) | **0.009** |
| *D. dendriticum* | Negative | 169/1425 | 11.86 (10.28 - 13.65) | – | – |
|  | Positive | 19/104 | 18.27 (11.94 - 26.93) | 1.51 (0.84 – 2.60) | 0.15 |
| *Capillaria* spp. | Negative | 174/1459 | 11.93 (10.36 - 13.69) |  |  |
|  | Positive | 14/70 | 20.00 (12.17 - 31.09) |  |  |
| *Paragonimus* spp. | Negative | 175/1473 | 11.88 (10.32 -13.64) | – | – |
|  | Positive | 13/56 | 23.21 (13.91 - 36.13) | 1.71 (0.83 – 3.29) | 0.12 |
| *Taenia*. spp | Negative | 178/1481 | 12.02 (10.46 - 13.78) | – | – |
|  | Positive | 10/48 | 20.83 (11.51 - 34.74) | 1.71 (0.78 – 3.44) | 0.15 |
| *T. trichiura* | Negative | 181/1487 | 12.17 (10.60 -13.94) |  |  |
|  | Positive | 7/42 | 16.67 (8.08 - 31.27) |  |  |
| *A. lumbricoides* | Negative | 180/1485 | 12.12 (10.56 - 13.88) |  |  |
|  | Positive | 8/44 | 18.18 (9.28 - 32.55) |  |  |
| *S. mansoni* | Negative | 186/1510 | 12.32 (10.75 - 14.08) |  |  |
|  | Positive | 2/19 | 10.53 (2.54 - 34.67) |  |  |

### ***M. perstans***

| **Parasite** | **Status** | **Infected/Examined** | **Prevalence** | **aOR** | **p-value** |
| --- | --- | --- | --- | --- | --- |
| *O. volvulus* | Negative | 148/1341 | 11.04 (9.47 - 12.83) | – | – |
|  | Positive | 47/188 | 25.00 (19.31 - 31.71) | 2.15 (1.41 – 3.22) | **0.0003** |
| *Loa loa* | Negative | 121/1380 | 8.77 (7.38 - 10.38) | – | – |
|  | Positive | 74/149 | 49.66 (41.68 - 57.67) | 9.76 (6.69 – 14.27) | **<0.0001** |
| *D. dendriticum* | Negative | 180/1425 | 12.63 (11.00 - 14.46) |  |  |
|  | Positive | 15/104 | 14.42 (8.86 - 22.61) |  |  |
| *Capillaria spp.* | Negative | 181/1459 | 12.41 (10.81 - 14.20) | – | – |
|  | Positive | 14/70 | 20.00 (12.17 - 31.09) | 1.64 (0.81 – 3.09) | 0.15 |
| *Paragonimus* spp. | Negative | 181/1473 | 12.29 (10.71 - 14.07) | – | – |
|  | Positive | 14/56 | 25.00 (15.32 - 38.04) | 2.14 (1.04 – 4.20) | **0.03** |
| *Taenia* spp | Negative | 187/1481 | 12.63 (11.03 - 14.42) |  |  |
|  | Positive | 8/48 | 16.67 (8.49 - 30.12) |  |  |
| *T. trichiura* | Negative | 191/1487 | 12.84 (11.24 - 14.65) |  |  |
|  | Positive | 4/42 | 9.52 (3.57 - 23.01) |  |  |
| *A. lumbricoides* | Negative | 192/1485 | 12.93 (11.31 - 14.74) | – | – |
|  | Positive | 3/44 | 6.82 (2.18 - 19.34) | 0.43 (0.10 – 1.27) | 0.18 |
| *S. mansoni* | Negative | 192/1510 | 12.72 (11.13 - 14.49) |  |  |
|  | Positive | 3/19 | 15.79 (5.01 - 39.99) |  |  |

### ***Loa loa***

| **Parasite** | **Status** | **Infected/Examined** | **Prevalence** | **aOR** | **p-value** |
| --- | --- | --- | --- | --- | --- |
| *O. volvulus* | Negative | 114/1341 | 8.50 (7.12 - 10.12) | – | – |
|  | Positive | 35/188 | 18.62 (13.66 - 24.85) | 1.85 (1.15 – 2.92) | **0.0097** |
| *M. perstans* | Negative | 75/1334 | 5.62 (4.51 - 7.00) | – | – |
|  | Positive | 74/195 | 37.95 (31.39 - 44.98) | 9.98 (6.83 – 14.62) | **<0.0001** |
| *D. dendriticum* | Negative | 147/1425 | 10.32 (8.84 - 12.01) | – | – |
|  | Positive | 2/104 | 1.92 (0.48 - 7.42) | 0.12 (0.02 – 0.42) | **0.005** |
| *Capillaria* spp. | Negative | 142/1459 | 9.73 (8.31 - 11.37) |  |  |
|  | Positive | 7/70 | 10.00 (4.81 - 19.62) |  |  |
| *Paragonimus* spp. | Negative | 142/1473 | 9.64 (8.23 - 11.26) |  |  |
|  | Positive | 7/56 | 12.50 (6.03 - 24.12) |  |  |
| *Taenia* spp | Negative | 145/1481 | 9.79 (8.38 - 11.41) |  |  |
|  | Positive | 4/48 | 8.33 (3.13 - 20.38) |  |  |
| *T. trichiura* | Negative | 146/1487 | 9.82 (8.40 - 11.44) |  |  |
|  | Positive | 3/42 | 7.14 (2.29 - 20.17) |  |  |
| *A. lumbricoides* | Negative | 145/1485 | 9.76 (8.35 - 11.38) |  |  |
|  | Positive | 4/44 | 9.09 (3.41 - 22.06) |  |  |
| *S. mansoni* | Negative | 148/1510 | 9.80 (8.40 - 11.41) |  |  |
|  | Positive | 1/19 | 5.26 (0.70 - 30.58) |  |  |

### ***D. dendriticum***

| **Parasite** | **Status** | **Infected/Examined** | **Prevalence** | **aOR** | **p-value** |
| --- | --- | --- | --- | --- | --- |
| *O. volvulus* | Negative | 85/1341 | 6.34 (5.15 - 7.78) |  |  |
|  | Positive | 19/188 | 10.11 (6.53 - 15.32) |  |  |
| *M. perstans* | Negative | 89/1334 | 6.67 (5.45 - 8.14) |  |  |
|  | Positive | 15/195 | 7.69 (4.68 - 12.39) |  |  |
| *Loa loa* | Negative | 102/1380 | 7.39 (6.12 - 8.90) | – | – |
|  | Positive | 2/149 | 1.34 (0.33 - 5.23) | 0.14 (0.02 – 0.46) | **0.007** |
| *Capillaria* spp. | Negative | 77/1459 | 5.28 (4.24 - 6.55) | – | – |
|  | Positive | 27/70 | 38.57 (27.88 - 50.49) | 8.36 (4.64 – 14.84) | **<0.0001** |
| *Paragonimus* spp. | Negative | 82/1473 | 5.57 (4.50 - 6.86) | – | – |
|  | Positive | 22/56 | 39.29 (27.35 - 52.65) | 7.91 (4.09 – 15.01) | **<0.0001** |
| *Taenia* spp | Negative | 92/1481 | 6.21 (5.09 - 7.56) | – | – |
|  | Positive | 12/48 | 25.00 (14.69 - 39.23) | 3.34 (1.53 – 6.92) | **0.002** |
| *T. trichiura* | Negative | 102/1487 | 6.86 (5.68 - 8.26) |  |  |
|  | Positive | 2/42 | 4.76 (1.17 - 17.40) |  |  |
| *A. lumbricoides* | Negative | 97/1485 | 6.53 (5.38 - 7.91) | – | – |
|  | Positive | 7/44 | 15.91 (7.71 - 30.00) | 1.99 (0.72 – 4.79) | 0.15 |
| *S. mansoni* | Negative | 102/1510 | 6.75 (5.59 - 8.14) |  |  |
|  | Positive | 2/19 | 10.53 (2.54 - 34.67) |  |  |

### ***Capillaria* spp.**

| **Parasite** | **Status** | **Infected/Examined** | **Prevalence** | **aOR** | **p-value** |
| --- | --- | --- | --- | --- | --- |
| *O. volvulus* | Negative | 56/1341 | 4.18 (3.23 - 5.39) |  |  |
|  | Positive | 14/188 | 7.45 (4.45 - 12.20) |  |  |
| *M. perstans* | Negative | 56/1334 | 4.20 (3.24 - 5.42) | – | – |
|  | Positive | 14/195 | 7.18 (4.29 - 11.78) | 1.74 (0.88 – 3.22) | 0.09 |
| *Loa loa* | Negative | 63/1380 | 4.57 (3.58 - 5.80) |  |  |
|  | Positive | 7/149 | 4.70 (2.25 - 9.55) |  |  |
| *D. dendriticum* | Negative | 43/1425 | 3.02 (2.24 - 4.05) | – | – |
|  | Positive | 27/104 | 25.96 (18.41 - 35.27) | 8.13 (4.56 – 14.25) | **<0.0001** |
| *Paragonimus* spp. | Negative | 59/1473 | 4.01 (3.11 - 5.14) | – | – |
|  | Positive | 11/56 | 19.64 (11.16 - 32.23) | 2.34 (1.01 – 5.01) | **0.036** |
| *Taenia* spp | Negative | 60/1481 | 4.05 (3.16 - 5.18) | – | – |
|  | Positive | 10/48 | 20.83 (11.51 - 34.74) | 3.68 (1.56 – 7.98) | **0.002** |
| *T. trichiura* | Negative | 68/1487 | 4.57 (3.62 - 5.76) |  |  |
|  | Positive | 2/42 | 4.76 (1.17 - 17.40) |  |  |
| *A. lumbricoides* | Negative | 67/1485 | 4.51 (3.57 - 5.69) |  |  |
|  | Positive | 3/44 | 6.82 (2.18 - 19.34) |  |  |
| *S. mansoni* | Negative | 69/1510 | 4.57 (3.62 - 5.75) |  |  |
|  | Positive | 1/19 | 5.26 (0.70 - 30.58) |  |  |

### ***Paragonimus* spp.**

| **Parasite** | **Status** | **Infected/Examined** | **Prevalence** | **aOR** | **p-value** |
| --- | --- | --- | --- | --- | --- |
| *O. volvulus* | Negative | 43/1341 | 3.21 (2.39 - 4.30) | – | – |
|  | Positive | 13/188 | 6.91 (4.05 - 11.56) | 1.66 (0.80 – 3.23) | 0.15 |
| *M. perstans* | Negative | 42/1334 | 3.15 (2.33 - 4.23) | – | – |
|  | Positive | 14/195 | 7.18 (4.29 - 11.78) | 2.24 (1.11 – 4.28) | **0.02** |
| *Loa loa* | Negative | 49/1380 | 3.55 (2.69 - 4.67) |  |  |
|  | Positive | 7/149 | 4.70 (2.25 - 9.55) |  |  |
| *D. dendriticum* | Negative | 34/1425 | 2.39 (1.71 - 3.32) | – | – |
|  | Positive | 22/104 | 21.15 (14.32 - 30.10) | 9.03 (4.72 – 16.93) | **<0.0001** |
| *Capillaria* spp. | Negative | 45/1459 | 3.08 (2.31 - 4.11) | – | – |
|  | Positive | 11/70 | 15.71 (8.88 - 26.29) | 2.17 (0.92 – 4.76) | **0.06** |
| *Taenia* spp | Negative | 51/1481 | 3.44 (2.63 - 4.50) |  |  |
|  | Positive | 5/48 | 10.42 (4.36 - 22.88) |  |  |
| *T. trichiura* | Negative | 53/1487 | 3.56 (2.73 - 4.64) | – | – |
|  | Positive | 3/42 | 7.14 (2.29 - 20.17) | 2.64 (0.92 – 4.76) | 0.13 |
| *A. lumbricoides* | Negative | 52/1485 | 3.50 (2.68 - 4.57) |  |  |
|  | Positive | 4/44 | 9.09 (3.41 - 22.06) |  |  |
| *S. mansoni* | Negative | 55/1510 | 3.64 (2.81 - 4.72) |  |  |
|  | Positive | 1/19 | 5.26 (0.70 - 30.58) |  |  |

### ***Taenia* spp.**

| **Parasite** | **Status** | **Infected/Examined** | **Prevalence** | **aOR** | **p-value** |
| --- | --- | --- | --- | --- | --- |
| *O. volvulus* | Negative | 38/1341 | 2.83 (2.07 - 3.87) | – | – |
|  | Positive | 10/188 | 5.32 (2.88 - 9.62) | 1.65 (0.75 – 3.32) | 0.18 |
| *M. perstans* | Negative | 40/1334 | 3.00 (2.21 - 4.06) |  |  |
|  | Positive | 8/195 | 4.10 (2.06 - 8.01) |  |  |
| *Loa loa* | Negative | 44/1380 | 3.19 (2.38 - 4.26) |  |  |
|  | Positive | 4/149 | 2.68 (1.01 - 6.96) |  |  |
| *D. dendriticum* | Negative | 36/1425 | 2.53 (1.83 - 3.48) | – | – |
|  | Positive | 12/104 | 11.54 (6.65 - 19.28) | 3.22 (1.42 – 6.76) | **0.003** |
| *Capillaria* spp. | Negative | 38/1459 | 2.60 (1.90 - 3.56) | – | – |
|  | Positive | 10/70 | 14.29 (7.82 - 24.66) | 3.74 (1.55 – 8.36) | **0.002** |
| *Paragonimus* spp. | Negative | 43/1473 | 2.92 (2.17 - 3.91) |  |  |
|  | Positive | 5/56 | 8.93 (3.73 - 19.86) |  |  |
| *T. trichiura* | Negative | 46/1487 | 3.09 (2.32 - 4.11) |  |  |
|  | Positive | 2/42 | 4.76 (1.17 - 17.40) |  |  |
| *A. lumbricoides* | Negative | 45/1485 | 3.03 (2.27 - 4.04) |  |  |
|  | Positive | 3/44 | 6.82 (2.18 - 19.34) |  |  |
| *S. mansoni* | Negative | 48/1510 | 3.18 (2.40 - 4.19) |  |  |
|  | Positive | 0/19 | 0.00 |  |  |

### ***T. trichiura***

| **Parasite** | **Status** | **Infected/Examined** | **Prevalence** | **aOR** | **p-value** |
| --- | --- | --- | --- | --- | --- |
| *O. volvulus* | Negative | 35/1341 | 2.61 (1.88 - 3.61) |  |  |
|  | Positive | 7/188 | 3.72 (1.78 - 7.62) |  |  |
| *M. perstans* | Negative | 38/1334 | 2.85 (2.08 - 3.89) |  |  |
|  | Positive | 4/195 | 2.05 (0.77 - 5.35) |  |  |
| *Loa loa* | Negative | 39/1380 | 2.83 (2.07 - 3.85) |  |  |
|  | Positive | 3/149 | 2.01 (0.65 - 6.08) |  |  |
| *D. dendriticum* | Negative | 40/1425 | 2.81 (2.06 - 3.81) |  |  |
|  | Positive | 2/104 | 1.92 (0.48 - 7.42) |  |  |
| *Capillaria* spp. | Negative | 40/1459 | 2.74 (2.02 - 3.72) |  |  |
|  | Positive | 2/70 | 2.86 (0.71 - 10.82) |  |  |
| *Paragonimus* spp. | Negative | 39/1473 | 2.65 (1.94 - 3.60) |  |  |
|  | Positive | 3/56 | 5.36 (1.72 - 15.49) |  |  |
| *Taenia* spp | Negative | 40/1481 | 2.70 (1.99 - 3.66) |  |  |
|  | Positive | 2/48 | 4.17 (1.03 - 15.40) |  |  |
| *A. lumbricoides* | Negative | 38/1485 | 2.56 (1.87 - 3.50) | – | – |
|  | Positive | 4/44 | 9.09 (3.41 - 22.06) | 3.35 (0.96 – 8.96) | **0.03** |
| *S. mansoni* | Negative | 40/1510 | 2.65 (1.95 - 3.59) | – | – |
|  | Positive | 2/19 | 10.53 (2.54 - 34.67) | 3.15 (0.48 – 11.70) | 0.14 |

### ***A. lumbricoides***

| **Parasite** | **Status** | **Infected/Examined** | **Prevalence** | **aOR** | **p-value** |
| --- | --- | --- | --- | --- | --- |
| *O. volvulus* | Negative | 36/1341 | 2.68 (1.94 - 3.70) |  |  |
|  | Positive | 8/188 | 4.26 (2.14 - 8.30) |  |  |
| *M. perstans* | Negative | 41/1334 | 3.07 (2.27 - 4.15) | – | – |
|  | Positive | 3/195 | 1.54 (0.50 - 4.68) | 0.43 (0.10 – 1.23) | 0.17 |
| *Loa loa* | Negative | 40/1380 | 2.90 (2.13 - 3.93) |  |  |
|  | Positive | 4/149 | 2.68 (1.01 - 6.96) |  |  |
| *D. dendriticum* | Negative | 37/1425 | 2.60 (1.89 - 3.56) | – | – |
|  | Positive | 7/104 | 6.73 (3.23 - 13.50) | 2.61 (1.04 – 5.68) | **0.024** |
| *Capillaria* spp. | Negative | 41/1459 | 2.81 (2.07 - 3.80) |  |  |
|  | Positive | 3/70 | 4.29 (1.38 - 12.56) |  |  |
| *Paragonimus* spp. | Negative | 40/1473 | 2.72 (2.00 - 3.68) |  |  |
|  | Positive | 4/56 | 7.14 (2.68 - 17.68) |  |  |
| *Taenia* spp | Negative | 41/1481 | 2.77 (2.04 - 3.74) |  |  |
|  | Positive | 3/48 | 6.25 (2.00 - 17.86) |  |  |
| *T. trichiura* | Negative | 40/1487 | 2.69 (1.98 - 3.65) | – | – |
|  | Positive | 4/42 | 9.52 (3.57 - 23.01) | 3.48 (1.00 – 9.33) | **0.025** |
| *S. mansoni* | Negative | 41/1510 | 2.72 (2.00 - 3.67) | – | – |
|  | Positive | 3/19 | 15.79 (5.01 - 39.99) | 5.25 (1.19 – 16.45) | **0.011** |

### ***S. mansoni***

| **Parasite** | **Status** | **Infected/Examined** | **Prevalence** | **aOR** | **p-value** |
| --- | --- | --- | --- | --- | --- |
| *O. volvulus* | Negative | 17/1341 | 1.27 (0.79 - 2.03) |  |  |
|  | Positive | 2/188 | 1.06 (0.26 - 4.17) |  |  |
| *M. perstans* | Negative | 16/1334 | 1.20 (0.74 - 1.95) |  |  |
|  | Positive | 3/195 | 1.54 (0.50 - 4.68) |  |  |
| *Loa loa* | Negative | 18/1380 | 1.30 (0.82 - 2.06) |  |  |
|  | Positive | 1/149 | 0.67 (0.09 - 4.64) |  |  |
| *D. dendriticum* | Negative | 17/1425 | 1.19 (0.74 - 1.91) |  |  |
|  | Positive | 2/104 | 1.92 (0.48 - 7.42) |  |  |
| *Capillaria* spp. | Negative | 18/1459 | 1.23 (0.78 - 1.95) |  |  |
|  | Positive | 1/70 | 1.43 (0.20 - 9.59) |  |  |
| *Paragonimus* spp. | Negative | 18/1473 | 1.22 (0.77 - 1.93) |  |  |
|  | Positive | 1/56 | 1.79 (0.25 - 11.81) |  |  |
| *Taenia* spp | Negative | 19/1481 | 1.28 (0.82 - 2.00) |  |  |
|  | Positive | 0/48 | 0.00 |  |  |
| *T. trichiura* | Negative | 17/1487 | 1.14 (0.71 - 1.83) | – | – |
|  | Positive | 2/42 | 4.76 (1.17 - 17.40) | 3.15 (0.48 – 11.70) | 0.14 |
| *A. lumbricoides* | Negative | 16/1485 | 1.08 (0.66 - 1.75) | – | – |
|  | Positive | 3/44 | 6.82 (2.18 - 19.34) | 4.90 (1.10 – 15.36) | **0.014** |

**Table B: Association between parasite groups**

### **Filaria**

| **Parasite group** | **Status** | **Infected/Examined** | **Prevalence** | **aOR** | **p-value** |
| --- | --- | --- | --- | --- | --- |
| Intest_Nema | Negative | 649/1982 | 32.74 (30.71 - 34.84) | – | – |
|  | Positive | 62/155 | 40.00 (32.56 - 47.93) | 1.37 (0.98 - 1.91) | 0.0658 |
| Trematode | Negative | 482/1399 | 34.45 (32.00 - 36.99) |  |  |
|  | Positive | 60/157 | 38.22 (30.93 - 46.08) |  |  |
| Cestode | Negative | 523/1503 | 34.80 (32.43 - 37.25) |  |  |
|  | Positive | 19/53 | 35.85 (24.06 - 49.63) |  |  |

### **Intestinal Nematode**

| **Parasite group** | **Status** | **Infected/Examined** | **Prevalence** | **aOR** | **p-value** |
| --- | --- | --- | --- | --- | --- |
| Filaria | Negative | 93/1426 | 6.52 (5.35 - 7.93) | – | – |
|  | Positive | 62/711 | 8.72 (6.86 - 11.03) | 1.27 (0.89 - 1.79) | 0.1834 |
| Trematode | Negative | 112/1399 | 8.01 (6.69 - 9.55) | – | – |
|  | Positive | 43/157 | 27.39 (20.96 - 34.91) | 3.89 (2.56 - 5.84) | **0.0000** |
| Cestode | Negative | 140/1503 | 9.31 (7.94 - 10.89) | – | – |
|  | Positive | 15/53 | 28.30 (17.75 - 41.93) | 2.66 (1.34 - 5.04) | **0.0036** |

### **Trematode**

| **Parasite group** | **Status** | **Infected/Examined** | **Prevalence** | **aOR** | **p-value** |
| --- | --- | --- | --- | --- | --- |
| Filaria | Negative | 97/1014 | 9.57 (7.90 - 11.54) |  |  |
|  | Positive | 60/542 | 11.07 (8.69 - 14.01) |  |  |
| Intest_Nema | Negative | 114/1401 | 8.14 (6.81 - 9.69) | – | – |
|  | Positive | 43/155 | 27.74 (21.24 - 35.34) | 3.92 (2.58 - 5.87) | **0.0000** |
| Cestode | Negative | 138/1503 | 9.18 (7.82 - 10.75) | – | – |
|  | Positive | 19/53 | 35.85 (24.06 - 49.63) | 4.43 (2.35 - 8.12) | **0.0000** |

### **Cestode**

| **Parasite group** | **Status** | **Infected/Examined** | **Prevalence** | **aOR** | **p-value** |
| --- | --- | --- | --- | --- | --- |
| Filaria | Negative | 34/1014 | 3.35 (2.40 - 4.66) |  |  |
|  | Positive | 19/542 | 3.51 (2.25 - 5.43) |  |  |
| Intest_Nema | Negative | 38/1401 | 2.71 (1.98 - 3.71) | – | – |
|  | Positive | 15/155 | 9.68 (5.91 - 15.46) | 2.64 (1.32 - 5) | **0.004** |
| Trematode | Negative | 34/1399 | 2.43 (1.74 - 3.38) | – | – |
|  | Positive | 19/157 | 12.10 (7.84 - 18.22) | 4.43 (2.35 - 8.12) | **0.000** |
