# Supplementary material for: Epidemiology of helminth infections in adults in rural rainforest communities of the Nkam-Wouri River Basin, Littoral Region of Cameroon: Implications for NTDs elimination using preventive chemotherapies
Source: PLoS Negl Trop Dis. 2026 Oct 9;20(10):e0014780. doi: 10.1371/journal.pntd.0014780 (PMC13686460; doi:10.1371/journal.pntd.0014780)
Supplement: Supplementary file 3 [file pntd.0014780.s003.docx]

# **S3 Appendix: Determinants of infection**

**Table A: Prevalence of helminth groups per socio-demographics and deworming history**

|  |  | ***Filariae*** | | ***Intestinal Nematodes*** | | ***Trematode*** | | **Cestodes** | |
| --- | --- | --- | --- | --- | --- | --- | --- | --- | --- |
| **Variables** | **Modalities** | **n/T** | **Prevalence (95%CI)** | **n/T** | **Prevalence (95%CI)** | **n/T** | **Prevalence (95%CI)** | **n/T** | **Prevalence (95%CI)** |
| Sex | Female | 245/1006 | 24.35 (21.80 - 27.11) | 67/1009 | 6.64 (5.26 - 8.35) | 64/704 | 9.09 (7.18 - 11.45) | 20/704 | 2.84 (1.84 - 4.36) |
|  | Male | 466/1131 | 41.20 (38.36 - 44.10) | 88/1131 | 7.78 (6.35 - 9.49) | 93/852 | 10.92 (8.99 - 13.20) | 33/852 | 3.87 (2.77 - 5.40) |
| Age | 18-49 | 335/1082 | 30.96 (28.27 - 33.78) | 71/1085 | 6.54 (5.22 - 8.18) | 78/800 | 9.75 (7.88 - 12.01) | 34/800 | 4.25 (3.05 - 5.89) |
|  | ≥50 | 376/1055 | 35.64 (32.80 - 38.58) | 84/1055 | 7.96 (6.47 - 9.76) | 79/756 | 10.45 (8.46 - 12.84) | 19/756 | 2.51 (1.61 - 3.91) |
| Education  Level | No formal education | 32/44 | 72.73 (57.64 - 83.94) | 3/44 | 6.82 (2.18 - 19.37) | 5/36 | 13.89 (5.81 - 29.65) | 1/36 | 2.78 (0.38 - 17.74) |
|  | Primary education | 84/226 | 37.17 (31.09 - 43.69) | 9/226 | 3.98 (2.08 - 7.50) | 12/209 | 5.74 (3.28 - 9.86) | 3/209 | 1.44 (0.46 - 4.38) |
|  | Secondary education | 26/180 | 14.44 (10.01 - 20.41) | 2/180 | 1.11 (0.28 - 4.36) | 5/169 | 2.96 (1.23 - 6.94) | 1/169 | 0.59 (0.08 - 4.12) |
|  | Higher education | 15/23 | 65.22 (43.76 - 81.88) | 1/23 | 4.35 (0.58 - 26.18) | 1/19 | 5.26 (0.69 - 30.67) | 0/19 | 0.00 |
| Health District | Loum | 84/183 | 45.90 (38.79 - 53.18) | 19/184 | 10.33 (6.67 - 15.64) | 26/129 | 20.16 (14.08 - 28.00) | 11/129 | 8.53 (4.77 - 14.78) |
|  | Manjo | 86/446 | 19.28 (15.88 - 23.22) | 36/447 | 8.05 (5.86 - 10.97) | 11/268 | 4.10 (2.28 - 7.27) | 5/268 | 1.87 (0.78 - 4.41) |
|  | Melong | 231/898 | 25.72 (22.97 - 28.69) | 54/898 | 6.01 (4.63 - 7.77) | 84/639 | 13.15 (10.74 - 16.00) | 19/639 | 2.97 (1.90 - 4.62) |
|  | Yabassi | 310/610 | 50.82 (46.85 - 54.78) | 46/611 | 7.53 (5.68 - 9.91) | 36/520 | 6.92 (5.03 - 9.45) | 18/520 | 3.46 (2.19 - 5.43) |
| BMI | Lean | 483/1259 | 38.36 (35.71 - 41.09) | 89/1260 | 7.06 (5.77 - 8.62) | 101/940 | 10.74 (8.92 - 12.89) | 34/940 | 3.62 (2.59 - 5.02) |
|  | Overweight | 175/593 | 29.51 (25.97 - 33.31) | 48/593 | 8.09 (6.15 - 10.58) | 40/428 | 9.35 (6.93 - 12.50) | 16/428 | 3.74 (2.30 - 6.02) |
|  | Obese | 42/260 | 16.15 (12.15 - 21.15) | 18/260 | 6.92 (4.40 - 10.73) | 14/176 | 7.95 (4.76 - 13.00) | 3/176 | 1.70 (0.55 - 5.17) |
| Last time point of deworming | Never | 261/697 | 37.45 (33.92 - 41.11) | 54/697 | 7.75 (5.98 - 9.98) | 56/505 | 11.09 (8.63 - 14.14) | 15/505 | 2.97 (1.80 - 4.87) |
|  | Unknown | 146/409 | 35.70 (31.19 - 40.47) | 29/409 | 7.09 (4.97 - 10.02) | 26/329 | 7.90 (5.43 - 11.36) | 13/329 | 3.95 (2.31 - 6.69) |
|  | <3 Months | 60/243 | 24.69 (19.66 - 30.52) | 13/244 | 5.33 (3.11 - 8.97) | 24/153 | 15.69 (10.72 - 22.37) | 7/153 | 4.58 (2.19 - 9.31) |
|  | 4 – 6 Months | 52/175 | 29.71 (23.39 - 36.92) | 16/175 | 9.14 (5.67 - 14.42) | 12/114 | 10.53 (6.06 - 17.67) | 0/114 | 0.00 |
|  | 7 – 12 Months | 176/571 | 30.82 (27.16 - 34.74) | 40/571 | 7.01 (5.18 - 9.41) | 36/426 | 8.45 (6.15 - 11.50) | 18/426 | 4.23 (2.68 - 6.61) |
| Duration in Community | 5-10 | 184/629 | 29.25 (25.82 - 32.94) | 39/630 | 6.19 (4.55 - 8.36) | 40/476 | 8.40 (6.22 - 11.26) | 15/476 | 3.15 (1.91 - 5.17) |
|  | 11-20 | 112/330 | 33.94 (29.02 - 39.23) | 30/330 | 9.09 (6.42 - 12.71) | 25/256 | 9.77 (6.68 - 14.07) | 13/256 | 5.08 (2.97 - 8.56) |
|  | 21-30 | 98/297 | 33.00 (27.87 - 38.56) | 26/297 | 8.75 (6.02 - 12.56) | 28/217 | 12.90 (9.05 - 18.07) | 8/217 | 3.69 (1.85 - 7.21) |
|  | 31-40 | 78/230 | 33.91 (28.07 - 40.29) | 14/231 | 6.06 (3.62 - 9.98) | 12/164 | 7.32 (4.19 - 12.47) | 5/164 | 3.05 (1.27 - 7.14) |
|  | 41-50 | 100/250 | 40.00 (34.09 - 46.21) | 18/251 | 7.17 (4.56 - 11.11) | 22/173 | 12.72 (8.51 - 18.58) | 6/173 | 3.47 (1.56 - 7.52) |
|  | >50 | 138/396 | 34.85 (30.31 - 39.68) | 28/396 | 7.07 (4.92 - 10.06) | 30/268 | 11.19 (7.93 - 15.57) | 6/268 | 2.24 (1.01 - 4.90) |
| Occupation | farming | 223/477 | 46.75 (42.30 - 51.26) | 25/477 | 5.24 (3.56 - 7.65) | 30/421 | 7.13 (5.02 - 10.02) | 9/421 | 2.14 (1.11 - 4.07) |
|  | Others | 48/120 | 40.00 (31.58 - 49.05) | 0/120 | 0.00 | 3/102 | 2.94 (0.94 - 8.79) | 0/102 | 0.00 |

***Table B: Prevalence of helminth groups per socio-demographics and deworming history***

|  |  | ***O. volvulus*** | | ***M. perstans*** | | ***L. loa*** | |
| --- | --- | --- | --- | --- | --- | --- | --- |
| **Variables** | **Modalities** | **n/T** | **Prevalence (95%CI)** | **n/T** | **Prevalence (95%CI)** | **n/T** | **Prevalence (95%CI)** |
| Sex | Female | 168/973 | 17.27 (15.02 - 19.77) | 81/1005 | 8.06 (6.53 - 9.91) | 58/1005 | 5.77 (4.49 - 7.40) |
|  | Male | 326/1098 | 29.69 (27.06 - 32.46) | 172/1128 | 15.25 (13.26 - 17.47) | 132/1128 | 11.70 (9.95 - 13.71) |
| Age | 18-49 | 213/1049 | 20.31 (17.98 - 22.85) | 127/1081 | 11.75 (9.96 - 13.81) | 100/1081 | 9.25 (7.66 - 11.13) |
|  | ≥50 | 281/1022 | 27.50 (24.84 - 30.32) | 126/1052 | 11.98 (10.15 - 14.08) | 90/1052 | 8.56 (7.01 - 10.41) |
| Education  Level | No formal education | 24/43 | 55.81 (40.69 - 69.93) | 11/43 | 25.58 (14.64 - 40.78) | 9/43 | 20.93 (11.16 - 35.81) |
|  | Primary education | 62/224 | 27.68 (22.19 - 33.93) | 43/226 | 19.03 (14.41 - 24.70) | 22/226 | 9.73 (6.48 - 14.37) |
|  | Secondary education | 21/179 | 11.73 (7.76 - 17.36) | 8/180 | 4.44 (2.23 - 8.66) | 4/180 | 2.22 (0.83 - 5.80) |
|  | Higher education | 14/23 | 60.87 (39.73 - 78.59) | 1/23 | 4.35 (0.58 - 26.18) | 2/23 | 8.70 (2.11 - 29.64) |
| Health District | Loum | 56/146 | 38.36 (30.80 - 46.52) | 48/183 | 26.23 (20.35 - 33.10) | 8/183 | 4.37 (2.20 - 8.51) |
|  | Manjo | 79/441 | 17.91 (14.60 - 21.78) | 3/445 | 0.67 (0.22 - 2.07) | 10/445 | 2.25 (1.21 - 4.13) |
|  | Melong | 187/888 | 21.06 (18.50 - 23.87) | 35/898 | 3.90 (2.81 - 5.38) | 33/898 | 3.67 (2.62 - 5.13) |
|  | Yabassi | 172/596 | 28.86 (25.36 - 32.63) | 167/607 | 27.51 (24.10 - 31.21) | 139/607 | 22.90 (19.72 - 26.42) |
| BMI | Lean | 349/1217 | 28.68 (26.20 - 31.29) | 178/1255 | 14.18 (12.36 - 16.23) | 118/1255 | 9.40 (7.91 - 11.15) |
|  | Overweight | 108/579 | 18.65 (15.68 - 22.04) | 60/593 | 10.12 (7.93 - 12.82) | 57/593 | 9.61 (7.48 - 12.26) |
|  | Obese | 27/253 | 10.67 (7.41 - 15.13) | 14/260 | 5.38 (3.21 - 8.90) | 15/260 | 5.77 (3.50 - 9.36) |
| Last time point of deworming | Never | 186/673 | 27.64 (24.39 - 31.14) | 80/696 | 11.49 (9.33 - 14.09) | 64/696 | 9.20 (7.26 - 11.58) |
|  | Unknown | 100/397 | 25.19 (21.16 - 29.70) | 49/408 | 12.01 (9.19 - 15.55) | 52/408 | 12.75 (9.84 - 16.35) |
|  | <3 Months | 39/231 | 16.88 (12.57 - 22.29) | 27/243 | 11.11 (7.72 - 15.73) | 14/243 | 5.76 (3.44 - 9.50) |
|  | 4 – 6 Months | 36/170 | 21.18 (15.66 - 27.99) | 21/175 | 12.00 (7.94 - 17.73) | 11/175 | 6.29 (3.51 - 11.01) |
|  | 7 – 12 Months | 118/559 | 21.11 (17.92 - 24.70) | 72/570 | 12.63 (10.14 - 15.62) | 47/570 | 8.25 (6.25 - 10.81) |
| Duration in Community | 5-10 | 111/597 | 18.59 (15.67 - 21.92) | 82/628 | 13.06 (10.64 - 15.93) | 64/628 | 10.19 (8.05 - 12.82) |
|  | 11-20 | 81/322 | 25.16 (20.71 - 30.19) | 40/330 | 12.12 (9.01 - 16.11) | 33/330 | 10.00 (7.19 - 13.74) |
|  | 21-30 | 66/293 | 22.53 (18.09 - 27.68) | 30/296 | 10.14 (7.17 - 14.14) | 28/296 | 9.46 (6.61 - 13.37) |
|  | 31-40 | 60/223 | 26.91 (21.48 - 33.12) | 24/230 | 10.43 (7.08 - 15.11) | 20/230 | 8.70 (5.67 - 13.11) |
|  | 41-50 | 69/244 | 28.28 (22.97 - 34.27) | 36/249 | 14.46 (10.60 - 19.41) | 21/249 | 8.43 (5.56 - 12.60) |
|  | >50 | 107/388 | 27.58 (23.35 - 32.25) | 40/395 | 10.13 (7.51 - 13.52) | 24/395 | 6.08 (4.10 - 8.91) |
| Occupation | farming | 166/472 | 35.17 (30.98 - 39.60) | 101/477 | 21.17 (17.73 - 25.08) | 53/477 | 11.11 (8.58 - 14.27) |
|  | Others | 38/119 | 31.93 (24.15 - 40.88) | 11/119 | 9.24 (5.17 - 15.98) | 10/119 | 8.40 (4.56 - 14.97) |

***Table C. Association between helminth group prevalence and socio-demographics and deworming history***

|  |  | **Filariae** | | **Intestinal Nematodes** | | **Trematodes** | | **Cestodes** | |
| --- | --- | --- | --- | --- | --- | --- | --- | --- | --- |
| **Variables** | **Modalities** | **aOR (95%CI)** | **p-value** | **aOR (95%CI)** | **p-value** | **aOR (95%CI)** | **p-value** | **aOR (95%CI)** | **p-value** |
| Sex | Female | – | – | – | – | – | – | – | – |
|  | Male | 0.62 (0.34 – 1.10) | 0.106 | – | – | – | – | – | – |
| Age | 18-49 | – | – | – | – | – | – | – | – |
|  | ≥50 | 1.35 (1.06 – 1.71) | 0.014 | – | – | – | – | 0.62 (0.34 – 1.10) | 0.106 |
| Highest Education  Level | No formal education | – | – | – | – | – | – | – | – |
|  | Primary education | – | – | – | – | – | – | – | – |
|  | Secondary education | – | – | – | – | – | – | – | – |
|  | Higher education | – | – | – | – | – | – | – | – |
| Health District | Loum | – | – | – | – | – | – | – | – |
|  | Manjo | 0.29 (0.19 – 0.44) | <0.001 | – | – | 0.15 (0.06 – 0.32) | <0.001 | 0.22 (0.07 – 0.62) | 0.006 |
|  | Melong | 0.41 (0.29 – 0.58) | <0.001 | – | – | 0.62 (0.38 – 1.03) | 0.054 | 0.33 (0.15 – 0.75) | 0.006 |
|  | Yabassi | 1.73 (1.21 – 2.49) | 0.003 | – | – | 0.3 (0.17 – 0.53) | <0.001 | 0.36 (0.16 – 0.81) | 0.011 |
| BMI | Lean | – | – | – | – | – | – | – | – |
|  | Overweight | 0.51 (0.39 – 0.66) | <0.001 | – | – | – | – | – | – |
|  | Obese | 0.86 (0.69 – 1.06) | 0.164 | – | – | – | – | – | – |
| Last time point of deworming | Never | – | – | – | – | – | – | – | – |
|  | Unknown | 0.72 (0.54 – 0.97) | 0.028 | – | – | – | – | 1.37 (0.62 – 3.02) | 0.427 |
|  | <3 Months | 0.5 (0.34 – 0.71) | <0.001 | – | – | – | – | 1.59 (0.59 – 3.91) | 0.332 |
|  | 4 – 6 Months | 0.62 (0.41 – 0.91) | 0.017 | – | – | – | – | 0 (0.00 – 35646.23) | 0.98 |
|  | 7 – 12 Months | 0.66 (0.51 – 0.85) | 0.001 | – | – | – | – | 1.7 (0.83 – 3.52) | 0.146 |
| Duration in Community | 5-10 | – | – | – | – | – | – | – | – |
|  | 11-20 | 1.54 (1.11 – 2.12) | 0.009 | – | – | – | – | – | – |
|  | 21-30 | 1.72 (1.23 – 2.41) | 0.002 | – | – | – | – | – | – |
|  | 31-40 | 2.07 (1.42 – 3.02) | <0.001 | – | – | – | – | – | – |
|  | 41-50 | 2.33 (1.63 – 3.33) | <0.001 | – | – | – | – | – | – |
|  | >50 | 1.7 (1.19 – 2.44) | 0.004 | – | – | – | – | – | – |
| Occupation | farming | – | – | – | – | – | – | – | – |
|  | Others | – | – | – | – | – | – | – | – |

***Table D. Association between filariasis prevalence and socio-demographics and deworming history***

|  |  | ***O. volvulus*** |  | ***M. perstans*** |  | ***L. loa*** |  |
| --- | --- | --- | --- | --- | --- | --- | --- |
| **Variables** | **Modalities** | **aOR (95%CI)** | **p-value** | **aOR (95%CI)** | **p-value** | **aOR (95%CI)** | **p-value** |
| Sex | Female | – | – | – | – | – | – |
|  | Male | – | – | – | – | – | – |
| Age | 18-49 | ­– | – |  |  |  |  |
|  | ≥50 | 1.56 (0.84 – 2.88) | 0.155 | 2.18 (1.16 – 4.17) | **0.017** | – | – |
| Education  Level | No formal education | – | **–** | – | – | – | – |
|  | Primary education | 0.42 (0.18 – 0.95) | **0.038** | 1.38 (0.59 – 3.45) | 0.47 | 0.53 (0.21 – 1.42) | 0.19 |
|  | Secondary education | 0.17 (0.07 – 0.41) | **<0.001** | 0.2 (0.07 – 0.60) | **0.004** | 0.06 (0.01 – 0.21) | **<0.001** |
|  | Higher education | 3.58 (0.93 – 14.75) | 0.069 | 0.26 (0.01 – 1.81) | 0.241 | 0.65 (0.08 – 3.48) | 0.634 |
| Health District | Loum | – | – |  |  |  |  |
|  | Manjo | 0.06 (0.02 – 0.17) | **<0.001** | 0 (0.00 – 26392.80) | 0.983 | 0.17 (0.01 – 1.42) | 0.137 |
|  | Melong | 0.24 (0.10 – 0.53) | **0.001** | 0.1 (0.04 – 0.26) | **<0.001** | 0.79 (0.18 – 4.03) | 0.758 |
|  | Yabassi | 0.77 (0.35 – 1.70) | 0.522 | 0.78 (0.37 – 1.66) | 0.514 | 6.52 (2.09 – 28.82) | **0.004** |
| BMI | Lean | – | – | – | – | – | – |
|  | Overweight | 0.53 (0.26 – 1.00) | 0.065 | – | – | – | – |
|  | Obese | 1.39 (0.77 – 2.54) | 0.275 | – | – | – | – |
| Last time point of deworming | Never | – | – | – | – | – | – |
|  | Unknown | 0.42 (0.20 – 0.88) | **0.023** | – | – | – | – |
|  | <3 Months | 0.58 (0.22 – 1.43) | 0.249 | – | – | – | – |
|  | 4 – 6 Months | 1.06 (0.40 – 2.72) | 0.897 | – | – | – | – |
|  | 7 – 12 Months | 0.39 (0.19 – 0.78) | **0.009** | – | – | – | – |
| Duration in Community | 0-10 | – | – | – | – | – | – |
|  | 11-20 | 2.45 (1.06 – 5.73) | **0.037** | – | – | – | – |
|  | 21-30 | 1.58 (0.60 – 4.07) | 0.344 | – | – | – | – |
|  | 31-40 | 6.41 (2.43 – 17.61) | **<0.001** | – | – | – | – |
|  | 41-50 | 2.67 (1.08 – 6.63) | **0.033** | – | – | – | – |
|  | >50 | 2.48 (0.95 – 6.64) | 0.065 | – | – | – | – |
| Occupation | farming | – | – | – | – | – | – |
|  | Others | – | – | – | – | – | – |

***Table E: Determinants of coinfection among infected participants***

| **Predictors** | **Modalities** | **nCo-inf/nInfected** | **Perc_Coinf** | ***X^2^*p** | **aOR (95% CI)** | **p.value** |
| --- | --- | --- | --- | --- | --- | --- |
| Sex | Female | 105/334 | 31.44 (26.67 - 36.63) | **0.021** | – | – |
|  | Male | 218/557 | 39.14 (35.16 - 43.27) | - | 1.18 (0.69 - 2.04) | 0.542 |
| Age | 18-49 | 152/434 | 35.02 (30.67 - 39.64) | 0.457 | - | - |
|  | 50+ | 171/457 | 37.42 (33.09 - 41.96) | - | - | - |
| Health District | Loum | 50/105 | 47.62 (38.21 - 57.20) | **<0.001** | – | – |
|  | Manjo | 22/122 | 18.03 (12.15 - 25.92) | - | 0.22 (0.07 - 0.59) | **0.004** |
|  | Melong | 88/306 | 28.76 (23.95 - 34.10) | - | 0.41 (0.19 - 0.88) | **0.023** |
|  | Yabassi | 163/358 | 45.53 (40.42 - 50.73) | - | 0.99 (0.52 - 1.9) | 0.979 |
| Education | No formal | 14/32 | 43.75 (27.56 - 61.39) | 0.170 | - | - |
|  | Primary | 42/88 | 47.73 (37.41 - 58.25) | - | - | - |
|  | Secondary | 10/27 | 37.04 (20.87 - 56.74) | - | - | - |
|  | Higher | 3/16 | 18.75 (5.88 - 46.01) | - | - | - |
| Obesity | Lean | 217/581 | 37.35 (33.50 - 41.37) | 0.387 | - | - |
|  | Overweight | 86/232 | 37.07 (31.07 - 43.49) | - | - | - |
|  | Obese | 19/66 | 28.79 (19.10 - 40.90) | - | - | - |
| Last time point of Treatment | Never | 108/318 | 33.96 (28.95 - 39.36) | 0.657 | - | - |
|  | Unknown | 73/180 | 40.56 (33.60 - 47.91) | - | - | - |
|  | <3M | 32/82 | 39.02 (29.04 - 50.02) | - | - | - |
|  | <6M | 24/68 | 35.29 (24.83 - 47.39) | - | - | - |
|  | <12M | 81/223 | 36.32 (30.25 - 42.86) | - | - | - |
| Duration in  Community | 0-10 | 91/236 | 38.56 (32.54 - 44.95) | 0.405 | - | - |
|  | 11-20 | 59/142 | 41.55 (33.70 - 49.85) | - | - | - |
|  | 21-30 | 49/129 | 37.98 (29.99 - 46.69) | - | - | - |
|  | 31-40 | 32/94 | 34.04 (25.15 - 44.23) | - | - | - |
|  | 41-50 | 39/121 | 32.23 (24.48 - 41.10) | - | - | - |
|  | >50 | 53/168 | 31.55 (24.95 - 38.99) | - | - | - |
| Occupation | Farmers | 109/229 | 47.60 (41.16 - 54.11) | **<0.001** | – | – |
|  | Others | 10/50 | 20.00 (11.01 - 33.55) | - | 0.3 (0.13 - 0.61) | **0.002** |

*X^2^*p: p-value from the Pearson’s chi square test; aOR 95% CI: Adjusted odds ratio and associated 95% confidence interval
