# Supplementary material for: Epidemiology of helminth infections in adults in rural rainforest communities of the Nkam-Wouri River Basin, Littoral Region of Cameroon: Implications for NTDs elimination using preventive chemotherapies
Source: PLoS Negl Trop Dis. 2026 Oct 9;20(10):e0014780. doi: 10.1371/journal.pntd.0014780 (PMC13686460; doi:10.1371/journal.pntd.0014780)
Supplement: Supplementary file 4 [file pntd.0014780.s004.docx]

|  |  |  | **Age Five Numbers Statistics** | | | | | **Age groups n (%)** | | **Gender n (%)** | |
| --- | --- | --- | --- | --- | --- | --- | --- | --- | --- | --- | --- |
| **Parasitic group** | **Parasitic species** | **N infected** | **Min** | **Q1** | **Q2** | **Q3** | **Max** | **18 – 49** | **≥50** | **Males** | **Females** |
| Filariae | *O. volvulus* | 494 | 18 | 40.0 | 52.00 | 60.00 | 74 | 213 (43.12) | 281 (56.88) | 326 (65.99) | 168 (34.01) |
| Filariae | *M. perstans* | 253 | 18 | 37.00 | 49.00 | 59.00 | 74 | 127 (50.2) | 126 (49.8) | 172 (67.98) | 81 (32.02) |
| Filariae | *L. loa* | 190 | 18 | 38.00 | 48.00 | 57.00 | 60 | 100 (52.63) | 90 (47.37) | 132 (69.47) | 58 (30.53) |
| Trematode | *D. dendriticum* | 107 | 18 | 40.00 | 50.00 | 60.00 | 60 | 50 (46.73) | 57 (53.27) | 60 (56.07) | 47 (43.93) |
| Intest_Nema | *Capillaria* spp. | 71 | 18 | 39.00 | 50.00 | 56.50 | 60 | 35 (49.3) | 36 (50.7) | 36 (50.7) | 35 (49.3) |
| Trematode | *Paragonimus* spp. | 54 | 21 | 36.50 | 52.00 | 58.00 | 60 | 21 (38.89) | 33 (61.11) | 34 (62.96) | 20 (37.04) |
| Cestode | *Taenia* spp. | 48 | 18 | 30.00 | 42.50 | 53.50 | 60 | 32 (66.67) | 16 (33.33) | 31 (64.58) | 17 (35.42) |
| Intest_Nema | *A. lumbricoides* | 45 | 19 | 35.00 | 52.00 | 60.00 | 61 | 18 (40) | 27 (60) | 30 (66.67) | 15 (33.33) |
| Intest_Nema | *T. trichiura* | 42 | 20 | 40.50 | 50.50 | 60.00 | 60 | 18 (42.86) | 24 (57.14) | 26 (61.9) | 16 (38.1) |
| Trematode | *S. mansoni* | 18 | 18 | 28.00 | 33.50 | 45.00 | 60 | 14 (77.78) | 4 (22.22) | 11 (61.11) | 7 (38.89) |
| Intest_Nema | *E. vermicularis* | 6 | 27 | 29.75 | 35.00 | 36.50 | 53 | 5 (83.33) | 1 (16.67) | 3 (50) | 3 (50) |
| Cestode | *D. caninum* | 3 | 30 | 44.00 | 58.00 | 59.00 | 60 | 1 (33.33) | 2 (66.67) | 1 (33.33) | 2 (66.67) |
| Trematode | *Fasciola* spp. | 3 | 24 | 36.00 | 48.00 | 49.00 | 50 | 2 (66.67) | 1 (33.33) | 0 (0) | 3 (100) |
| Cestode | *H. nana* | 2 | 33 | 37.50 | 42.00 | 46.50 | 51 | 1 (50) | 1 (50) | 1 (50) | 1 (50) |
| Intest_Nema | *S. stercoralis* | 2 | 48 | 49.75 | 51.50 | 53.25 | 55 | 1 (50) | 1 (50) | 0 (0) | 2 (100) |
| Intest_Nema | *Trichostrongylus* spp*.* | 2 | 45 | 47.50 | 50.00 | 52.50 | 55 | 1 (50) | 1 (50) | 1 (50) | 1 (50) |
| Cestode | *D. latum* | 1 | 44 | 44.00 | 44.00 | 44.00 | 44 | 1 (100) | 0 (0) | 0 (0) | 1 (100) |
| Intest_Nema | Hookworms | 1 | 59 | 59.00 | 59.00 | 59.00 | 59 | 0 (0) | 1 (100) | 1 (100) | 0 (0) |
| Trematode | *Clonorchis* spp. | 1 | 60 | 60.00 | 60.00 | 60.00 | 60 | 0 (0) | 1 (100) | 0 (0) | 1 (100) |
| Trematode | *Opisthorchis* spp. | 1 | 22 | 22.00 | 22.00 | 22.00 | 22 | 1 (100) | 0 (0) | 1 (100) | 0 (0) |

S4-Appendix. Figure A data set
